# Novel Modular Rhodopsins from Green Algae Hold Great Potential for Cellular Optogenetic Modulation Across the Biological Model Systems

**DOI:** 10.3390/life10110259

**Published:** 2020-10-28

**Authors:** Mayanka Awasthi, Kumari Sushmita, Manish Singh Kaushik, Peeyush Ranjan, Suneel Kateriya

**Affiliations:** 1 Department of Cell Biology and Molecular Genetics, University of Maryland, College Park, MD 20742, USA; awasthi9@umd.edu; 2 Laboratory of Optobiology, School of Biotechnology, Jawaharlal Nehru University, New Delhi 110067, India; sushmi59_sbt@jnu.ac.in (K.S.); manish13587@gmail.com (M.S.K.)

**Keywords:** enzyme-rhodopsin, channelrhodopsins, optogenetics, two-component system, cyclase, phosphodiesterase

## Abstract

Light-gated ion channel and ion pump rhodopsins are widely used as optogenetic tools and these can control the electrically excitable cells as (1) they are a single-component system i.e., their light sensing and ion-conducting functions are encoded by the 7-transmembrane domains and, (2) they show fast kinetics with small dark-thermal recovery time. In cellular signaling, a signal receptor, modulator, and the effector components are involved in attaining synchronous regulation of signaling. Optical modulation of the multicomponent network requires either receptor to effector encoded in a single ORF or direct modulation of the effector domain through bypassing all upstream players. Recently discovered modular rhodopsins like rhodopsin guanylate cyclase (RhoGC) and rhodopsin phosphodiesterase (RhoPDE) paves the way to establish a proof of concept for utilization of complex rhodopsin (modular rhodopsin) for optogenetic applications. Light sensor coupled modular system could be expressed in any cell type and hence holds great potential in the advancement of optogenetics 2.0 which would enable manipulating the entire relevant cell signaling system. Here, we had identified 50 novel modular rhodopsins with variant domains and their diverse cognate signaling cascades encoded in a single ORF, which are associated with specialized functions in the cells. These novel modular algal rhodopsins have been characterized based on their sequence and structural homology with previously reported rhodopsins. The presented novel modular rhodopsins with various effector domains leverage the potential to expand the optogenetic tool kit to regulate various cellular signaling pathways across the diverse biological model systems.

## 1. Introduction

The photobehavioural responses of many organisms are mediated by the rhodopsin-based photoreceptor(s) that are distributed across almost all clades of life. Rhodopsins are seven-transmembrane helical proteins that use retinal as a chromophore. Based on the isoforms of the retinal bound in the ground state, rhodopsins are classified into two broad categories i.e., Type I or microbial type (MTR) and Type II or animal-type rhodopsins (ATR). MTRs are widely distributed across all kingdoms of life and perform diverse physiological functions, such as the light-activated ion pump Bacteriorhodopsin (BR) [1] and Halorhodopsin (HR) [2], light-gated channel Channelrhodopsins (ChR1 & ChR2) [3,4], and sensory photoreceptors (SRI & II) [5]. Light-gated ion pumps and channels cause alterations in the membrane potential in a light-dependent manner whereas sensory rhodopsins mediate downstream signaling. SRI and II in halobacteria communicate with the flagellar motor via the transducer proteins HtrI and HtrII, respectively [5]. 

ATR or type II rhodopsins are broadly classified as vertebrate and invertebrate rhodopsins based on variation in their amino acid sequences [6]. The ATRs (both vertebrate and invertebrate) mediate the downstream signaling cascade through the G-protein that involves multiple steps and protein complexes. Both the ATRs as well as the SRs of MTRs are multi-component systems that require a series of protein complexes to mediate the light-activated signaling. This limits their use as an optogenetic tool for regulating intracellular signaling processes. The success of MTRs as an optogenetic tool is mainly attributed to their property that both the light-sensing and the ion channel activity of the Channelrhodopsins (ChRs) are encoded in a single protein. Recent advancements in the genome database have led to the discovery of many new MTRs that are directly coupled to effector domains, e.g., two-component system and cyclase in enzyme-rhodopsins [7,8]. This structural diversity imparts great precision, fast kinetics, and low off-target effects that provide an edge to the MTRs to target and regulate specific cellular processes simply by illumination. cAMP and cGMP, the key modulators of cell signaling, are the secondary messengers that regulate many cellular, metabolic, and developmental processes. However, it is difficult to target/modulate cGMP and cAMP levels precisely in specific cell types with spatiotemporal resolution using the animal-type rhodopsin signaling cascade because of the involvement of many players in the cascade. In addition, pharmacological targeting has the limitation of specificity and temporal issues at the cellular level. 

Enzyme-rhodopsins (Rhodopsin phosphodiesterase; RhoPDE and Rhodopsin guanylate cyclase; RhoGC) have emerged as promising optogenetic tools for the precise and non-invasive spatiotemporal control of cyclic nucleotide signaling pathways. The heterologous expression of RhoPDE [9,10] from *Salpingoeca rosetta* in *Xenopus* oocyte and HEK293 cell lines demonstrated the light-activated cGMP and cAMP-phosphodiesterase activity [11]. Similarly, RhoGC [12,13] isolated from fungi *Blastocladiella emersonii* and *Catenaria anguillulae* when expressed in various mammalian cell lines, could generate substantial cGMPs [14,15]. Furthermore, the existing rhodopsins can be modified via mutations or new sequences can be searched to overcome the current shortcomings of the optogenetic field by means of the optogenetic toolbox v2.0 [16]. Consequently, significant interest has developed towards the identification, characterization, and testing of novel modular rhodopsins [7,17,18] as optogenetic tool candidates for tweaking the cell signaling processes. The identified modular rhodopsins coupled with other domains in a single ORF have shown the potential to overcome the limitation of SRs to be used as an optogenetic tool. Characterizing the physiological role of the existing and newly identified multidomain rhodopsins is tempting but limited because of their large transcript size, poor heterologous expression of the transmembrane domain, and lack of the established functional assays for these modular rhodopsins. Recently, we have identified several new modular rhodopsins from different algae [7]. In the present study, we have identified fifty new modular rhodopsins and ChRs fused with new domains that were previously unknown and analyzed their evolutionary pattern, sequence homology as well as the structural and functional potential of these domains coupled to rhodopsin (based on available experimental evidence). We have also investigated the diversity of multidomain rhodopsins and the recruitment of signaling components in a single ORF in relation to its prokaryotic counterpart. This extensive analysis of MTRs defines a future roadmap towards the involvement of modular rhodopsin-based photoreceptors in the photophysiological response of the relevant organism. Evolutionary pattern analysis of the MTRs suggests the evolution of multi-domain rhodopsins in the microalgal system after the evolution of the ChRs with extended C-terminus of unknown function by lateral gene transfer. Moreover, these novel modular rhodopsins with different effector domains strengthen the potential to expand the optogenetics tool kit 2.0 to regulate various cellular signaling pathways across a multitude of biological model systems.

## 2. Materials and Methods

### 2.1. Identification of Rhodopsin Domain, Homology and Structural Analysis

Extensive genome database search for MTRs and modular rhodopsins were performed on the JGI genome database, metagenome database, and NCBI portal using BR and *Chlamydomonas* rhodopsin as a template. The rhodopsin identity, sequence accession number, homology, conserved domains, are summarized in Table S1. Multiple sequence alignment was performed using the Clustal_ X program [19] and BioEdit (http://www.mbio.ncsu.edu/bioedit/bioedit.html). All color editing was done by using the BioEdit program. The rhodopsin domains of new MTRs were identified by sequence alignment with canonical rhodopsins, analyzed with conserved domain architecture retrieval tool (CDART) [20] and conserved domain database [21] programs. The rhodopsins with conserved seven transmembrane helices and retinal binding motif in the seventh helix were considered for further analysis. The number indicating the position of amino acid is referred with respect to BR unless mentioned in the text. 

### 2.2. Evolutionary Analysis of Rhodopsin Domains of Modular Proteins

Molecular evolutionary analysis of typical MTR and rhodopsin domains (helices 1−7) of modular proteins was performed computationally with protein sequences. Multiple sequence alignment of the rhodopsin domain was done on Clustal X 2.0 [19]. Phylogenetic analysis was performed by the Neighbor-joining (NJ) method using MEGA X [22] with a thousand bootstrap replicates. The same was also verified by the maximum likelihood (ML) method on MEGA X. The topology was viewed by MEGA X as well as by tree view and NJ plot [23]. 

### 2.3. Protein-Protein Interaction Analysis of Novel Domains from Modular Algal Rhodopsins

The interactomes of effector domain(s) associated with ChRs, i.e., FimV, MED15, and UL36, were constructed. The interacting partners for each of the effector domains were predicted using the String version 11 [24] and the output was further used to generate the network by employing Cytoscape 3.7.2 [25]. 

## 3. Results and Discussion

### 3.1. Microbial Rhodopsins With Modular Domain Organization

Mining the genome database of the organisms from diverse taxa and strata revealed the presence of MTRs in various organisms inhabiting diverse habitats from freshwater to terrestrial environments. The phototactic green alga *C. reinhardtii* is an excellent system to study and learn various aspects of cell biology ranging from the photobehavioural responses (especially ChR-mediated) to photosynthesis, cilia biology, intraflagellar transport to vesicle, and membrane-bound trafficking and dynamics [26,27]. Steady progress on unraveling the photobehavioural response in *Chlamydomonas* led to the early discovery of modular rhodopsins in this green alga but since then very few have been reported in other organisms.

**Figure 1:**
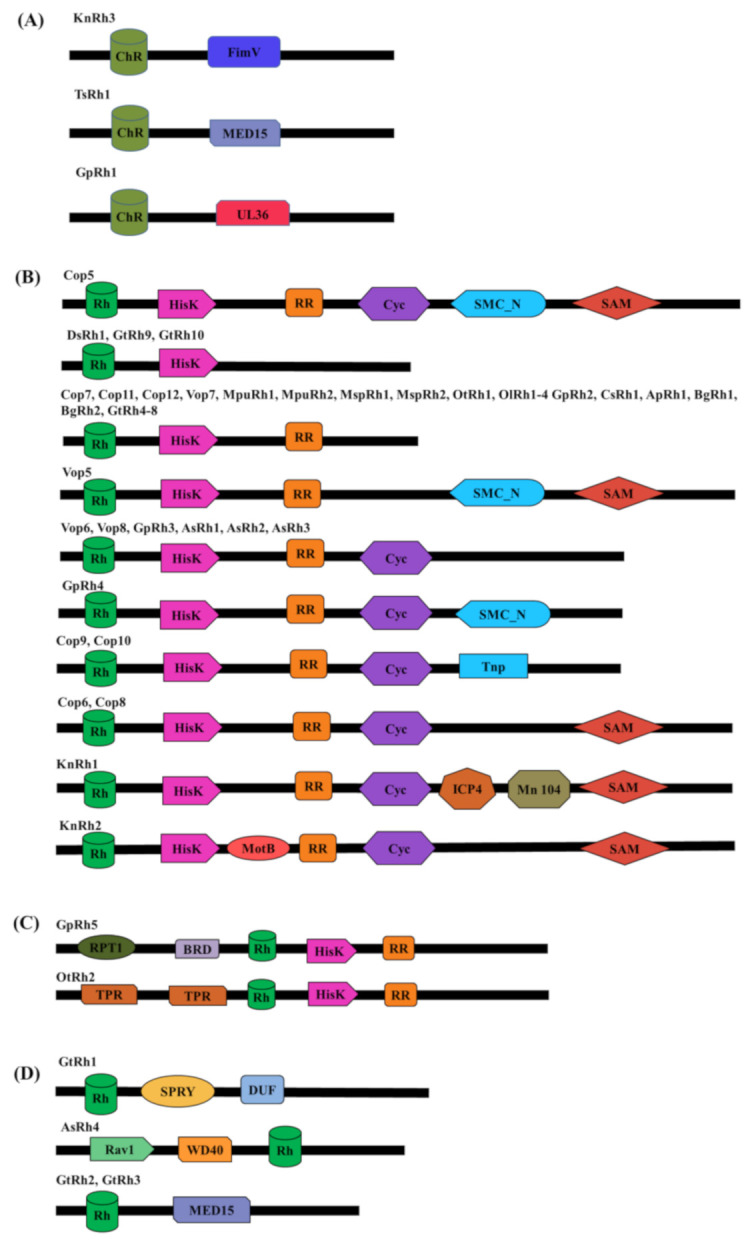
Schematic representation of domains present in modular microbial type rhodopsins. The schematic representation shows rhodopsin with modular domain(s), the black line represents full-length protein, and domains are depicted by geometric structures (Figure not to scale). (**A**) Domain organization of modular Channelrhodopsins (ChRs). ChR coupled with FimV (peptidoglycan binding protein), MED15 (mediator of RNA polymerase transcription factor subunit 15), and UL36 (large tegument protein) were found in three different algae. (**B**) Rhodopsin coupled HisK and RR form the largest group of modular domains and others have additional unique effector domains like cyclase (Cyc), sterile alpha subunit (SAM), structural maintenance of chromosome_N-terminus (SMC_N), transposase (Tnp2), major viral transcription factor ICP4 homolog (ICP4), 104kDa microneme/rhoptry (Mn 104) and bacterial flagellar motor protein (MotB). (**C**) Modular rhodopsin with rhodopsin preceded by unique domain at N-terminus; ATP-dependent 26S proteasome (RPT1) and bromodomain (BRD) in GpRh5 and tricopeptide (TPR) in OtRh2. (**D**) Modular rhodopsin lacking HisK and RR; GtRh1 possesses Spore lysis A and Ryanodine receptor (SPRY) domain that regulates innate and adaptive immune response and domain of unknown function (DUF), GtRh2 and 3 possess MED15. AsRh1 possesses regulator of V-ATPase of vacuolar membrane protein 1 (Rav1) and WD40 at N-terminus.

Here, we have identified new microbial modular ChRs (Figure 1A and Table 1A,B) and modular sensory-type rhodopsins (Figure 1B–D and Table 2A,B) across different taxa and analyzed their critical features that segregate MTRs from other seven-transmembrane protein families. 

### 3.2. Modular Channelrhodopsins and Their Optogenetic Potential 

**Figure 2:**
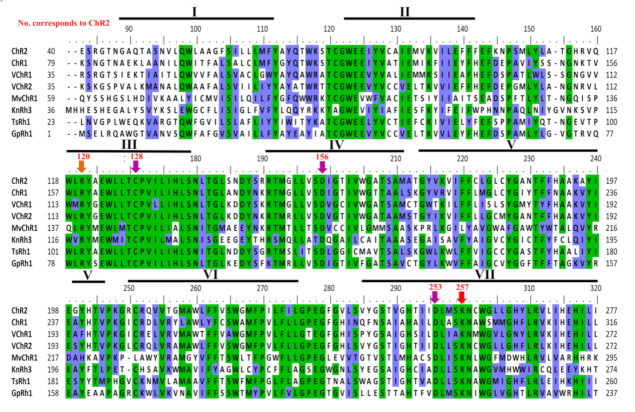
Comparison of novel channelrhodopsins and mapping of the important amino acid residues: Modular ChRs (KnRh3, TsRh1, and GpRh1) were aligned with other ChRs (ChR1 and ChR2 Figure 1. & VChR2 from *V. carteri*, MvChR1 from *M. viride*. Helices 1–7 are depicted by a black bar and marked in roman numbers. Retinal binding lysine is marked by the red arrow; proton acceptor/donor and cysteine hydrogen-bonded to proton donor (DC pair) are marked by the pink arrow; arginine is important for primary translocation of the proton is marked by an orange arrow.

Our targeted search for the modular ChR yielded three modular ChRs as shown in Figure 1A. These are KnRh3 from *Klebsormidium nitens,* TsRh1 from *Tetraselmis subcordiformis*, and GpRh1 from *Gonium pectorale.* KnRh3 is coupled with the peptidoglycan binding protein, FimV, whereas TsRh1 is the blue-shifted ChR for which the rhodopsin domain has been characterized [28]. TsRh1 is coupled with the mediator subunit, MED15 (Mediator of RNA polymerase II subunit 15) [28], however its modular nature has not been characterized and discussed. GpRh1 from *Gonium pectorale* is coupled with UL36 (large tegument protein). The optogenetic potentials of these modular domains (FimV, MED15, and UL36) are summarized in Table 1A. The Rhodopsin domains of KnRh3, TsRh1, and GpRh1 were aligned with well-characterized ChRs taken as the reference for sequence analysis (Figure 2). The conserved residues essential for photocycle are marked in Figure 2, and the same have been analyzed for four main functionalities namely: (1) retinal-binding lysine, (2) counter ion/proton acceptor of retinal Schiff base (RSB,) (3) stabilization of proton acceptor and, (4) DC-gate present in helix 3 and 4. Based on these amino acid residues, we evaluated the rhodopsin domains and summarized the details in Table 1B and Table 2B for modular ChRs and modular sensory-type rhodopsins, respectively.

All the three ChRs have the conserved seven transmembrane domains and the lysine motif at the seventh helix that forms a covalent linkage with retinal (Figure 2 and Table 1B). Asp253 (in ChR2) accepts the proton from the RSB during deprotonation and Asp156 (in ChR2) donates the proton to the RSB during re-protonation. Both these sites are conserved in modular ChRs (Figure 2 and Table 1B). Arg82 (in BR) stabilizes the negatively charged proton acceptor Asp85 (in BR) and is hydrogen bonded to Tyr83 via water 405 in M state and together they play a primary role during deprotonation of RSB. The corresponding position in ChR2 (Arg120) is hydrogen-bonded to E253 (proton acceptor) and is the core of the extracellular gate participating in ion movement [29]. This site is highly conserved among MTRs including modular ChRs (Figure 2 and Table 1B). Asp156 (in ChR2) is hydrogen-bonded to Cys128 to form a DC-gate that acts as a switch for the movement of ions [30]. Mutation of Cys128 to Thr (C128T) delays the closure of the ion channel gate and therefore remains conducting for a longer period [31]. This mutation has enhanced the property of ChR2 to be used as an optogenetic tool. Cys128 is also conserved in newly identified modular ChRs (Figure 2 and Table 1B).

The conservation of important amino acids reflects their functionality and could be engineered to enhance their properties. Thus, newly identified modular ChRs hold the potential to be used as optogenetic tools for controlling new biological pathways. 

Apart from the three modular ChRs, the genome database search also led to the identification of many modular sensory-type rhodopsins from different alga. A diverse set of domains fused with modular sensory-type rhodopsins were identified in a single ORF, which suggests multiple light-mediated cellular signaling pathways in these algae. Most of the identified rhodopsins are coupled with the two-component histidine kinase (HisK) and response regulator (RR) system. The first modular rhodopsin identified and characterized was Chlamyopsin5 (Cop5/HKR1) of *C. reinhardtii* [32].

### 3.3. Modular Sensory-Type Rhodopsins and Their Optogenetic Potential 

In the Cop5 modular organization, rhodopsin was coupled with HisK and RR domain along with Cyc, SMC_N, and SAM (Figure 1B). Experimental evidence suggests that Cop5 localizes in the eyespot of *C. reinhardtii*, with dichromic absorbance maxima in the UV range however, their native functional role is still not clear [32]. Followed by Cop5, many other rhodopsins with similar domain architecture were identified in *C. reinhardtii* and other algae as well. Cop6–8 expressions were further confirmed in *C. reinhardtii* and Cop8 was localized in cilia and eyespot in a light-dependent manner [7]. Similar homologs of the modular rhodopsins were identified in another closely related colonial green algae *Volvox carteri* and other algae (Figure 1B). Along with HisK and RR, other domains like Cyc, SMC_N, Tnp, and SAM were also coupled in some modular rhodopsins as shown in Figure 1B. Interestingly, GpRh5 and OtRh2 possess domains (RPT1 and BRD in GpRh5; TPR in OtRh2) at the N-terminus of rhodopsin and the two-component system at the C-terminus of rhodopsin (Figure 1C, Table 2A). Another group of modular rhodopsin lacks the two-component system but is coupled to a unique domain like SPRY, DUF, and MED15 (Figure 1D). AsRh4 is unique among this group in possessing Rav1 and WD40 at the N-terminus of rhodopsin (Figure 1D). We have summarized the modular sensory-type rhodopsins according to their domain architecture, cellular function, and possible optogenetic applications in Table 2A.

### 3.4. Light-Gated Ion Pump and Photo-Sensory Function Prediction Based on Conserved Residues of Rhodopsins

**Figure 3:**
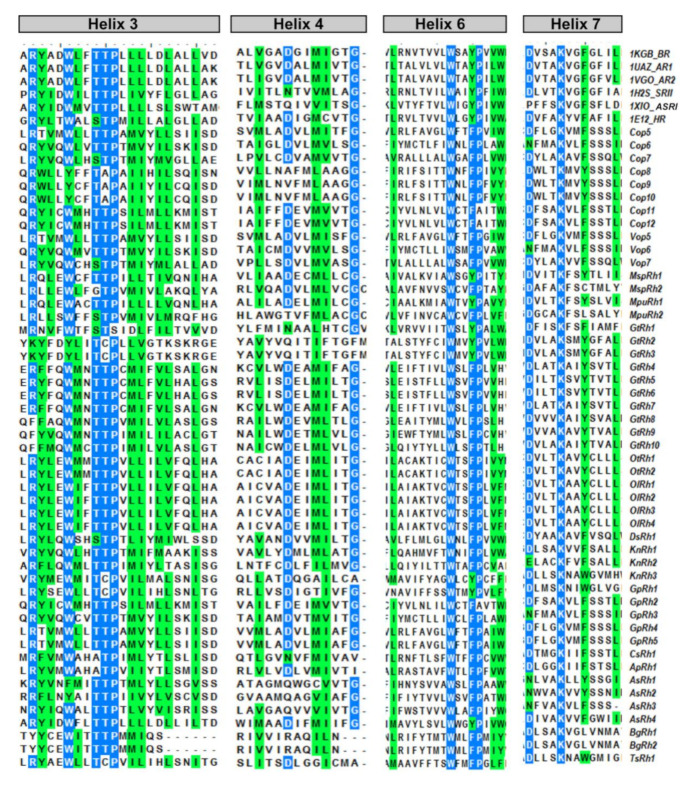
Comparison of light sensor domain of the modular rhodopsin among different algae: Most conserved third, fourth, sixth, and seventh helices of rhodopsin are depicted here. Numbering was adapted according to the protein of BR. 1KGB: Bacteriorhodopsin, 1UAZ: Archaerhodopsin-1, 1VGO: Archaerhodopsin-2, 1El2: Halorhodopsin, 1H2S: Sensory Rhodopsin II, 1XIO: Anabaena sensory rhodopsin.

Amino acids in the proximity of retinal are the key determinants in the activation and function of rhodopsins. The crystal structure of BR suggests that Asp85 is the proton acceptor from RSB during deprotonation. Thr89 is hydrogen-bonded to Asp85 (Figure 3 and Table 2B). Thr90 forms a part of the retinal binding pocket and the corresponding position in ChR2 (Cys128) forms the DC-gate regulating the movement of ions. Asp212 forms a part of counterion and thus, plays a role during the primary proton transfer event. Asp96 donates a proton to the RSB during reprotonation. Glu194 and 204 are the terminal amino acids responsible for the outward release of protons to the extracellular side. These positions were analyzed in the modular rhodopsins to assign their functionality. Out of 47 modular rhodopsins at position 85, 14 had conserved Asp/Glu while 17 had Gln (Figure 3 and Table 2B). Position 89 is well conserved with 43 out of 47 modular rhodopsins possessing Ser/Thr at this position (Figure 3 and Table 2B). Asp96 is only conserved in AsRh4 (Table 2B). Asp212 is well conserved among modular rhodopsins except 6 of them which possess Asn at this position (Figure 3 and Table 2B). Only 4 modular rhodopsins possess Asp at 194th position while 25 modular rhodopsins have Glu at 204th position (Figure 3 and Table 2B). These rhodopsins seem to be functional since the retinal binding lysine is conserved among all of them (Figure 3 and Table 2B). AsRh4 is the only modular rhodopsin with an amino acid conserved for proton pump. Other modular rhodopsins seem to form a new group with a different mechanism for activation and relay of signals. Despite lacking the proton acceptor Asp85, Cop5 was found to be active in UV-A and blue light (Figure 3 and Table 2B). Cop6/Vop6 behaves as a light inhibited guanylate cyclase in the presence of ATP when expressed in *Xenopus* oocyte [33] even though; it lacks Asp85, Asp96, and Asp212 (Figure 3 and Table 2B). The signal relay in Cop6/Vop6 proceeds through HisK and RR. OtRh1/Ot-HKR is a green absorbing modular rhodopsin controlling the circadian clock of *O. tauri*. The photophysical properties of OtRh1/Ot-HKR are affected by salt concentration indicating this rhodopsin might provide input for adaptation in the salt environment [34]. These examples suggest that the important amino acids are substituted but these rhodopsins are still functional. Unique domains coupled with rhodopsin might regulate specific function in cell/organism and hold potential to be used as optogenetic tools and therefore should be explored in detail.

### 3.5. Spectral Tuning of the New Microbial Rhodopsins

The amino acid residues surrounding the chromophore are primarily responsible for tuning the absorbance maxima of the holoprotein rhodopsin. The significant role of amino acids in spectral tuning was studied in the case of green and blue proteorhodopsins (GPR and BPR, respectively). The amino acid residue at the 105th position of the highly homologous green absorbing proteorhodopsin (GPR: AY210898) and blue absorbing proteorhodopsin (BPR: AY210919) have nonpolar leucine and polar glutamine residues, respectively. The substitution of either convert it into the other form and vice versa [35]. The four rhodopsins of halobacteria BR, HR, SRI, and SRII have the same bound chromophore but SRII shows a blue-shifted absorbance at 498 nm as compared to BR, HR, and SRI by 60 to 80 nm. Point mutations of all residues in the retinal pocket in archaeal SRII corresponding to BR did not shift the maxima of SRII to BR [36,37]. This suggests that spectral tuning is also regulated by other structural feature(s) of rhodopsins, probably by residues present at the flanking sides of the retinal binding pocket. The absorption spectrum of animal rhodopsin covers the entire visible range from UV-A to NIR. Absorbance maxima of MTRs are largely confined to the blue and green region of the spectra But, the recently characterized Cop5, modular rhodopsin coupled with HisK, RR, and Cyc suggest its tuning to UV-A and blue light (bi-stable switch). The chromophore isomerization and counterion distance were involved in spectral shift [32,38,39]. Based on the sequence analysis and comparison of residues corresponding to the 105th position (proteorhodopsin), the spectral shift (blue or green) of the modular rhodopsin has been analyzed and summarized in Table 3. This analysis suggests that newly identified modular rhodopsins are green tuned due to the presence of a nonpolar amino acid at the position corresponding to the 105th position (proteorhodopsin) except GtRh1 which possesses an acidic amino acid.

In addition to blue and green-shifted MTRs, the red-shifted MTRs [40,41,42,43,44] have also been reported with advantages over the former such as better light penetration, less scattering by biological material, and reduced phototoxicity. Many factors were found to be responsible for the red-shift such as the substitution of amino acid residues near the retinal binding pocket, the protonation state of the counter-ion for RSB, and the distribution of polar amino acids [43]. These factors constitute a challenge to predict a model for the newly identified modular rhodopsins for their red-shifted spectral tuning. The blue and green tuned rhodopsins can be engineered to obtain the red-shifted molecules thereby making them suitable for optogenetic applications. Spectral response in long-wavelength (~590 to 630 nm) was achieved by the engineering of VChR1 named as red activated ChR, ReaChR (VChR1 with N terminus of CHEF/CHIEF, transmembrane helix F of VChR2 and point mutation Lue171Ile [45]). Point mutations (P219T/S254A) in the sodium ion pump (KR2) led to a red-shift of 40 nm without affecting its ion pumping activity [46]. The addition of the retinal analog 3-methylamino-16-nor-1,2,3,4-didehydroretinal (MMAR) led to a red-shift in the archaerhodopsin-3 absorption spectra [47]. The modular ChR would be expected not only to change the membrane potential but also to modulate the specific signaling pathways linked with coupled domains (Figure S3A,B). MED15 expression controls the malignancies and progression of the tumor, and suppression of MED15 leads to cancer progression. On a speculative note, the MED15 domain of ChR might be involved in reversing the light-mediated cellular toxicity in these organisms that depend on light for their photo-behavioral responses. Hence MED15 coupled ChRs might be an excellent tool for optogenetic stimulation of the cells. Moreover, other additional tools like genetic engineering, exogenous supply of other compounds like MMAR, etc., mentioned above to red-shift the spectral tuning might be the added benefits of their optogenetic applications.

### 3.6. Evolutionary Pattern of the Modular Microbial Rhodopsins

MTRs provide a smart alternative pathway of ATP production, other than photosynthesis, in archaea and help in the survival of the organism in harsh conditions. Many reports have been published regarding the evolutionary pattern of MTRs [48,49] but the descent of modular rhodopsins is not yet known. As this is the first report of modular rhodopsin from diverse organisms; it is noteworthy to analyze the evolutionary pattern of these rhodopsins from different taxa of life. 

**Figure 4:**
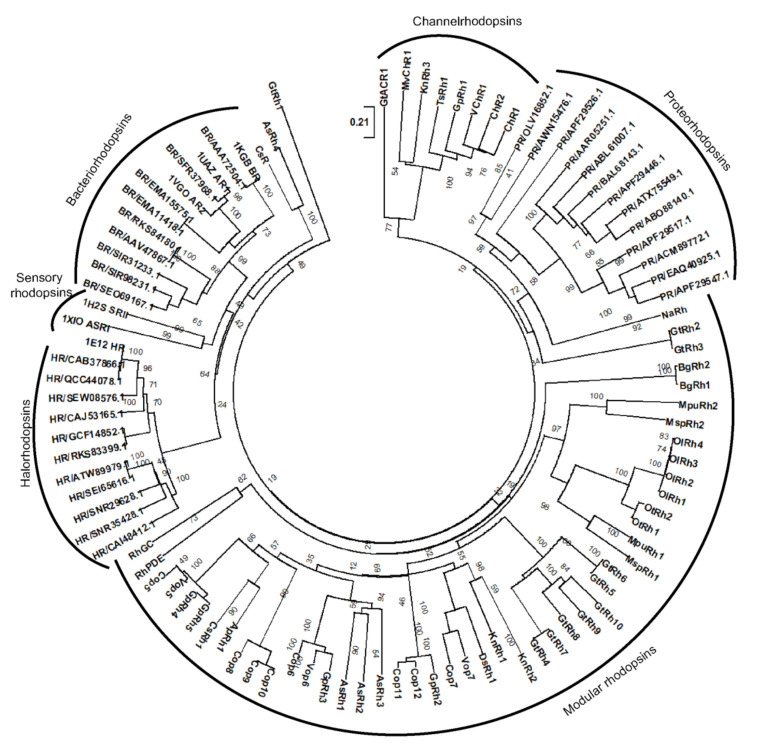
Sequence relatedness of the microbial type modular rhodopsin: Rhodopsin domain phyletic topology shows clustering of typical MTR and extended C-terminus rhodopsins in a separate clade. Modular rhodopsins formed a different clade. KnRh3, GpRh1 and TsRh1 grouped with ChRs. AsRh4 with Rav1 domain is the only modular rhodopsin grouped with proton pumping algal rhodopsin CsR (Rhodopsin from *Coccomyxa subllipsodea*). GtRh1 was unique and separated from all lying between BR and HR.

FimV, UL36, and MED15 coupled Channelrhodopsins (KnRh3, GpRh1, and TsRh1) were grouped with ChR and VChR (Figure 4) while rhodopsins from proteobacterium, proton pumping BR, chloride pumping HR and SR clustered in separate clades (Figure 4). Interestingly, AsRh4 preceded by Rav1 and WD40 domain at N-terminus was the only modular rhodopsin grouped with algal proton pump CsR from *Chlorella subellipsoedea*. Sequence alignment also confirmed the presence of important residues required for pump activity in AsRh4 (Figure 2 and Figure 3, see text). Surprisingly, modular rhodopsins clustered together independently of SRs. A closer analysis of the branching pattern showed that the ChRs grouped with the modular rhodopsins more closely than the proton pumping algal rhodopsins revealing their unique functional properties. Among the ChRs, the best-characterized one is the light-driven ion channel. The spectroscopically characterized modular rhodopsin domain, Cop5, is a UV and blue light-absorbing rhodopsin [32,38,39]. Cop6 expressed in *Xenopus laevis* behaves as a light-inhibited guanylate cyclase in the presence of ATP [33]. The photophysical properties of histidine kinase rhodopsin Ot-HKR (referred here as OtRh1) from *O. tauri* are affected by salt concentration indicating that this rhodopsin might be involved during adaptation in the salt environment [34]. OtHKR/OtRh1 speculated to regulate the circadian clock genes TOC1 and CCA shows a higher expression during dusk [34]. The characterization of additional multidomain rhodopsins is tempting because it may unearth entirely new classes of rhodopsins not known yet. At the same time, it is limiting because of long transcript and high molecular weight protein, poor heterologous expression of the full length and transmembrane domain, and the lack of established functional assays.

### 3.7. Cyclase Domain is a Canonical Secondary Messenger of Modular Sensory Type Rhodopsin

**Figure 5:**
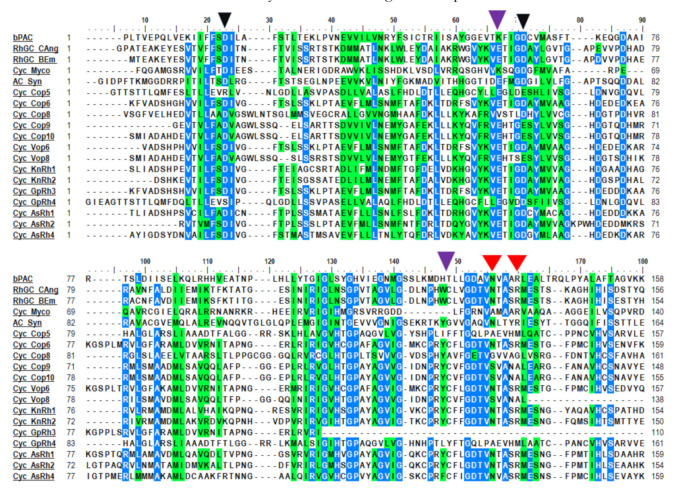
Multiple sequence alignment of the cyclase domain of modular rhodopsins: Cyclase domains of modular rhodopsins were aligned with canonical cyclase proteins. Black arrowhead depicts metal-binding residue, purple arrowhead shows substrate-binding residue and the red arrowhead shows transition state stabilizing the residues of the cyclases.

Cyclases are a lyase class of enzymes that catalyze the formation of cyclic nucleotides. Cyclic nucleotide monophosphate (cNMP) serves as a signaling molecule in many prokaryotes and eukaryotes. Based on the substrate specificity, there are two classes of cyclases—adenylyl cyclase (AC) and guanylyl cyclase (GC). Multidomain cyclases are generally composed of a receptor domain at the N-terminus and a cyclase domain at the C-terminus with a kinase homology domain in the center. A similar architecture is found in modular rhodopsin coupled cyclases. Sequence analysis suggests that most cyclase domains have a conserved amino acid residue to perform the enzymatic activity. Cop5 and Vop5 lack the conserved aspartate involved in metal binding (Figure 5). Substrate binding and transition state stabilizing residues are also absent in Cop5 and Vop5 (Figure 5). This points towards an inactive cyclase, which was also confirmed by the SMART domain analysis program. Cyclases generally function in the dimer state with the active sites being located at the dimer interface. The activity requires a divalent cation, either Mg^2+^ or Mn^2+^. The conserved motifs, especially the transition state stabilizing residues of the cyclase are also missing, which suggests that other transition state stabilizing molecules might be involved in signaling (Figure 5). Both monomers work in tandem to carry out cyclase activity where one determines substrate specificity whereas metal-binding sites are provided by the other monomer. The inactive cyclase might form regulation and another functionally active monomeric partner may complement the activity of the cyclase.

In *C. reinhardtii*, cAMP induces the rapid mobilization of membrane adhesion receptor protein from the cell membrane to the ciliary membrane in gametes [27] which leads to the adhesion and fusion of gametes to form the zygote and hence, promotes its sexual life cycle [50]. In the phototaxis mutant strain of *C. reinhardtii*, cyclase activity biases the photo-behavioral response and carotenoid biosynthesis [51]. The modular rhodopsins in conjunction with the two-component system and cyclase might be performing diverse light-regulated physiological functions in the green alga. Sequence analysis suggests degenerate cyclase in Cop5 and Vop5. Apart from the ciliary signaling, cilia beating pattern, phototaxis, and communication with eyespot, some modular rhodopsin(s) must have a diverse physiological role and be localized to a different place than the eyespot [7]. These above-mentioned hypotheses get strong support from the fact that homologous modular rhodopsins are also present in the non-flagellated, eyespot devoid, unicellular green algae *Ostreococcus lucimarinus*, symbiotic algae, and in colonial algae *Volvox carteri*. The rhodopsin coupled guanylyl cyclase from the fungus *Blastocladiella emersonii* was required for the phototactic behavior of the zoospore and had shown *in vitro* functional activity as well [52]. Rho-GC from other fungi showed promising results in modulating light-dependent cGMP levels in *Xenopus* oocytes, hippocampal neural cells, and Chinese hamster ovary cells [12,13]. It will be interesting to investigate the functional modulation of cAMP/cGMP in the cell by the modular algal rhodopsins as well.

### 3.8. Optogenetic Potential of the Novel Modular Rhodopsins

Among a variety of effector domains coupled with the ChRs, we selected the FimV, MED15, and UL36 domains of functional importance, which have not yet been characterized in the algal system. We subjected these domains to protein-protein interaction network analysis and identified their potential partners and associated pathways. The protein-protein interaction analysis for the FimV domain revealed its association in regulating bacterial pathogenesis machinery (Figure S1A). In the opportunistic pathogen *Pseudomonas aeruginosa*, FimV is an inner membrane hub protein that controls the type IV pilus (T4P)-mediated twitching motility by regulating the intracellular cAMP level via activation of the adenylate cyclase CyaB [53,54]. Factors like pili, flagella, toxin, etc., that determine the virulence/pathogenicity of microbes are controlled by cAMP, an allosteric activator of the virulence factor regulator, Vfr [55]. However, FimV and the Chp system (PilG, PilJ, PilN, and PilF) also regulate the twitching motility in a cAMP-independent manner in *P. aeruginosa*, where PilG may regulate the directional movement, while FimV functions to localize both structural and regulatory elements to the cell poles for an optimal function [54]. Therefore, based on the protein network analysis, we propose that the ChR coupled FimV domain could be used for the optogenetic control of cAMP-dependent as well as independent pathways to regulate twitching motility that may elucidate the molecular signaling pathways of pathogen invasion (Figure S3A).

MED15 (co-activator) plays a crucial role in the transcriptional regulation of RNA polymerase II-dependent genes [56]. The protein-protein interaction analysis of the MED15 domain showed that it interacted with other mediator complex subunits (Figure S1B). MED15 was identified as the regulator of mammalian sterol regulatory element-binding protein 1α (SREBP1α) which controls the genes involved in cellular cholesterol and lipid homeostasis [57]. MED15 possesses a conserve “KIX fold” and is responsible for binding to SREBP1α. This fold is also conserved in the *Caenorhabditis elegans* orthologue, MDT15, and yeast orthologue GAL11p [57,58]. It has also been reported that the deregulation of the MED15 expression promotes human malignancies and inactivation of MED15 may inhibit the progression of several types of cancers [56,59]. Several studies found that MED15 is an important prognostic biomarker for patients with various types of carcinomas [56,59]. In breast cancer and few epithelial cancers, the inactivation of MED15 inhibits the aberrant transforming growth factor β (TGFβ) -induced epithelial-mesen chymal transition (EMT), as it acts as a crucial cofactor for TGFβ signaling [60]. The localized tumor-specific expression of ChR coupled MED15 could be used to target tumor cell signaling and eventually induce the tumor for autophagy or growth arrest in conjunction with other engineered proteins, in a light-dependent manner. Figure S3B represents a probable model for ChR coupled MED15 mediated optogenetic regulation of promoter initiation complex (PIC) assembly (a crucial step in transcriptional regulation), the dysregulation of which leads to oncogenic proliferation. 

The UL36 domain, associated with modular ChR, GpRh1 from *G. pectorale* is a tegument viral protein found in herpes simplex virus 1 (HSV-1) and its homologs are well distributed across the members of *Herpes viridae* [61]. UL36 protein is an ubiquitin-specific protease [62] which is also evident from our protein-protein interaction analysis of UL36 protein (Figure S2A). Most of the interacting partners like Ubiquitin, 26S proteasome regulatory subunit S5A, proteasome regulatory particle subunit (RpnC), and DSS1/SEM1 family protein belongs to the ubiquitin-dependent proteolysis machinery [63,64,65]. Proteasome subunit S5a (the human homolog of Rpn10) functions in conjunction with hHR23a/b (the two human homologs of Rad23) to recruit ubiquitylated substrates to the proteasome for their degradation [66]. In humans, DSS1/SEM1 is related to a tumor suppressor protein (BRCA2), which has a crucial role in the recombinational DNA repair in association with RAD51 [67,68]. UL36 deubiquitinating activity has a role in inhibiting the interferon-mediated immune defense upon viral invasion in the host [62]. Interestingly, the UL36 domain coupled to GpRh1 showed similarity to the C-terminal segment of HSV-1 UL36 protein (Figure S2B). Böttcher et al. (2005), in a mutation analysis with UL36 homologs from Pseudorabies virus, constructed several truncations and showed that the extreme C-terminus of UL36 having proline/alanine-rich region, is crucial for viral replication [69]. In the proposed model ChR coupled UL36 (C-terminal segment), could be used to regulate capsid assembly, retrograde transport of capsid, entry of viral DNA into the nucleus of the infected host cell as well as nuclear egress (Prototypic Vesicular Nucleo cytoplasmic Transport) in a light-dependent manner (Figure S3C). Based on protein-protein interaction analysis, it may be assumed that ChR coupled effector domains can be utilized as the next generation optogenetic tools, which might help in controlling processes ranging from lipid metabolism, ubiquitin-mediating proteolysis, and pathogenesis to carcinogenesis. Apart from the natural variant, the modular rhodopsins could also be genetically engineered for enhanced kinetics, better spectral tuning (red-shifted spectral compatibility), and modulation to precisely control diverse cellular physiological responses. Hence, the computational analysis of the identified rhodopsins provides an insight into their functionality and further experimental characterization would expand the existing optogenetic toolkit.

## 4. Conclusions

In this study, we have reported various rhodopsins with diverse effector domains. Based on multiple protein sequence alignments and phylogenetic analysis, these modular rhodopsins can be categorized as ChRs, ion pumping (AsRh4), or sensory-type rhodopsins. Owing to the diverse functions offered by the encoded effector domains of these modular rhodopsins hold great potential to expand the optogenetic toolkit. We have also proposed the working models of the modular channelrhodopsins (i.e., ChR-FimV, ChR-MED15, and ChR-UL36), in regulating processes ranging from bacterial pathogenesis, transcription to viral replication and light-gated proteasomal regulation, respectively. The established methods for expression and delivery system could be systematically utilized to design further experiments to study the modular rhodopsin mediated optogenetic modulation of crucial processes across the biological systems. These naturally occurring light-sensitive rhodopsin modules could be recruited in the biological systems and activated relevant approaches such as forced conformational change, heterodimerization, etc. These conformational changes could bring desired changes in cellular signaling like gene expression, protein translocation, and receptor signaling pathways. Empirical optimization, targeted engineering, and directed evolution of the modular rhodopsin(s) would enable us to refine light-sensing mechanisms (e.g., development of red/near infra-red shifted spectral tuning of the rhodopsin) and engineering of the coupled effector domain(s) for extensive applications avenues in optogenetics.

## Figures and Tables

**Table:** (**A**)

Modular Domain	Channelrhodopsin	Functional Role and Optogenetic Potential
FimV (Peptidoglycan binding protein)	KnRh3	In bacteria: Controls bacterial pathogenesis by indirectly activating adenylyl cyclase and hence cAMP level.
MED15 (Subunit of mediator complex)	TsRh1	In mammals: Regulates cholesterol and lipid homeostasis. Promotes cancerous growth and is used as a biomarker for malignancies.
UL36 (Large tegument protein)	GpRh1	Regulates viral entry to the cells.

**Table:** (**B**)

Function of the Residue	Proton Acceptor	Proton Donor	DC Gate	Stabilizes Proton Acceptor	Retinal Attachment
No. corresponds to ChR2	253	156	128	120	257
ChR2	D_253_	D_156_	C_128_	R_120_	K_257_
KnRh3	D_250_	D_154_	C_126_	R_118_	K_254_
TsRh1	D_236_	D_139_	C_111_	R_103_	K_240_
GpRh1	D_213_	D_116_	C_88_	R_80_	K_217_

**Table:** (**A**)

Modular Domain	Modular Rhodopsins	Cellular Role and Optogenetic Potential
HisK	DsRh1, GtRh4-10, Cop5-12, Vop5-8, AsRh1-3, GpRh2-5, KnRh1 & 2, OtRh1&2, OlRh1-4, MpuRh1&2, Msp1&2, CsRh1, ApRh1, BgRh1&2	Part of two-component signaling; regulates gene expression
HisK-RR (Histidine kinase-response regulator) Two-component signaling system	GtRh4-8, Cop5-12, Vop5-8, AsRh1-3, GpRh2-5, KnRh1 & 2, OtRh1&2, OlRh1-4, MpuRh1&2, Msp1&2, CsRh1, ApRh1, BgRh1&2	Regulates gene expression and various other cell processes via output domain like helix-turn-helix (HTH), RNA, enzyme, or ligand-binding domain.
Cyc (Cyclase)	Cop5, 6, 8, 9 &10, Vop6&8, AsRh1-3, GpRh3&4, KnRh1 & 2	Regulates the level of secondary messengers: cAMP and cGMP.
SMC_N (Structural Maintenance of chromosome _N terminal)	Cop5, Vop5, GpRh4	Stabilizes the chromosome, helps in its proper segregation during cell division and DNA repair.
Tnp (Transposase)	Cop9 & 10	Recognizes the transposable elements in DNA and catalyzes their movement to another DNA.
SAM (Sterile alpha motif)	Cop5-8, Vop5, KnRh1 & 2	Mediate protein-protein interactions, RNA and lipid binding; regulates transcription factor
ICP4 (Infected-cell polypeptide 4)	KnRh1	Major transcription factor of herpes simplex virus type1 (HSV-1)
Mn104 (Microneme/rhoptry)	KnRh1	Helps in invading host cell by apicomplexan parasites; N-terminal region proposed to serve as a signal peptide for ER
MotB (Flagellar motor protein)	KnRh2	MotB acts as a stator in the proton pump.
RPT1 (Regulatory Particle Triple ATPase)	GpRh5	Forms a part of 26S proteasomal complex
BRD (Bromodomain)	GpRh5	Modulate gene expression by associating with acetylated lysine on histone
TPR (Tetracopeptide repeat)	OtRh2	Regulates virulence in bacteria; translocation of receptors to their respective organelles in different systems
SPRY [Spore lysis A (Spl A) in *Dictyostelium discoideum* and mammalian Ryanodine receptor (RYR)]	GtRh1	Substrate binding for ubiquitination in ubiquitin ligase family proteins; involved in the various immune response
DUF (Domain of unknown function)	GtRh1	Mediate protein-protein interaction and transcription repression; ATP dependent protein kinase; enzymatic part of dicer; virulence and pathogenesis.
Rav1 (Regulator of V-ATPase of vacuole membrane protein 1)	AsRh4	Regulates the assembly of V-ATPase (ATP powered H+ pump in vacuole forming organelles)
WD40	AsRh4	Mediate protein–protein interaction

**Table:** (**B**)

Function of the Residue	Ion Pumping					Proton-Release to Outside		Retinal Attachment
No. corresponds to BR	85	89	90	96	212	194	204	216
BR	D	T	T	D	D	E	E	K
HR	T_90_	S_94_	T_95_	A_101_	D_217_	E_198_	T_209_	K_221_
KR2 (Na+)	N_112_	D_116_	V_117_	Q_123_	D_251_	L_227_	R_243_	K_255_
ASR1	D_75_	T_79_	T_80_	S_86_	P_206_	S_188_	D_198_	K_210_
SR2	D_75_	T_79_	T_80_	F_86_	D_201_	L_188_	D_193_	K_205_
RhoGC	E_254_	T_258_	C_259_	L_265_	D_380_	S_364_	A_372_	K_384_
RhoPDE	E_164_	T_168_	C_167_	W_175_	D_292_	Q_276_	G_284_	K_296_
AsRh4	D_2593_	T_2597_	T_2598_	D_2604_	D_2718_	G_2701_	E_2710_	K_2722_
GtRh1	F_152_	S_156_	T_157_	I_163_	D_297_	G_280_	K_289_	K_301_
GtRh2/3	D_95_	T_99_	C_100_	T_106_	D_248_	T_232_	E_240_	K_252_
Cop5	M_113_	T_117_	T_118_	L_124_	D_239_	M_223_	E_231_	K_243_
Cop6	Q_170_	T_174_	T_175_	I_181_	N_294_	V_279_	-	K_298_
Cop7	Q_161_	S_165_	T_166_	M_172_	D_287_	W_271_	E_279_	K_291_
Cop8	L_67_	T_71_	A_72_	I_78_	D_194_	D_178_	S_186_	K_198_
Cop9-10	L_141_	T_145_	A_146_	I_152_	D_268_	D_252_	S_260_	K_272_
Cop11	C_95_	T_99_	T_100_	L_106_	D_279_	L_263_	E_271_	K_283_
Cop12	C_95_	T_99_	T_100_	L_106_	D_221_	L_205_	E_213_	K_225_
Vop5	M_157_	T_161_	T_162_	L_168_	D_283_	L_267_	E_275_	K_287_
Vop6	Q_153_	T_157_	T_158_	I_164_	N_278_	L_263_	-	K_282_
Vop7	Q_147_	S_151_	T_152_	M_158_	D_272_	W_256_	E_264_	K_276_
MspRh1	E_140_	T_144_	T_145_	I_151_	D_284_	F_268_	Q_276_	K_288_
MspRh2	E_142_	G_146_	T_147_	L_153_	D_299_	S_283_	L_291_	K_303_
MpuRh1	E_140_	T_144_	T_145_	I_151_	D_300_	F_284_	Q_292_	K_304_
MpuRh2	S_151_	S_155_	T_156_	L_162_	D_328_	A_312_	A_320_	K_332_
GtRh4	Q_92_	T_96_	T_97_	V_103_	D_225_	S_209_	Y_217_	K_229_
GtRh5	Q_222_	T_226_	T_227_	V_233_	D_355_	G_339_	Y_347_	K_359_
GtRh6	Q_234_	T_238_	T_239_	V_245_	D_367_	G_351_	Y_359_	K_371_
GtRh7	Q_116_	T_120_	T_121_	V_127_	D_249_	S_233_	Y_241_	K_253_
GtRh8	Q_226_	T_230_	T_231_	V_237_	D_359_	L_343_	Y_351_	K_363_
GtRh9	Q_229_	T_233_	T_234_	I_240_	D_362_	L_346_	Y_354_	K_366_
GtRh10	Q_192_	T_196_	T_197_	V_203_	D_325_	L_309_	F_317_	K_329_
BgRh1/2	E_173_	T_177_	T_178_	S_184_	D_302_	L_286_	E_294_	K_306_
OtRh1	E_181_	T_185_	T_186_	L_192_	D_314_	M_298_	E_306_	K_318_
OtRh2	E_476_	T_480_	T_481_	L_487_	D_609_	M_593_	E_601_	K_613_
OlRh1	E_204_	T_208_	T_209_	L_215_	D_337_	L_321_	E_329_	K_341_
OlRh2	E_260_	T_264_	T_265_	L_271_	D_393_	L_377_	E_385_	K_397_
OlRh3	E_188_	T_192_	T_193_	L_199_	D_321_	L_305_	E_313_	K_325_
OlRh4	E_115_	T_119_	T_120_	L_126_	D_248_	L_232_	E_240_	K_252_
DsRh1	Q_140_	S_144_	T_145_	M_151_	D_268_	L_252_	E_260_	K_272_
GpRh2	C_91_	T_95_	T_96_	L_102_	D_217_	L_201_	E_209_	K_221_
GpRh3	Q_85_	T_89_	T_90_	I_96_	N_209_	A_194_	-	K_213_
GpRh4	M_67_	T_71_	T_72_	L_78_	D_193_	L_177_	E_185_	K_197_
GpRh5	Q_1412_	S_1416_	T_1417_	M_1423_	D_1537_	L_1521_	E_1529_	K_1541_
CsRh1	M_144_	A_148_	T_149_	T_155_	D_269_	L_253_	E_261_	K_273_
ApRh1	M_67_	A_71_	T_72_	T_78_	D_192_	A_176_	E_184_	K_196_
AsRh1	N_122_	T_126_	T_127_	L_133_	N_248_	L_232_	T_240_	K_252_
AsRh2	N_123_	T_127_	T_128_	L_134_	N_249_	L_233_	S_241_	K_253_
AsRh3	Q_78_	T_82_	T_83_	V_89_	N_203_	L_187_	C_195_	K_207_
KnRh1	Q_166_	T_170_	T_171_	M_177_	D_292_	L_276_	E_284_	K_296_
KnRh2	Q_95_	T_99_	T_100_	L_106_	E_221_	T_205_	E_213_	K_225_

**Table 3:** Comparative Analysis of the Amino Acid Residues Determining Spectral Tuning of the Rhodopsin.

Rhodopsin	105th Position/ Corresponding Amino Acid	Polar/Non-Polar aa	Green/Blue Shifted
Green PR	Leucine	Non-Polar	Green
Blue PR	Glutamine	Polar	Blue
KnRh3, TsRh1 and GpRh3	Isoleucine	Non-polar	Green
Cop8-12, GpRh2, ApRh1, AsRh2	Isoleucine	Non-polar	Green
MspRh1, MpuRh1, AsRh3-4, OtRh1-2, OlRh1-4, DsRh1, GtRh2,3	Leucine	Non-polar	Green
Cop5-7, Vop5-7, GpRh3-5, GtRh4-10, AsRh1, MspRh2, MpuRh2, CsRh1, BgRh1-2, KnRh1-2	Methionine	Non-polar	Green
GtRh1	Aspartate	Acidic	unknown

## Supplementary Materials

The following are available online at https://www.mdpi.com/2075-1729/10/11/259/s1, Figure S1: Protein-Protein interaction network showing interacting partners of (A) FimV and (B) mediator complex subunit 15 (MED15) domains of modular ChRs. Figure S2: Interactome and sequence alignment of UL36 domain of modular ChR (GpRh1). Figure S3: Schematics representing optogenetic potentials of the modular ChRs. Table S1: Sequence identity of modular rhodopsin used in the analysis and their protein sequences.

## Abbreviations

Cop-Chlamyopsin	rhodopsin from *Chlamydomonas reinhardtii*
Vop-Volvoxopsin	rhodopsin from *Volvox carteri*
GpRh 1−5	rhodopsin from *Gonium pectorale*
AsRh1−4	*Asterochloris sp.*
KnRh1−3	*Klebsormidium nitens*
OtRh1−2	*Ostreococcus tauri*
MpuRh1&2	*Micromonas pusilla*
MspRh1&2	*Micromonas species*
OlRh1−4	*Ostreococcus lucimarinus*
CsRh1	*Chlorella sorokiniana*
ApRh1	*Auxenochlorella protothecoides*
BgRh1&2	*Bigelowiella natans*
GtRh1−10	*Guillardia theta*
DsRh1	*Dunaliella salina*
TsRh1	*Tetraselmis subcordiformis*
