# Supplementary material for: Novel Modular Rhodopsins from Green Algae Hold Great Potential for Cellular Optogenetic Modulation Across the Biological Model Systems
Source: Life (Basel). 2020 Oct 28;10(11):259. doi: 10.3390/life10110259 (PMC7693036; doi:10.3390/life10110259)
Supplement: Supplementary file 1 [file life-10-00259-s001.pdf]

life

Novel Modular Rhodopsins from Green Algae Holds a Great Potential for Cellular Optogenetic Modulation Across the Biological Model Systems

Mayanka Awasthi [1,#], Kumari Sushmita [2,#], Manish Singh Kaushik [2] and Peeyush Ranjan [1,*] and Suneel Kateriya [2,*]

1 Department of Cell Biology and Molecular Genetics, University of Maryland, College Park, USA.

2 Laboratory of Optobiology, School of Biotechnology, Jawaharlal Nehru University, New Delhi, India.

# Equally contributed
* Corresponding Authors: skateriya@jnu.ac.in (S.K.), rpeeyush@umd.edu (P.R.)

Supplementary Figures

Supplementary Figure 1:

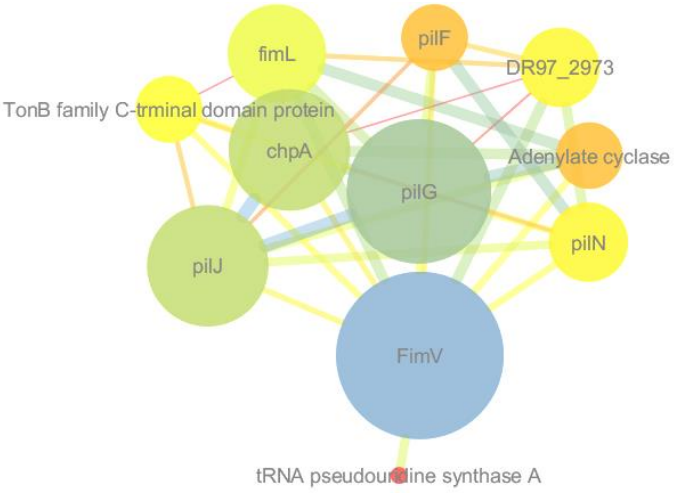

A

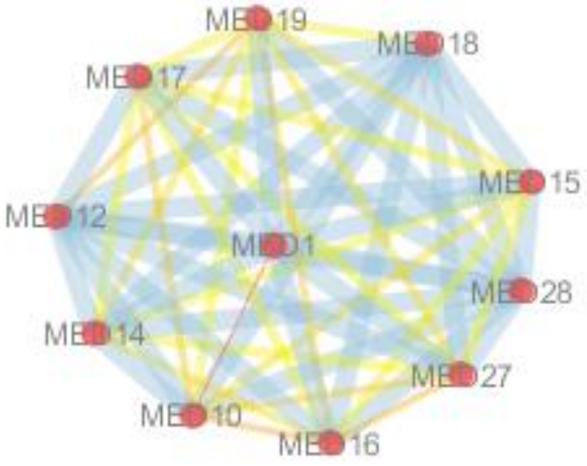

B

Figure S1. Protein-Protein interaction network showing interacting partners of (**A**) FimV and (**B**) mediator complex subunit 15 (MED15) domains of modular ChRs. Protein-protein interaction was performed using String version 11 (https://string-db.org/) and further modified by CytoScape 3.7.2 using node degree analysis. Size of the nodes represent number of interactions for each node and color scale ranging from red-orange-yellow-green to blue represent a score (confidence level) from low (< 0.4) to high (> 0.7) values.

Supplementary Figure 2:

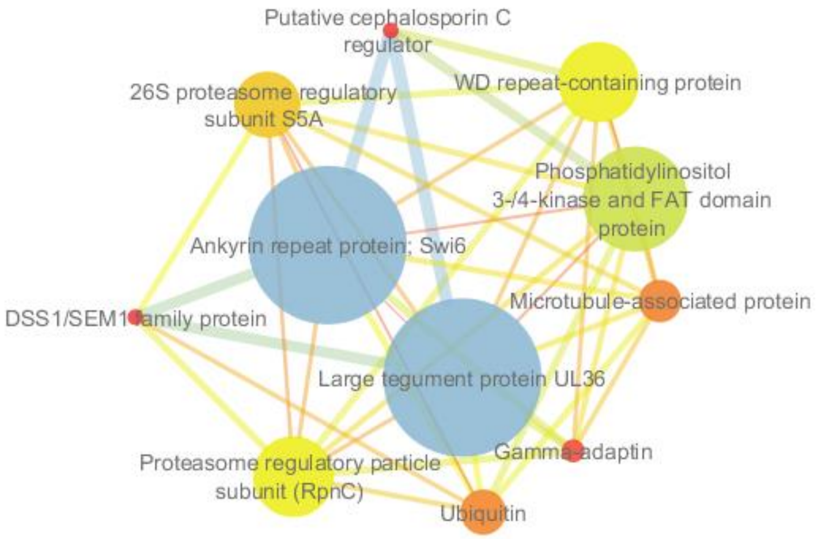

A

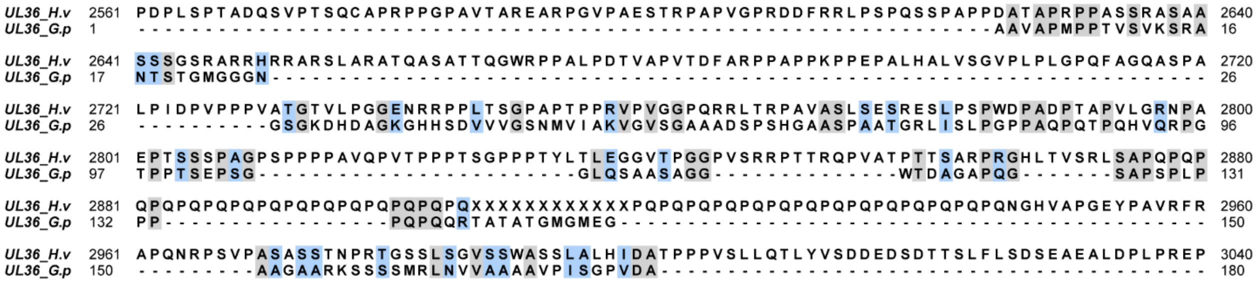

B

Figure S2. (**A**) Interactome of UL36 domain of modular ChR (GpRh1). Protein network was constructed with String version 11 (https://string-db.org/) and further subjected to node degree analysis using CytoScape 3.7.2. Size of the nodes represents number of interactions for each node. Color scale ranging from red-orange-yellow-green to blue represent confidence level from low (<0.4) to high (> 0.7) values. (**B**) The sequence alignment of UL36 domain of modular ChR (GpRh1) from *G. pectorale* with the C-terminal segment of UL36 (Accession Number: AWW11697.1) of human alpha herpes Virus-1. The sequence of UL36 domain of GpRh1 aligned well with that of C-terminal segment of HSV-1 UL36 protein having proline/alanine rich region which is crucial for viral replication. Identical amino acids are shown in blue and similar amino acid residues are shown in grey.

Supplementary Figure 3:

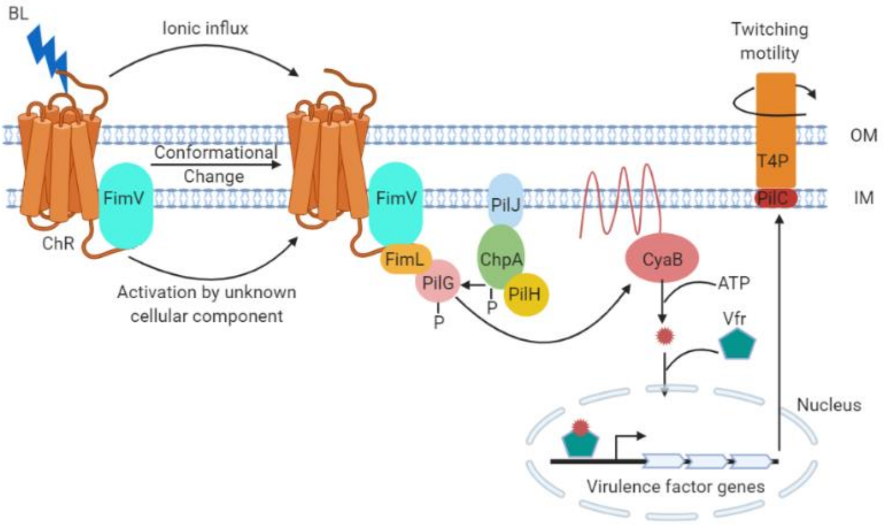

A

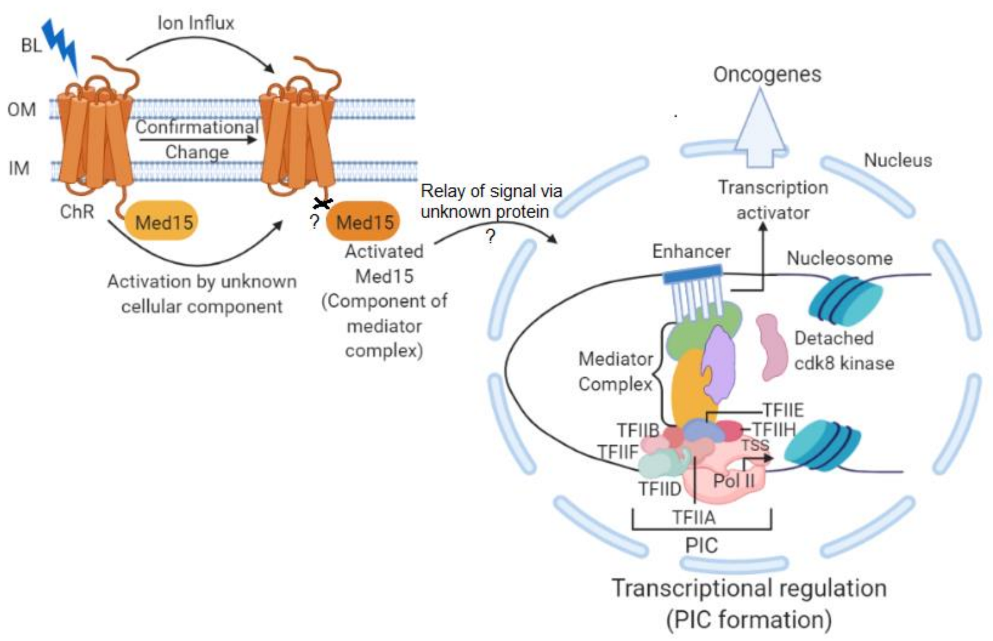

B

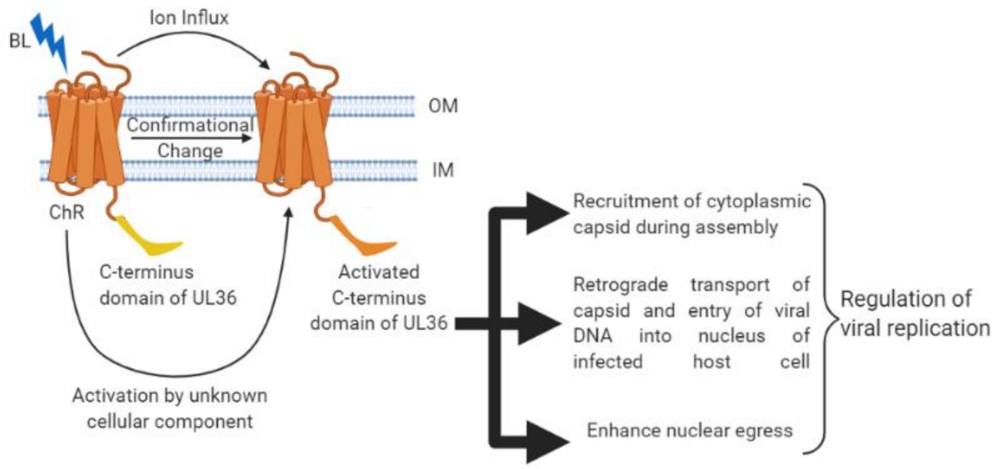

C

Figure S3. Schematics representing optogenetic potentials of the modular ChRs. (**A**) Light driven ChR coupled FimV (KnRh3) mediate regulation of machinery controlling twitching motility and virulence. (**B**) Optogenetic regulation of assembly of PIC by ChR coupled MED15 (TsRh1). (**C**) Light-gated control of viral replication in infected host cells by ChR coupled UL36 (GpRh1; C-terminal).BL: blue light, ChR: channelrhodopsin, cAMP: cyclic 3′, 5′-adenosine monophosphate, CyaB: adenylate cyclase, ATP: adenosine triphosphate, T4P: Type IV pilus, Vfr: virulence factor regulator, OM: outer membrane; IM: inner membrane, Pol II: RNA polymerase II, PIC: Promoter initiation complex, TFIIA: transcriptional factor II A, TFIIB: transcriptional factor II B, TFIID: transcriptional factor II D, TFIIE: transcriptional

factor II E, TFIIF: transcriptional factor II F, TFIIH: transcriptional factor II H, CTD: C-terminal domain, cdk8: cyclin dependent kinase 8, TSS: transcriptional start site.

Table S1. Sequence identity of modular rhodopsin used in the analysis.

S.no	Seq. name	Sequence identifier	Organism	Genome database link
1	>Cop5	AAQ16277.3	*Chlamydomonas reinhardtii*	https://www.ncbi.nlm.nih.gov/protein [1] [2]
2	>Cop6	Cre11.g467678.t1.1	*Chlamydomonas reinhardtii*	https://phytozome.jgi.doe.gov/pz/portal.html#!info?alias=Org_Creinhardtii [3]
3	>Cop7	Cre01.g038050.t1.1	*Chlamydomonas reinhardtii*	https://phytozome.jgi.doe.gov/pz/portal.html#!info?alias=Org_Creinhardtii [3]
4	>Cop8	Cre07.g329900.t1.1	*Chlamydomonas reinhardtii*	https://phytozome.jgi.doe.gov/pz/portal.html#!info?alias=Org_Creinhardtii [3]
5	>Cop9	Cre15.g643503.t1.1	*Chlamydomonas reinhardtii*	https://phytozome.jgi.doe.gov/pz/portal.html#!info?alias=Org_Creinhardtii [3]
6	>Cop10	Cre15.g643503.t2.1	*Chlamydomonas reinhardtii*	https://phytozome.jgi.doe.gov/pz/portal.html#!info?alias=Org_Creinhardtii [3]
7	>Cop11	Cre17.g733150.t1.1	*Chlamydomonas reinhardtii*	https://phytozome.jgi.doe.gov/pz/portal.html#!info?alias=Org_Creinhardtii [3]
8	>Cop12	Cre17.g733150.t2.1	*Chlamydomonas reinhardtii*	https://phytozome.jgi.doe.gov/pz/portal.html#!info?alias=Org_Creinhardtii [3]
9	>Vop5	Vocar.0044s0018.1	*Volvox carteri*	https://phytozome.jgi.doe.gov/pz/portal.html#!info?alias=Org_Vcarteri [4]
10	>Vop6	Vocar.0009s0380.1	*Volvox carteri*	https://phytozome.jgi.doe.gov/pz/portal.html#!info?alias=Org_Vcarter [4]
11	>Vop7	Vocar.0001s0831.1	*Volvox carteri*	https://phytozome.jgi.doe.gov/pz/portal.html#!info?alias=Org_Vcarter [4]
12	>Vop8	Vocar.0069s0008.1	*Volvox carteri*	https://phytozome.jgi.doe.gov/pz/portal.html#!info?alias=Org_Vcarter [4]
13	>MspRh1	62803	*Micromonas species*	https://phytozome.jgi.doe.gov/pz/portal.html#!info?alias=Org_MspRCC299 [5]
14	>MspRh2	61324	*Micromonas species*	https://phytozome.jgi.doe.gov/pz/portal.html#!info?alias=Org_MspRCC299 [5]
15	>MpuRh1	70932	*Micromonas pusilla*	https://phytozome.jgi.doe.gov/pz/portal.html#!info?alias=Org_MpusillaCCMP1545 [5]
16	>MpuRh2	40573	*Micromonas pusilla*	https://phytozome.jgi.doe.gov/pz/portal.html#!info?alias=Org_MpusillaCCMP1545 [5]
17	>GtRh1	146834	*Guillardia theta*	https://phycocosm.jgi.doe.gov/pages/search-for-genes.jsf?organism=Guith1 [6]
18	>GtRh2	148916	*Guillardia theta*	https://phycocosm.jgi.doe.gov/pages/search-for-genes.jsf?organism=Guith1 [6]
19	>GtRh3	148915	*Guillardia theta*	https://phycocosm.jgi.doe.gov/pages/search-for-genes.jsf?organism=Guith1 [6]
20	>GtRh4	107802	*Guillardia theta*	https://phycocosm.jgi.doe.gov/pages/search-for-genes.jsf?organism=Guith1 [6]
21	>GtRh5	122016	*Guillardia theta*	https://phycocosm.jgi.doe.gov/pages/search-for-genes.jsf?organism=Guith1 [6]
22	>GtRh6	149064	*Guillardia theta*	https://phycocosm.jgi.doe.gov/pages/search-for-genes.jsf?organism=Guith1 [6]
23	>GtRh7	138313	*Guillardia theta*	https://phycocosm.jgi.doe.gov/pages/search-for-genes.jsf?organism=Guith1 [6]
24	>GtRh8	145205	*Guillardia theta*	https://phycocosm.jgi.doe.gov/pages/search-for-genes.jsf?organism=Guith1 [6]
25	>GtRh9	144198	*Guillardia theta*	https://phycocosm.jgi.doe.gov/pages/search-for-genes.jsf?organism=Guith1 [6]
26	>GtRh10	144226	*Guillardia theta*	https://phycocosm.jgi.doe.gov/pages/searc

				h-for-genes.jsf?organism=Guith1 [6]
27	>OtRh1	46936	*Ostreococcus tauri*	https://phycocosm.jgi.doe.gov/Ostta1115_2/Ostta1115_2.home.html [7]
28	>OtRh2	199419	*Ostreococcus tauri*	https://phycocosm.jgi.doe.gov/Ostta1115_2/Ostta1115_2.home.html [7]
29	>OlRh1	25667	*Ostreococcus lucimarinus*	https://phycocosm.jgi.doe.gov/Ost9901_3/Ost9901_3.home.html [8]
30	>OlRh2	89413	*Ostreococcus lucimarinus*	https://phycocosm.jgi.doe.gov/Ost9901_3/Ost9901_3.home.html [8]
31	>OlRh3	28080	*Ostreococcus lucimarinus*	https://phycocosm.jgi.doe.gov/Ost9901_3/Ost9901_3.home.html [8]
32	>OlRh4	47806	*Ostreococcus lucimarinus*	https://phycocosm.jgi.doe.gov/Ost9901_3/Ost9901_3.home.html [8]
33	>DsRh1	Dusal.0121s00015.1	*Dunaliella salina*	https://phytozome.jgi.doe.gov/pz/portal.html#!info?alias=Org_Dsalina [9]
34	>KnRh1	kfl00193_0190_v1.1	*Klebsormidium nitens*	http://www.plantmorphogenesis.bio.titech.ac.jp/cgi-bin/blast/blast_www_klebsormidium.cgi [10]
35	>KnRh2	kfl00421_0020_v1.1	*Klebsormidium nitens*	http://www.plantmorphogenesis.bio.titech.ac.jp/cgi-bin/blast/blast_www_klebsormidium.cgi [10]
36	>KnRh3	kfl00037_0310_v1.1	*Klebsormidium nitens*	http://www.plantmorphogenesis.bio.titech.ac.jp/cgi-bin/blast/blast_www_klebsormidium.cgi [10]
37	>GpRh1	KXZ47652.1	*Gonium pectorale*	https://www.ncbi.nlm.nih.gov/protein [11]
38	>GpRh2	KXZ46245.1	*Gonium pectorale*	https://www.ncbi.nlm.nih.gov/protein [11]
39	>GpRh3	KXZ47741.1	*Gonium pectorale*	https://www.ncbi.nlm.nih.gov/protein [11]
40	>GpRh4	KXZ54193.1	*Gonium pectorale*	https://www.ncbi.nlm.nih.gov/protein [11]
41	>GpRh5	KXZ55246.1	*Gonium pectorale*	https://www.ncbi.nlm.nih.gov/protein [11]
42	>CsRh1	PRW60699.1	*Chlorella sorokiniana*	https://www.ncbi.nlm.nih.gov/protein [12]
43	>ApRh1	XP_011395544.1	*Auxenochlorella protothecoides*	https://www.ncbi.nlm.nih.gov/protein [13]
44	>AsRh1	5151	*Asterochloris species/ Asterochloris glomerata*	https://phycocosm.jgi.doe.gov/Astpho2/Astpho2.home.html [14]
45	>AsRh2	77589	*Asterochloris species/ Asterochloris glomerata*	https://phycocosm.jgi.doe.gov/Astpho2/Astpho2.home.html [14]
46	>AsRh3	6282	*Asterochloris species/ Asterochloris glomerata*	https://phycocosm.jgi.doe.gov/Astpho2/Astpho2.home.html [14]
47	>AsRh4	6192	*Asterochloris species/ Asterochloris glomerata*	https://phycocosm.jgi.doe.gov/Astpho2/Astpho2.home.html [14]
48	>BgRh1	89464	*Bigelowiella natans*	https://phycocosm.jgi.doe.gov/Bigna1/Bigna1.
49	>BgRh2	139324	*Bigelowiella natans*	https://phycocosm.jgi.doe.gov/Bigna1/Bigna1.
50	>TsRh1	AGF84747.1	*Tetraselmis subcordiformis*	https://www.ncbi.nlm.nih.gov/protein [15]

References:

[1] National Center for Biotechnology Information (NCBI). Bethesda (MD): National Library of Medicine (US), National Center for Biotechnology Information; [1988] – [cited 2020 Oct 21]. Available from: https://www.ncbi.nlm.nih.gov/.

[2] M. Luck, T. Mathes, S. Bruun, R. Fudim, R. Hagedorn, T.M.T. Nguyen, S. Kateriya, J.T.M. Kennis, P.

Hildebrandt, P. Hegemann, A Photochromic Histidine Kinase Rhodopsin (HKR1) That Is Bimodally Switched by Ultraviolet and Blue Light, J. Biol. Chem. 287 (2012) 40083–40090. https://doi.org/10.1074/jbc.M112.401604.

[3] S.S. Merchant, S.E. Prochnik, O. Vallon, E.H. Harris, S.J. Karpowicz, G.B. Witman, A. Terry, A. Salamov, L.K. Fritz-Laylin, L. Maréchal-Drouard, W.F. Marshall, L. Qu, D.R. Nelson, A.A. Sanderfoot, M.H. Spalding, V. V Kapitonov, Q. Ren, P. Ferris, E. Lindquist, H. Shapiro, S.M. Lucas, J. Grimwood, J. Schmutz, P. Cardol, H. Cerutti, G. Chanfreau, C.-L. Chen, V. Cognat, M.T. Croft, R. Dent, S. Dutcher, E. Fernández, H. Fukuzawa, D. González-Ballester, D. González-Halphen, A. Hallmann, M. Hanikenne, M. Hippler, W. Inwood, K. Jabbari, M. Kalanon, R. Kuras, P.A. Lefebvre, S.D. Lemaire, A. V. Lobanov, M. Lohr, A. Manuell, I. Meier, L. Mets, M. Mittag, T. Mittelmeier, J. V Moroney, J. Moseley, C. Napoli, A.M. Nedelcu, K. Niyogi, S. V Novoselov, I.T. Paulsen, G. Pazour, S. Purton, J.-P. Ral, D.M. Riaño-Pachón, W. Riekhof, L. Rymarquis, M. Schroda, D. Stern, J. Umen, R. Willows, N. Wilson, S.L. Zimmer, J. Allmer, J. Balk, K. Bisova, C.-J. Chen, M. Elias, K. Gendler, C. Hauser, M.R. Lamb, H. Ledford, J.C. Long, J. Minagawa, M.D. Page, J. Pan, W. Pootakham, S. Roje, A. Rose, E. Stahlberg, A.M. Terauchi, P. Yang, S. Ball, C. Bowler, C.L. Dieckmann, V.N. Gladyshev, P. Green, R. Jorgensen, S. Mayfield, B. Mueller-Roeber, S. Rajamani, R.T. Sayre, P. Brokstein, I. Dubchak, D. Goodstein, L. Hornick, Y.W. Huang, J. Jhaveri, Y. Luo, D. Martínez, W.C.A. Ngau, B. Otillar, A. Poliakov, A. Porter, L. Szajkowski, G. Werner, K. Zhou, I. V Grigoriev, D.S. Rokhsar, A.R. Grossman, The Chlamydomonas genome reveals the evolution of key animal and plant functions., Science. 318 (2007) 245–50. https://doi.org/10.1126/science.1143609.

[4] S.E. Prochnik, J. Umen, A.M. Nedelcu, A. Hallmann, S.M. Miller, I. Nishii, P. Ferris, A. Kuo, T. Mitros, L.K. Fritz-Laylin, U. Hellsten, J. Chapman, O. Simakov, S.A. Rensing, A. Terry, J. Pangilinan, V. Kapitonov, J. Jurka, A. Salamov, H. Shapiro, J. Schmutz, J. Grimwood, E. Lindquist, S. Lucas, I. V Grigoriev, R. Schmitt, D. Kirk, D.S. Rokhsar, Genomic analysis of organismal complexity in the multicellular green alga Volvox carteri., Science. 329 (2010) 223–6. https://doi.org/10.1126/science.1188800.

[5] A.Z. Worden, J. Lee, T. Mock, P. Rouzé, M.P. Simmons, A.L. Aerts, A.E. Allen, M.L. Cuvelier, E. Derelle, M. V Everett, E. Foulon, J. Grimwood, H. Gundlach, B. Henrissat, C. Napoli, S.M. McDonald, M.S. Parker, S. Rombauts, A. Salamov, P. Von Dassow, J.H. Badger, P.M. Coutinho, E. Demir, I. Dubchak, C. Gentemann, W. Eikrem, J.E. Gready, U. John, W. Lanier, E.A. Lindquist, S. Lucas, K.F.X. Mayer, H. Moreau, F. Not, R. Otillar, O. Panaud, J. Pangilinan, I. Paulsen, B. Piegu, A. Poliakov, S. Robbens, J. Schmutz, E. Toulza, T. Wyss, A. Zelensky, K. Zhou, E.V. Armbrust, D. Bhattacharya, U.W. Goodenough, Y. Van de Peer, I. V Grigoriev, Green evolution and dynamic adaptations revealed by genomes of the marine picoeukaryotes Micromonas., Science. 324 (2009) 268–72. https://doi.org/10.1126/science.1167222.

[6] B.A. Curtis, G. Tanifuji, F. Burki, A. Gruber, M. Irimia, S. Maruyama, M.C. Arias, S.G. Ball, G.H. Gile, Y. Hirakawa, J.F. Hopkins, A. Kuo, S.A. Rensing, J. Schmutz, A. Symeonidi, M. Elias, R.J.M. Eveleigh, E.K. Herman, M.J. Klute, T. Nakayama, M. Oborník, A. Reyes-Prieto, E.V. Armbrust, S.J. Aves, R.G. Beiko, P. Coutinho, J.B. Dacks, D.G. Durnford, N.M. Fast, B.R. Green, C.J. Grisdale, F. Hempel, B. Henrissat, M.P. Höppner, K.-I. Ishida, E. Kim, L. Kořený, P.G. Kroth, Y. Liu, S.-B. Malik, U.G. Maier, D. McRose, T. Mock, J.A.D. Neilson, N.T. Onodera, A.M. Poole, E.J. Pritham, T.A. Richards, G. Rocap, S.W. Roy, C. Sarai, S. Schaack, S. Shirato, C.H. Slamovits, D.F. Spencer, S. Suzuki, A.Z. Worden, S. Zauner, K. Barry, C. Bell, A.K. Bharti, J.A. Crow, J. Grimwood, R. Kramer, E. Lindquist, S. Lucas, A. Salamov, G.I.

McFadden, C.E. Lane, P.J. Keeling, M.W. Gray, I. V. Grigoriev, J.M. Archibald, Algal genomes reveal evolutionary mosaicism and the fate of nucleomorphs., Nature. 492 (2012) 59–65. https://doi.org/10.1038/nature11681.

[7] R. Blanc-Mathieu, M. Krasovec, M. Hebrard, S. Yau, E. Desgranges, J. Martin, W. Schackwitz, A. Kuo, G. Salin, C. Donnadieu, Y. Desdevises, S. Sanchez-Ferandin, H. Moreau, E. Rivals, I. V. Grigoriev, N. Grimsley, A. Eyre-Walker, G. Piganeau, Population genomics of picophytoplankton unveils novel chromosome hypervariability., Sci. Adv. 3 (2017) e1700239. https://doi.org/10.1126/sciadv.1700239.

[8] B. Palenik, J. Grimwood, A. Aerts, P. Rouzé, A. Salamov, N. Putnam, C. Dupont, R. Jorgensen, E. Derelle, S. Rombauts, K. Zhou, R. Otillar, S.S. Merchant, S. Podell, T. Gaasterland, C. Napoli, K. Gendler, A. Manuell, V. Tai, O. Vallon, G. Piganeau, S. Jancek, M. Heijde, K. Jabbari, C. Bowler, M. Lohr, S. Robbens, G. Werner, I. Dubchak, G.J. Pazour, Q. Ren, I. Paulsen, C. Delwiche, J. Schmutz, D. Rokhsar, Y. Van de Peer, H. Moreau, I. V. Grigoriev, The tiny eukaryote Ostreococcus provides genomic insights into the paradox of plankton speciation., Proc. Natl. Acad. Sci. U. S. A. 104 (2007) 7705–10. https://doi.org/10.1073/pnas.0611046104.

[9] J.E.W. Polle, K. Barry, J. Cushman, J. Schmutz, D. Tran, L.T. Hathwaik, W.C. Yim, J. Jenkins, Z. McKie-Krisberg, S. Prochnik, E. Lindquist, R.B. Dockter, C. Adam, H. Molina, J. Bunkenborg, E. Jin, M. Buchheim, J. Magnuson, Draft nuclear genome sequence of the halophilic and ceta-Carotene-accumulating green alga Dunaliella salina strain CCAP19/18., Genome Announc. 5 (2017) 17–19. https://doi.org/10.1128/genomeA.01105-17.

[10] K. Hori, F. Maruyama, T. Fujisawa, T. Togashi, N. Yamamoto, M. Seo, S. Sato, T. Yamada, H. Mori, N. Tajima, T. Moriyama, M. Ikeuchi, M. Watanabe, H. Wada, K. Kobayashi, M. Saito, T. Masuda, Y. Sasaki-Sekimoto, K. Mashiguchi, K. Awai, M. Shimojima, S. Masuda, M. Iwai, T. Nobusawa, T. Narise, S. Kondo, H. Saito, R. Sato, M. Murakawa, Y. Ihara, Y. Oshima-Yamada, K. Ohtaka, M. Satoh, K. Sonobe, M. Ishii, R. Ohtani, M. Kanamori-Sato, R. Honoki, D. Miyazaki, H. Mochizuki, J. Umetsu, K. Higashi, D. Shibata, Y. Kamiya, N. Sato, Y. Nakamura, S. Tabata, S. Ida, K. Kurokawa, H. Ohta, Klebsormidium flaccidum genome reveals primary factors for plant terrestrial adaptation., Nat. Commun. 5 (2014) 3978. https://doi.org/10.1038/ncomms4978.

[11] E.R. Hanschen, T.N. Marriage, P.J. Ferris, T. Hamaji, A. Toyoda, A. Fujiyama, R. Neme, H. Noguchi, Y. Minakuchi, M. Suzuki, H. Kawai-toyooka, D.R. Smith, H. Sparks, J. Anderson, R. Bakaric, V. Luria, A. Karger, M.W. Kirschner, P.M. Durand, R.E. Michod, H. Nozaki, B.J.S.C. Olson, The Gonium pectorale genome demonstrates co-option of cell cycle regulation during evolution of multicellularity, Nat. Commun. 7 (2016) 11370. https://doi.org/10.1038/ncomms11370.

[12] M.B. Arriola, N. Velmurugan, Y. Zhang, M.H. Plunkett, H. Hondzo, B.M. Barney, Genome sequences of Chlorella sorokiniana UTEX 1602 and Micractinium conductrix SAG 241.80: implications to maltose excretion by a green alga., Plant J. 93 (2018) 566–586. https://doi.org/10.1111/tpj.13789.

[13] C. Gao, Y. Wang, Y. Shen, D. Yan, X. He, J. Dai, Q. Wu, Oil accumulation mechanisms of the oleaginous microalga Chlorella protothecoides revealed through its genome, transcriptomes, and proteomes., BMC Genomics. 15 (2014) 582. https://doi.org/10.1186/1471-2164-15-582.

[14] D. Armaleo, O. Müller, F. Lutzoni, Ó.S. Andrésson, G. Blanc, H.B. Bode, F.R. Collart, F. Dal Grande, F. Dietrich, I. V. Grigoriev, S. Joneson, A. Kuo, P.E. Larsen, J.M. Logsdon, D. Lopez, F. Martin, S.P. May, T.R. McDonald, S.S. Merchant, V. Miao, E. Morin, R. Oono, M. Pellegrini, N. Rubinstein, M.V. Sanchez-Puerta, E. Savelkoul, I. Schmitt, J.C. Slot, D. Soanes, P. Szövényi, N.J. Talbot, C. Veneault-Fourrey, B.B. Xavier, The lichen symbiosis re-viewed through the genomes of Cladonia grayi

and its algal partner Asterochloris glomerata., BMC Genomics. 20 (2019) 605. https://doi.org/10.1186/s12864-019-5629-x.

[15] O.A. Sineshchekov, E.G. Govorunova, J. Wang, H. Li, J.L. Spudich, Intramolecular proton transfer in channelrhodopsins., Biophys. J. 104 (2013) 807–17. https://doi.org/10.1016/j.bpj.2013.01.002.

Sequence of modular rhodopsin and reference sequences used in this analysis

>KnRh3_GAQ79757.1_Klebsormidium nitens kfl00037_0310_v1.1

MTVDAHSTVDAHSTVTDSHSAGNGTESCYVADFLGMHHESHEGALYSVYKSLEWGCFLISIGLFVFYLQQYRKKTAGWEVIYIAFIESFKYIFEI
FWPHNNPAQLNIYGVNKSVPWVRYMEWMITCPVILMALSNISGEEGEYTHRSMQLLATDQGAILCAITAAASEGAISAVFYAIGVCYGICTFY
FCLQIYIEAYFTLPETCHSAVKWMAVIFYAGWLCYPCFFLAGSEGWGNLSYEGSAIGHCIADLLSKNAWGVMHWWIRCQLEEYKHTHNGQL
PHYSLETRAKMRAEAGHIIAGSLGSVLHVAGHQHHHFHFDEDSGMQGAQIQGQPLAQANERANLKASLLNGNAVNYNQAVQAQPQAFQ
IQSGEDGYVISMNQGESSASGSRRTTQNIEGPKKDPVASAFSKALGSMKSKVTEHFTKDLPPSARPDSPSRRGSARGGADAPTAQPQQSMKIDL
RDLANNPQLQQQLSMMLKGQMAGGYALKTDLKDLANNPQLQQQLSMMLKGQMGAGNSNTNQGSNNGSPRARPVSEELAQPARLQKA
QSARATSNLDDEIEEQAPAQMSRRPSARVPRATAAADEEQAPARTLSRKISRGDGGEPVSEERLQKRPSYRAAKAAAEAADEDMTDAPAPAL
ARRPSARAPAADADEETAPVRTASLRRAGSKSPGNSLPSSPSRRVSRANSLRAPVSASVLLCDADGDMGDFFLDQFAAVPNSKCSVKVVATPD
ELIDALATGRAYDFVMVPKGVITSDPEISEEIRAHAQTPLVAFGPALEGMAVAEVKAHMFELDVDDYIGLPKRGQEYPVEDLEALVWKYKKV
ADDSN

>TsRh1_AGF84747.1_Tetraselmis subcordiformis

MGFQLNPEYLNETILLDDCTPIYLNVGPLWEQKVARGTQWFGVILSLAFLIYYIWITYKATCGWEELYVCTIEFCKIVIELYFEFSPPAMIYQTNG
EVTPWLRYAEWLLTCPVILIHLSNITGLNDDYSGRTMSLITSDLGGICMAVTSALSKGWLKWLFFVIGCCYGASTFYHAALIYIESYYTMPHGVC
KNMVLAMAAVFFTSWFMFPGLFLAGPEGTNALSWAGSTIGHTVADLLSKNAWGMIGHFLRLEIHKHIIIHGDVRRPITVNTLGREVTVSCFV
DKEEEDEDERISTKTYANRASFMKMRNDMEQRGIQTRKSLEMLAPPPALNDGSIVLAVADPMTLTFFTQQLSQLDATIRATPAMGQGQLEQV
LEKGGFDGVLVSPEYIQQVGLVQRLKDKYHMPVYAFGWGKSSPWRSVIEGSGVDGWLEGPYFGSTFDTDALSDAIAEMQRIKTSYSMVVNGV
GMNGAGMNGVGMNGMGMDGVGMNGAGMNGVGMNGMGGYGSVGNNMHSMPMMSQQAVMMPQSAPQMMGGMSQTQQPAMMG
GMQGASPHYSVGNLQNMEGQQAVGSPQVLASSWQQSALHGGMGQQQQYGQVQQMPMMVGMQTPASPGGVQTPPHTMAGQPQMSPQ
QLQQQLYFMQQLQQRQLQQQQYQGGTGQR

>**GpRh1**_KXZ47652.1_Gonium pectorale

MSELRQAWGTVANVSQWFAFGVSVAILLFYAYEAYIATCGWEEVYVCCVELTKVVLEYFHEFDSPAMLYLGVGTRVQWLRYSEWLLTCPVIL
IHLSNLTGLKEDYSFKTMRLLVSDIGTIVFGATSAVCTGYLKWVFFAIGCVYGGFTFFTAGKVYREAYEAAPAGRCKWLVKVNAVIFFSSWTM
YPVLFVLGPEGTGVISLLESTTAHTFVDLMSKNIWGLVGHTLRVAVWRHILTNGEASAGTMRQSISVSRAEVGKAMQRMSIGSQYGGANRVSI
GSQNGGTASGPAGGNRLSIGSQSGILIPTGAATGAATGGRNSVCSQTGMAVPAVSTSGIVWTTTGAADQDDHKNRSSFWPSVGKGSAAVAP
MPPTVSVKSRANTSTGMGGGNGSGKDHDAGKGHHSDVVVGSNMVIAKVGVSGAAADSPSHGAASPAATGRLISLPGPPAQPQTPQHVQRP
GTPPTSEPSGGLQSAASAGGWTDAGAPQGSAPSPLPPPPQPQQRTATATGMGMEGAAGAARKSSSSMRLNVVAAAAVPISGPVDAIEDVEEE
EVPPPTVPVEPPPPSDRRRSSVGDPASRPYTSQQRQQAQSPRTYPHLTAADI

>Cop5_AAQ16277.3

MPATSRRFGVLLYLLASYLICTSNLGDPELTAQFHAQADPNAYTWPMVAFGTAFGLNFITLLFERESAKFQLALLACYINFLAGFSDYMSWKG
YAPIVRDSWGQGFQLLRTV**M**WLL**T**TPAMVY**L**LSIISDFSRLKVYSVMLADVLMITFGILAFLAYNKVMSILFYMVAWCLFAYVVHSMWSMFH
ASIAEARHDSSRVSLEVLRLFAVGLWFTFPVIWIVVK**M**GLVDIRT**E**EWTWCAC**D**FLG**K**VMFSSSLLHGNFLTIEQRRLIAMRIVEEGNRIQVIQE
LKDLVEQKERFMSSMSHELRTPLNGIIGLSDALLVGSCGEINDQALKTITTIKTSGARLLNLINDILDAASMRKGKLTIKHEKVNLKRVVDDVID

LCQPLAKRGVKLVNDLRENVPFVLGDTGRIIQVFHNLIGNSCKFTHSGNISISAAVKDDEVEVAVADTGIGIPEDKFDQIFLAFEQVDMSVTRK
YGGTGLGLNLVKQLVEAHGGRISVKSRENQGTTFYFTLKIHSEHPNEGQPQMTPTESVAAIPPPEHSQLATVRGTAPSAGGLPPSSSGATAGPR
RAPSRRGSFTDKMLGALGAGMGGHHKKPPSETESRDGPYASNAGPGGGGNAGQGSAMVRRVSRDAHGSNPPNPKSVTAGPQVSGPMSLSD
AAALMKGALKRKSSFRERGAKVRVLSVDDDPVNQLVIQNLLAPVGYEILQAMDGQEALKVLTEEERLPDVILLDVMMPGMSGYEVCRKLRE
MYPLSCIPVIMISAKSKEEHIVEGLAAGSNDYVVKPFGRQEILARIAAHLRFRDTVYQAGEIAGAIPGDVRPERVLLRGGANGGGLDLTPFLTGP
ARFTSLPPRIARGI**EAGTTSTTLQMFESLTLLEVRLVNLGDLLASVPASDLLVALASLFHDLDTLLEQHGCYLLEGLDESHLIVSGLDNVGD**
QVLHALGLARSLIAAADTFALGGRRSKLHLAVGVHTGPAQGVLVGYSHPLIFFTGQLPAEVHMLQATCPPNCVHVSARVLESVAHSER
EHFVPAGVMASGATTYLMKVGGWEGGGIVAASEATSRWSKAKRGMHDGLATDARRMRPIQLALLDMAQANPAFLVDIASAAHGAAGGG
AGTSSDGQGERGGGGGGSEELSKLKREYESLERQMEEVSGEAARLQDLVDELEEQLLARSNTAAASTAATAAASQQAAVAAADAASLKAH
VAELEGQLAEAARERNGLEAALAEMEQRLVATHAALATANHEAADRARAHTPAPQELPPHHPHSASRSHLHVLPQQNTPRVETASMAPS
ASGSEANFASGLSALGGGSLFGGGALMPRGAMPLGVPMRMPNLAALRAHAGDMRGLLDELGLGSLASRFEAEDVSPGLLPYLDDAAMREL
GASSVGARLKLRLAAQALFM

>Cop6_Cre11.g467678.t1.1

MKLRQRTVGAQLRSQPVSSAGGPANSGPPATPSGGIAPVSIFGAAEALADPEARGWILTWSWTFTGFFVYITASWLSGLWYTTDPLAYAALRA
QVPTLVYQMSSTAFFTALVLNLTSLLFEDNAPKRQLALLSCAIKGAACHTDLLLVTGGATVLYDAYGSICIPQRYV**Q**WLV**T**TPTMVY**I**LSKISD
FTPRQTATAIGLDVLMVLSGLVANFLRSPYLWVAFLTSTAA**FIGVLYMMGLMVYSAVKEHTSANSRRSLLFIYMCTLFIWNLFPLAWILHV**
VHRGSPAAEYLNVFANFMAKVLFSSSIMYGNYMTIAQRRLLAQQDAENANRVQMIQDLRDSVTRKDQFMSLMSHELRTPLNGIIQLSDAL
VRGAGGEMNPKGQHFVRTIKNSSNHLLNIINDILDVAALKEGKLTIKHEVCSLAKAVDHVVDIVAPLAKKEVTMERWVDPATPLIIADFSRVI
QILYNLTGNALKFTNKGRVGVRVEPSADGTHVLLQVSDTGIGIPKDRLHSIWGAFEQVDMSVTRKYGGTGLGLNIVKQLVEAHEGTIEVASVE
GRGTTFTVELPVLQSSTRRSLEGQVLDSLTRCGHAAARDTMVQRRTRSRPSLGLEDTITQFARGVQRRASGLLGVAKAAQEAGSNAGTGPAG
STGAGAGGGGGAGGGGQRDSLDREEGEQLLRKRTHELEHESRLGDLARRTVHKQSMEEADLASRQLLLSDYERRAERDTRSLERIDSGQPGL
TNGGGEGAGGAAGGGAGSGGGGGSAPGSAGKAGVAGGSCRGDVRGGGTDGRGGGGGGAGGAGGGGGGGNGGGGASGGGGRRSGAPT
SGRASLGELPSGGSGGGGGGGGGGGDGTPESPRSAIARRGLLAMRQSSLSNLRGATSARAGGAAGGAAASGGGVSVGRWASTTDTGFANQ
GAAAAAWRGDSHRTLPGVEGGCVSVSSANGNSIADVYEALQLARASNESGGGGGGGGGGGGGGNGSSLKASGGSLALRMSAYGRTGYGG
ANGGGGGGANGLNGYGGGGGALGGSGASGSLLKSALESDLYRNGPSPRDPYDCDASSVGADSEYEWVGDGGGAGGGGAFGGRSRGPTST
GSLALGGMACGPGGRRHQPPKPSLRSGKLTPMLSNAAAAAAAITAVPPGLTLDKLAYSDMYGTIQVLSVDDEDINQIVLEEILTDSGYAFARC
MDGAEALEWLCASDTMPDLILLDCMMPVMSGHEFCATLRKVIPGNVLPVIMVSAKSDEENIVEGLRSGSNDFVRKPYQREELLARIETQLRLK
SDSWWLAELVNNVDGRETESMKLLKNILPESIIARMQQGQKFVADSHGHVVILFSDIVGFTSLSSKLPTAEVFLMLSNMFTAFDKLTDRFSVYK
VETIGDAYMVAAGHDEDEDKEAKGSPLMRVLGFARAMLDVVRNITAPNGERLRIRIGVHCGPAFAGVIGMKCPRYCFLGDTVNTASRMEST
GFPMCIHVSENVFKHHPAAEAELQEVGERDIKGKGHMRTYVVRTGAWEQALRDFAARQQAAAAAAQAQAQTLALARQQAALLQQQHE
QLQLQLQANGGGGGGGATANAAKAAADALAQLPASLSFDSASAGSTSSMLPLSGGSVAGAAARTANSLAPGAGGGGAAAGAADGGGGG
NGCGGGGGAVRMGHLLSTVAEEGSLPGSPSSFAAAAAAAAALSPSAAAQQQQQQAPGYHPSHLRTASAGAGRSPLSRGELPAAASSPLQPGG
GAAAGAAGSSPLLPPLLRGANHYTDPRLAGGLRPSFSTGGYVLEDDSDDGGTSTNTGVGGGTGHGMGSSLGEGRAGSISLTGIGARSFTGASC
GGGAGGGGGGGGLRMLNSPTGQYDTGDAGENGSSGPGGSGGGGGEVGGSGGTPSGLRVHVPRSLSGLSGGSYGSGGSTGIGMSRAGLGSAT
AAAAAASGACFLTSDAADASFSGGVLAATTSHAALGSGGAADSQHAPSAAGAVMGSSPAAALQVSPASPGGAAAGGLGSGLSPQSSLYGG
AQLLLSPNSAGLHSGHANTTLAYLEQRIASLGTQLATEALSRQRLQDELDAERRRAAGAMQQASLLMQQLRNATQAAGGGSGGAPSSVAA
AAAAAGQEQHLARLQLGSGGGGADAASGGGGGAALPPSAAVPLSRLPPPPRGAGGAVASLRAAGGANELLPTSNEANSNADADVIIGGG
GMSSTVVPAAQPPSSSAFGSAGAATSGGSGNDDQEITLASPFVSDLPAYALEAGDVVPNSLDFASQGFDPGSSGAGGRGGGGGSRHAGRAAA
AQQRPSSKVQRMNVGDAAALMLQPPKAQQLQPGSQSTAFVEAGGVGRSGASGGGIAEPLPGSAAEAYSGYSRQQLEDQLLLPVPMPTSITAL
MPGGGTAAATAAVGTATAASTTTTTTTTVSHHRHVFHTSAAAGLALSAHPAPGSSPLPAPSSSSPCTALQPLQLQPPPLPCYSLDALFVDLGL
EPYLPRFRDEAIRLDMLLSMDAQQLERLGLKPLGYRIRVREAVVELARGLLRSSEDAAVLVEMSQQHQHQQQYRSQQAQQLQQQHQPLER*

>Cop7_Cre01.g038050.t1.1

MGALPEAERSSAHLIWTLGPRRNFVGQLYSVDTPVLLWCCLGSLSYACCALGLHQLDSTVREESDQGQEAVEWTIQVQTAAFGAVLLLNVL
NLGMRQHLLPFKCNLLLLFINAVAFATDLIIWRRGTPLYPLSPGLGLGLLRPGAAAAAGDRAFVPLRYV**Q**WLH**S**TPTMIY**M**VGLLAEVRGW
QLALPVLCDVAMVVTGLAACCTTGLTKAFFTAVSF**ATFAVVLRRIHAMFDKTLRSGDVAADQRGTLRAVRALLLALWGAFPLVWLAAD**
WRLVS**PQAEAVAWGVCDYLAKAVFSSQLWQSNLTGVHVRREVALEAWEAHNRVEAVARLSSLLAQ**RDELLSTLSHELRTPLAGIVALT
GALYRDVASALPASARTLGAVRAAATCMLHIVASTLDAFAGQRAERGAAGADAGAAGGSSSNATASSGSNGGQAYNQQALALKAPAGGA
AGGATGGSGGGGGGGSVSAAVVDLRPLVETVAAVLRPLAHDGVAVAAELPEGLPAVTADATRLNQVLYNLVGNALRFTSSGEVRVSARVL
GPDGAVWRCREPNSSGDSGAVGLSSDSPHAAAAAVRGRRSEDFTASSSSVARSPLPGGRNSGNNAPGSAGLWRRRPRTSASSEHSAAMAAP
GGAGGPLGAEAAAAALPPGCVVEVAVEDTGVGISQAQLDALMAPAYRPLGTADTAAAANAAAAAGYAASASIRAGDAGGTLSDDSAARK
RSNKSRRRAAKAGKAAASSSSLFYTGAPASGAISIGGTGLGLYLVRLALRAQGSELTAESAAGIGSMFRFRLPVATAACALALASKQAAQQQG
QQGQQQQQQGAAVAAEEQGAVNMAREASVAWMAAAGEGEGSQPGPTSAAAPAPAKVSTAPVVPRSEPSAIARLTCRVNGGAAAAAAD
GASSSGAADGVGAGGALMRYAQSCCSPLAATGSVPGDAGDSGGFPLPSGSTAAASHLGSGVPAMTGYLGRPPLERGLSCSACNGPGGLRAA
SGVSVVMAAGAAAAAAAAAAAAASSAVGSAGSADGALVPGGSAPRERSSLLGYPKREVLDVRNLAGACGGPAAVTGEGALVPAPVAAAAP
VANNFRHCNNGTLQVLSVDDDPINQLVAGQMLTSQQWKVVKCMDGPQALKFLRLPAAPEPRASASGGATAADALAEALAAADAAAPPL
DPVPDCVLLDVMMPGMTGFEVCRRLRRRYPHAALPVIIVSAKSDAAAVEEAFAAGADDYMTKPYKRAEMVARIKAQIRIRDSMAAAAAAG
VPGGSVEVPPLGNDLRVQARAQDPAAAAAAEPQQPAAQSRPQLHAPGGHVAGSLASLLGGAAGSDSAITPPGAYARRRSVAHASPAAAM
ASGASTPCGSNSAAATVSPPRASLNGATGAFGSRMLGGSTCVTENGMQAPAPPRGVATGPAGLGSFGGGAAAAAGLFFSPGLPSGGGGGGG
GGGGGGGGIFSATSTLTGTGQFRYVEDACASELALVTTTSITTGPTPPRAAALTVAFVDAESSGAGGAAAGALRAAATPAVAASPVSIRTAEG
GVAGLSSGKLLMLPADTASAPTVSGGLTSAPVSGAAADAAAAGVSLQQQPPPTPIPLAALSPPFPPAGGAAASPAVAASPAATAAAAAAAA
AAAAVPMDLASLMAELLAEVRRSRVLEADVSKLTARVAALQTACGTLAADRDGWCRQARDLRALLASGLAAATAGIAGGGAAAPPPSLAF
GGSPAAYGGSAAGGFTSLNLVGQPTSSAGPASVVVRANSLLNEAALAAANGSTASADSSNHALPLATYGAAANANGGGGGGGSVAAQLE
DLRRETAALRQQLRLALASIGNGSNSVSGGGGAQGSVPPVRAGSGISGGGLPSTGASAGINNGSSSSAAQMGRAYGAGAGAGAPAIPGTGAG
SAAANAANGCAATSPQPQRAVQADGNGGAPGPAMTGPVAGAAPAATSGGADAGLSAGSGLVAAPWGPHHQLLPNPLFASSTADMLLHT
AGSAASASASAGSGAAPHGLAAAATTVAAAAAAAGAGHAEPAGSVIAAKDAVAAAIDASARTADATTAAAGAADAGLPSGDVPTPTRA
NGDAGSGPGARRGGGGASAHAHAQLPPAEAQAHMGVTSLSSDLPPQVRRGNAGSNGVTATANGQSCAAGAAGADHGHGGHGCGGAA
ADAGIFRGGGVSGRSENSRGGSTPSVPTTGSVQGGDGTPKVADRALILPGAQPLTASTPVTPAAAALAAASAVAGSGRDGARGRSGGATAGI
LRLLCSRGAVPVNGVDSK*

>Cop8_Cre07.g329900.t1.1

MVGYACNMILTLLKVIFESSRSKLDLHLVSCYINALAAASYLMLWTGFSPIVPDINSCLYIPQRWL**L**YFF**T**APAIIH**I**LSQISNYSTRMRIWVVLL
NAFMLAAGGFGTVPWISWTHKVFWYTLACVPFPSILCHMWRMVTHALDETQDPSS**KRAISFIRIFSITTWNFFPIVYFAAIDGALPLEISEPL**
WAALDWLTKMVYSSSLMEANFFTTHWRELTLRAIEEANRLSTIQQLSLDIERKDEFLSAMSHELRTPLNGIIGLSDSMIAGACGPLPEKAVK
TISTVKLSGKRLLQLINDILDAAKMKQGMLVIKHEKVDIKRLVSDVLDLSLPLVRKGVRLINNVGNVPKIVGDNGRIVQILYNLVGNATKFTRA
GTVSVSAGVVEGKVYITVSDTGVGIPPDKIDKIFEAFEQVDMSTTRRFGGTGLGLHLVKELVKAHSGRISVQSKVGVGSSFTVWLPINQEEIDFG
DAFTPSRLRQASEDSNDAGSTRLGGNSVKWMDASANPAAAAGAGGDAALAAAAQAAAAARTFNREVGMGGGAARSEAEADDAAIVAA
AMEAERRAGGRSPPPMEASRNMPDDAQTLLSVTKSGFKLVKPFYRERNGGCMVLSVDDDPINQLVVENLLLPEGFKVEQAMCGSEALDWL
QHTPLLPDVILLDIMMPDMSGYEVCQEVRRRFSTVCIPILMVSANGTPEHVMKGLEAGAVDYVKKPFNRQELLGRIRAQVRNREVVDAEMET
RLVNKMLHRMLPAPVVEALQGGAGVSGFVELHEDVTLLAADVGSWLNTSGLMMSVEGCRALLGVVNGMHAAFDKLLLKYKAFRVVSTLD
HYLVVCGHDGTPDHVRRGLSLAEELVTAARSLTLPPGCGGGQLRVRCGLHTGPLTSVVVGVDSPHYAVFGETVGVVAGLVSRGFDNTVHCS
FAVHAALRIKSQDRISASFVPAHLLLKLPGKLPPVQTYLYKCGDWEQALKQLDDEEGERETAESAMAELSARERKWKEEQAAAEAAAELTA
ASLEEERAALEALAARLVTERAELEAARGEVARLRGEVEAARAGGAAASAGAALAAAEADTALAAARAEVEALTVRLAESEAERSRMKITY
EARLIEARETAAAAAASVHRSMLPMRGYGAHHEYGAVLGHRPGDSPHQQMLLAQHQQQVQQHVRPAGVSRISAASHNGAPGVSAPQA

PLVPQASGGAAGAFLPYSYPYSYTPGRRAPSIGGASSLNMSGGSFWTQPYRIESFMRDIGLPQYAAVLRSQDVTPAVLAGMRESDLEAMGVPT
LGARLRILEAARQYGSQSTVRMALLCKDGQDGADGAEQMLSDALGQGGVESAATWAHMGGGDSEDTAAGGAGSGNERL*

>Cop9_Cre15.g643503.t1.1

MPGDRQHSSVGPPVPASTSVSAPTEHLKEAYNTLVLLVSCAAIWMVCSYIDATYTPRDFNYIPSRSLIISYQWCMVGYASNLILTLMKVIFETNR
SKLKLHMVSCYINILAATSYCMLWTGFSPIVPDVQSCLYIPQRWL**L**YCF**T**APAIIY**I**LCQISDYTTRMRVWVIMLNVFMLAAGGLGTVPWISWT
HKVYWYVMSCVPFPSILVHMWRMVSSAVDETMDPTSKRAIKFIRLFSITTWNLFPFVYFAAI**D**GALSLEI**S**EPLWAAL**D**WLT**K**MVYSSSLMEA
NFFTIAQRREFAVRAIEEANRLATIQQLSTAIERKDDFLSVRKGVRLVNNVGNVPKIVGDNGRIVQILYNLVGNAAKFTRQGIISITAGVSEDNA
KVYVSVTDTGVGIPKEKISKIFGAFEQADMSTTRRYGGTGLGLHLVKELVKAHCGDITVESEVGVGSSFTVWLPIAQDGAMAGSVEESDREGR
EEKPYTLDFNGPLRRISEDSEEGGYLQAAAAAAAAGGGGGGGRSGGGGGGGTNRRSSQENRNNGGGGRAGGGGGGGGRGGERGHAESDG
EQDGPGGGGGGGGGGGGAGGEEQEGDEGTPRGGRGGGGGGRGGRGGGGGGGASNQSNHFGFYSNGDSGASGGPGGGGGGGGRGGGGG
GGQGDSGPQGDSGGGGGGGGGGGGGNGGGAGGGGSGGRGLGKGPSGRGRESSAENLLTVTKSGFKVMKPFYREKFGGCMVLSVDDDPIN
QMVVENLLSPEGYKVEQAMSGSEALDFLKRTTTLPDVILLDIMMPDMSGYEVCQEIRRRYSAVSIPIIMVSAKGHPEHVMKGLEAGSVDYVKK
PFHRQELLSRIRAQVRNREIFEAEMESRKVTEMLKRILPLSVIQRLQQGQSMIADAHDEVTVLFAGQFMIADAHDEVTVLFAGQFMIADAHDE
VTVLFAGQSMIADGEVTVLFADVAGWLSSQELSARTTSDVVIVLNEMYGAFEKLLLKYQVFRVEHTGESYLVVSGHDGTQDHMRRMLSMAA
DMLSAVQQLAFPGGEPLRLRVGLHTGPAYAGVVGIDNPRYCVFGDTVSVANALEARGFANAVHCSNAVYEAMRSKDRDRAGAMFAPARK
SMAAGPSVMDTFVYKVGNWESALKELEEEEQQKEAADREAREKDDAARAAEAEAAAAAAAAAAAAAVAEAASREREELAAACARAEE
LERKVNTLAAEKEAAEAAAAAARAAADALRARAADEAAERGRLHASIATYERLLAEAREAVTLAAARAGSAGGAGGGGAGGLAAAAAA
GAAAAAQRRGSPAGIQHHHHQQLQLQLPPNLAGSPSSMLPPGHHHGPQHAHQHAGLGAEDSGGLGLGGLGSGLGGLGSGGGGLGSGLG
GLGGGGLFSPTGSDVFPLYPGGGGGAGSVAGSGGGGSVAGQAVAQSFWTMAYPIDMFMRAAPSPSPPPDDAGPVSPRADGQDGDPALEIPG
AGVQPAGAQELEELGIPPAAAAALRDALQPQEFELPGIPEIDEERRLYESRLPGKDGAERIPRFESVRIPQVPIDPDIRSFLLDVCVRALAQREAS
PTTTESSVKAMLASVFNALDRPGASERDREIASYIPRTFKEVLTLVTVLEPACRWPQLYRYKMCKCGFIYRGAHQSASVCHGIVKAQGGGVEA
CGISCEQAREVTYSSIANYVERAYANPEIAADLSSWRQRQAFPRMVERIAARERERVAAHQAANAAAGRPGDPIPPKTPEEKAEDERDSQAST
DSMFDMVDGELVKEALRKDPAFAADPRNLLVVLVSDPFIVPADEHERSSIPYLLLPVNASPENRHHLGFATLLSLIGGNVKPPGSKKPDPLNP
NHMMALIRDELAFLEKYGIRVWDASKNEFFICRVKCISFISDYRGLHKHLGMKGSPAKCACFKCWHEGHVVGRKAVYPHNAAHLPCLHVL
RTSLVGRHVPPPKKPPKKQAAAGAAKPQPTRATAPGDGTRAAAPGAKRTAAAPPVTGAAAGGGKRLRGAQGAAGGRASDSGSSSSSDGGS
SSASEYSDVEVESEDEAKRLRRQQQQRQQQPQQPRQQQAVRGRGPAVDPNLATPPARRTKAEMWYACNSPTPGASLVPVPWRITLEDPIGL
AFPLYELDSFDPIGGINYDGMHTIYGVMRDTVVRYVQSFRKRSAAMEEYDKKNDIITVSGRGAGKVVENFRGALAVIAANVPRAIAGRLARLT
TAGKKAKSHSFFLLAGPFGAYAVKKAGMPPRLEAAMLSIVNACSRLWDKVQRRSQLGQLRTAVVEAICLVERHMSATELDIKLHNLMHLVD
GIEQLGPLFTHSMFKPESIWGQLGRWAHSKRHMEMSMFFMALDREVSLRLRQLHPASVDAPQRHKEAQRQLYVGEEARGGRAAPVLRIQAR
HDADGEEEVPDEGEATMWDEAKFFEADPVIYWGRFSGSRDWFDAESKEVEALYESAGLEHAMGSTVQSVRMLKEIYVGSVKLSRGSWFMCR
AANDLPDWWFGRVVEVVLHKGADDTERALVRAEWFTGATKEEAGAVSVPICDQILAPVVASRPANIIVETGPWYLPEAIVPWPCHLEPHPG
NPNVQVALARLWHILRNLFPYPTVADAVRVSRPFP*

>Cop10_Cre15.g643503.t2.1

MPGDRQHSSVGPPVPASTSVSAPTEHLKEAYNTLVLLVSCAAIWMVCSYIDATYTPRDFNYIPSRSLIISYQWCMVGYASNLILTLMKVIFETNR
SKLKLHMVSCYINILAATSYCMLWTGFSPIVPDVQSCLYIPQRWL**L**YCF**T**APAIIY**I**LCQISDYTTRMRVWVIMLNVFMLAAGGLGTVPWISWT
HKVYWYVMSCVPFPSILVHMWRMVSSAVDETMDPTSKRAIKFIRLFSITTWNLFPFVYFAAI**D**GALSLEI**S**EPLWAAL**D**WLT**K**MVYSSSLMEA
NFFTIAQRREFAVRAIEEANRLATIQQLSTAIERKDDFLSVRKGVRLVNNVGNVPKIVGDNGRIVQILYNLVGNAAKFTRQGIISITAGVSEDNA
KVYVSVTDTGVGIPKEKISKIFGAFEQADMSTTRRYGGTGLGLHLVKELVKAHCGDITVESEVGVGSSFTVWLPIAQDGAMAGSVEESDREGR
EEKPYTLDFNGPLRRISEDSEEGGYLQAAAAAAAAGGGGGGGRSGGGGGGGTNRRSSQENRNNGGGGRAGGGGGGGGRGGERGHAESDG
EQDGPGGGGGGGGGGGGAGGEEQEGDEGTPRGGRGGGGGGRGGRGGGGGGGASNQSNHFGFYSNGDSGASGGPGGGGGGGGRGGGGG
GGQGDSGPQGDSGGGGGGGGGGGGGNGGGAGGGGSGGRGLGKGPSGRGRESSAENLLTVTKSGFKVMKPFYREKFGGCMVLSVDDDPIN

QMVVENLLSPEGYKVEQAMSGSEALDFLKRTTTLPDVILLDIMMPDMSGYEVCQEIRRRYSAVSIPIIMVSAKGHPEHVMKGLEAGSVDYVKK
PFHRQELLSRIRAQVRNREIFEAEMESRKVTEMLKRILPLSVIQRLQQGQSMIADAHDEVTVLFADVAGWLSSQELSARTTSDVVIVLNEMYGA
FEKLLLKYQVFRVEHTGESYLVVSGHDGTQDHMRRMLSMAADMLSAVQQLAFPGGEPLRLRVGLHTGPAYAGVVGIDNPRYCVFGDTVSV
ANALEARGFANAVHCSNAVYEAMRSKDRDRAGAMFAPARKSMAAGPSVMDTFVYKVGNWESALKELEEEEQQKEAADREAREKDDAAR
AAEAEAAAAAAAAAAAAAAAVAEAASREREELAAACARAEELERKVNTLAAEKEAAEAAAAAAARAAADALRARAADEAAERGRLHASIA
TYERLLAEAREAVTLAAARAGSAGGAGGGGAGGLAAAAAAGAAAAAQRRGSPAGIQHHHHQQLQLQLPPNLAGSPSSMLPPGHHHGPQ
HAHQHAGLGAEDSGGLGLGGLGSGLGGLGSGGGGLGSGLGGLGGGGLFSPTGSDVFPLYPGGGGGAGSVAGSGGGGSVAGQAVAQSFWT
MAYPIDMFMRAAPSPSPPPDDAGPVSPRADGQDGDPALEIPGAGVQPAGAQELEELGIPPAAAAALRDALQPQEFELPGIPEIDEERRLYESRL
PGKDGAERIPRFESVRIPQVPIDPDIRSFLLDVCVRALAQREASPTTTESSVKAMLASVFNALDRPGASERDREIASYIPRTFKEVLTLVTVLEPAC
RWPQLYRYKMCKCGFIYRGAHQSASVCHGIVKAQGGGVEACGISCEQAREVTYSSIANYVERAYANPEIAADLSSWRQRQAFPRMVERIAAR
ERERVAAHQAANAAAGRPGDPIPPKTPEEKAEDERDSQASTDSMFDMVDGELVKEALRKDPAFAADPRNLLVVLVSDPFIVPADEHERSSIP
YLLLPVNASPENRHHLGFATLLSLIGGNVKPPGSKKPDPLNPNHMMALIRDELAFLEKYGIRVWDASKNEFFICRVKCISFISDYRGLHKHLG
MKGSPAKCACFKCWHEGHVVGRKAVYPHNAAHLPCLHVLRTSLVGRHVPPPKKPPKKQAAAGAAKPQPTRATAPGDGTRAAAPGAKRT
AAAPPVTGAAAGGGKRLRGAQGAAGGRASDSGSSSSSDGGSSSASEYSDVEVESEDEAKRLRRQQQQRQQQPQQPRQQQAVRGRGPAVDP
NLATPPARRTKAEMWYACNSPTPGASLVPVPWRITLEDPIGLAFPLYELDSFDPIGGINYDGMHTIYGVMRDTVVRYVQSFRKRSAAMEEYDK
KNDIITVSGRGAGKVVENFRGALAVIAANVPRAIAGRLARLTTAGKKAKSHSFFLLAGPFGAYAVKKAGMPPRLEAAMLSIVNACSRLWDK
VQRRSQLGQLRTAVVEAICLVERHMSATELDIKLHNLMHLVDGIEQLGPLFTHSMFKPESIWGQLGRWAHSKRHMEMSMFFMALDREVSLR
LRQLHPASVDAPQRHKEAQRQLYVGEEARGGRAAPVLRIQARHDADGEEEVPDEGEATMWDEAKFFEADPVIYWGRFSGSRDWFDAESKE
VEALYESAGLEHAMGSTVQSVRMLKEIYVGSVKLSRGSWFMCRAANDLPDWWFGRVVEVVLHKGADDTERALVRAEWFTGATKEEAGAV
SVPICDQILAPVVASRPANIIVETGPWYLPEAIVPWPCHLEPHPGNPNVQVALARLWHILRNLFPYPTVADAVRVSRPFP*

>Cop11_Cre17.g733150.t1.1

MLGRAFLHNPAGLAELRIQVPNAVYDRSSSIFLTALAIDVLNIVFEQQTIKLDCVLLPAFIKGMASTTNVLVRFFTPVIAMTDLGRPVVVQRYI**C**
WMH**T**TPSILM**L**LKMISTTITPRETMIAIFFDEVMVVTGVLALVTTGWQRVLWSLVTHAAMLPVVPYMHKAFTECIATHVAAAPTMAPLWAA
PSAGSAGRRGGSGAVSIGYDAVGFDGGGSGGGGRPGLLARAMEQ**IRTRPLQLTMLCIYVLNLVLWCTFAITWDLALLGWISVTMEEVLYV**
GCDFSAKVLFSSTLMLSSFKEIEARRENAMRVIEGSSKAKLIAELQALLEQKERFMSSVSHELRTPLNGIIGISEGMLSGCCGVLPEGVRRQIYII
RTSGARLLALINDVMDAAALRQNKLVLKQEHVVLRHVVDDVLDLTRSLVDGEVALVNLVPPRMMVVGDTGRIVQILNNLLGNAAKFTRRG
QIRVTARQVESGRKVAVTVSDTGIGIPRNKLATIFLPFEQVDMSISRKYGGFGLGLNIVQELVKAHGGTINVSSIEGKGSAFTFTMPLLRAMGRE
SLEEGRNAAHRAAAAGGATKMIGGTGMSTTDTSSQLGGSTSGSGGAHGTGGAGTISGAVSTATPRGDSRTPGGGGAGTNTISDYPGGDTDT
MYTRRSDVSESSRVRGGGGGGMGGESQSGADATAELEQYGELKYNAAELSALASSRPYAQSAVRDSLPSLAADGSGMLPHPSGPLSPSVGLD
GSAGGAGGAAGSGGMGRGSSSQLPTVLPAGASSAVAAAARARSRAVERGGAGGRGGTTGGGGGGAAGAGVELPGGRIMPGPKPFHYTMY
KRFQLLSVDDDPVNQSVVKSLVQGTGYEVVSARSGAEALRHVAAAPALPDLVLLDCMMPEMDGYEVLQRLRAMTPHVHLPIIMVSAQTEE
DHVVCGLDLGADDYVTKPFKRAELLARIRSQLAYGEWEQDEGVFDTISTMDGGELANVAAAAANAAAGRGPLGGPAAAPRPGAAGGGSD
DSEAGAVSAALGNLNLGPAGVTAASDGLGGASGAEQRLIVCIDDDDVNQVVLHGMLASQHYRYVRASTGAQGLAFVCGPTNGGIPPDLVL
LDCSLPDTTGFDVCRVIRQMYNKQQVPIIMLSARHNESAVVEGLKCGANTYVTKPFRRNELLARIRLHLRARDGPIQRGTSINDMGQVDESA
AAAAAAAASMVGADGAGLAFGGMLPSSSHQAGLGGGGGSYAQQASLAQPPSAHPLLLASGSGSAVFPDSAVLVVAVADYAGLCGMLLPG
EMAELSGRMLAAFERAVRAAGALPIEAGSGIMSAALLPPPPAAAEADGGAAAGLRLAALHRVGRALLDTASTIPVPGTTTCLQLQQALALG
ALTVSAGGGGGGGTTSSSFFGPVLVELLDLARRAPPMTIVTPDAEAEALRACGATADIRGPLFLEADDVGGGAGGAVAGPLVSRRLWLVEAH
PLAHQYLPPQLLYASGAARGAFFQVQPGATGAGEAAHQAAPLAAGAPGAHAGGVGVMGFGFAEQQQPGTRSGSAALLAGAGGGVGGGG
AAAAAGMVPVSVHPTPGVSAAASEAAAGSGGAGRGFGSSSIASYGAAGGGGGSAAGATAASLGLGGPGMGIGLYTNGMGVSRGVSRAFSH
TFGLDSDNHGRANSAAAGSAAGGSVAGGGSVAGGGVHTGHTAGTSGNGGSQGSVLTNGRGGGGSGGASDVAAATAMTAALPGGSAVG
GLDSSSVAGITFSLQAAAAAVAAASTGGGGPAQQHLQTPAGAGSGLHDSAGASGLYFNSSAGGGAGRSPLQPFGVVGGGGGGAGSGMTH
NSQPLQLQQQQQQQQQFDMVSAAAAEPQIRALRHELAAMRQQLEALQGAASTSSASNVMAGGGQGNGGQPQLSQQQLSQQLTQQQLSP

QMTQLLLQQQMSMHQGFVDAALAAAAAAVAAAPGGSASGVVPMTIGSGAAGLPGVASSVGSAVPPAGTEGDAAGALSTAGSAVPAEKAS
KKGGKISRLFKMGSSKSSK*

>Cop12_Cre17.g733150.t2.1

MLGRAFLHNPAGLAELRIQVPNAVYDRSSSIFLTALAIDVLNIVFEQQTIKLDCVLLPAFIKGMASTTNVLVRFFTPVIAMTDLGRPVVVQRYI**C**
WMH**T**TPSILM**L**LKMISTTITPRETMIAIFFDEVMVVTGVLALVTTGWQRVLWSLVTHAAMLPVVPYMHKAFTEAMEQIRTRPLQLT**MLCIYV**
LNLVLWCTFAITWDLALLGWISVTMEEVLYVGCDFSAKVLFSSTLMLSSFKEIEARRENAMRVIEGSSKAKLIAELQALLEQKERFMSSVS
HELRTPLNGIIGISEGMLSGCCGVLPEGVRRQIYIIRTSGARLLALINDVMDAAALRQNKLVLKQEHVVLRHVVDDVLDLTRSLVDGEVALVN
LVPPRMMVVGDTGRIVQILNNLLGNAAKFTRRGQIRVTARQVESGRKVAVTVSDTGIGIPRNKLATIFLPFEQVDMSISRKYGGFGLGLNIVQE
LVKAHGGTINVSSIEGKGSAFTFTMPLLRAMGRESLEEGRNAAHRAAAAGGATKMIGGTGMSTTDTSSQLGGSTSGSGGAHGTGGAGTISGA
VSTATPRGDSRTPGGGGAGTNTISDYPGGDTDTMYTRRSDVSESSRVRGGGGGGMGGESQSGADATAELEQYGELKYNAAELSALASSRPYA
QSAVRDSLPSLAADGSGMLPHPSGPLSPSVGLDGSAGGAGGAAGSGGMGRGSSSQLPTVLPAGASSAVAAAARARSRAVERGGAGGRGGTT
GGGGGGAAGAGVELPGGRIMPGPKPFHYTMYKRFQLLSVDDDPVNQSVVKSLVQGTGYEVVSARSGAEALRHVAAAPALPDLVLLDCMM
PEMDGYEVLQRLRAMTPHVHLPIIMVSAQTEEDHVVCGLDLGADDYVTKPFKRAELLARIRSQLAYGEWEQDEGVFDTISTMDGGELANVA
AAAANAAAGRGPLGGPAAAPRPGAAGGGSDDSEAGAVSAALGNLNLGPAGVTAASDGLGGASGAEQRLIVCIDDDDVNQVVLHGMLAS
QHYRYVRASTGAQGLAFVCGPTNGGIPPDLVLLDCSLPDTTGFDVCRVIRQMYNKQQVPIIMLSARHNESAVVEGLKCGANTYVTKPFRRNE
LLARIRLHLRARDGPIQRGTSINDMGQVDESAAAAAAAAASMVGADGAGLAFGGMLPSSSHQAGLGGGGGSYAQQASLAQPPSAHPLLLA
SGSGSAVFPDSAVLVVAVADYAGLCGMLLPGEMAELSGRMLAAFERAVRAAGALPIEAGSGIMSAALLPPPPAAAEADGGAAAGLRLAAL
HRVGRALLDTASTIPVPGTTTCLQLQQALALGALTVSAGGGGGGGTTSSSFFGPVLVELLDLARRAPPMTIVTPDAEAEALRACGATADIRGP
LFLEADDVGGGAGGAVAGPLVSRRLWLVEAHPLAHQYLPPQLLYASGAARGAFFQVQPGATGAGEAAHQAAPLAAGAPGAHAGGVGV
MGFGFAEQQQPGTRSGSAALLAGAGGGVGGGGAAAAAGMVPVSVHPTPGVSAAASEAAAGSGGAGRGFGSSSIASYGAAGGGGGSAAGA
TAASLGLGGPGMGIGLYTNGMGVSRGVSRAFSHTFGLDSDNHGRANSAAAGSAAGGSVAGGGSVAGGGVHTGHTAGTSGNGGSQGSVLT
NGRGGGGSGGASDVAAATAMTAALPGGSAVGGLDSSSVAGITFSLQAAAAAVAAASTGGGGPAQQHLQTPAGAGSGLHDSAGASGLYFN
SSAGGGAGRSPLQPFGVVGGGGGGAGSGMTHNSQPLQLQQQQQQQQFDMVSAAAAEPQIRALRHELAAMRQQLEALQGAASTSSASNV
MAGGGQGNGGQPQLSQQQLSQQLTQQQLSPQMTQLLLQQQMSMHQGFVDAALAAAAAAVAAAPGGSASGVVPMTIGSGAAGLPGVAS
SVGSAVPPAGTEGDAAGALSTAGSAVPAEKASKKGGKISRLFKMGSSKSSK*

>Vop5_Vocar.0044s0018.1

MGGGGKASTLPAHLQDRIDAIETNQRDTDAQAFQKAVERRARVSFSVAIAGFGVLMFLLSAYLLCTSGIGDPELTAEFHSKTDPNCYTWPMV
AFSTAFGLNFITLLFERESAKFQLALLACYINFLAGFNDYLSWRGYSPIVRDSWGQGFQLLRTV**M**WLL**T**TPAMVY**L**LSIISDFSRIKVYSVMLAD
VLMISFGILAFLAFNKYLSILLYLVAWVFFSYVVYSMWTMFHASIAEARHDSSRVSLEVLRLFAVGLWFTFPGIWIIVK**L**GLVDVHT**E**EWMWCA
C**D**FLG**K**VMFSSSLLHGNFLTIEQRRLIAMRIVEEGNRYALKTITTIKTSGARLLNLINDILDAASMRKGKLTIKHEKVNLKRVVDDVIDLCQPLA
KRGVKLVNDLRENVPFVLGDTGRIIQVFHNLIGNSCKFTHNGFISIAASVKDDEVEVAVSDTGIGIPEDKFDQIFLAFEQVDMSVTRKYGGTGL
GLNLVKQLVEAHGGRISVKSKEGQGTTFFFTLKIHSSAEHPNEGQPQGTPTEAVAGPAEHSQLAAIRGTAAAPGGGGGHGHGPPRRAPSRRG
SFTDKMLGAKKTPSEAGKSSLAPGGQNAGAHGQPGGAARAAAAAASAAASPQSGEGHGGPAGGAESDLSYARFVRHMMPIRVSTRPTYLP
DEALFQLLAYVTCVALHDDSHDNGKLTKPNCQRAVGRPHARTHAHTRREKTLFVFATTPPPTPTPSPTSPARFHSPPGGIPGGSDAAAAATA
ATGVPGSGAGVVRRVSRETGGPGGGGAPAAGRSVTAGPQANAGFLVDIASVSPEAKDSGSFVSAGVAMPAIASANGGRPAAAGGADGGGG
GGGGGDAAAAAAAAAEIAKLKKDYESLEKQLEEVSNEAARLQDLVDDLEEQLIAKNGATEEASAQAAAASAQATAAAAQAAAAAAELA
ALRTQVSELECQLEEAARERGGLEAALAEMEQRLVATHAALASANHEAADRARSRPSALPEPRIETASMASGSEAGFGALGGGGGTAGGAV
GATGSSGMSLSTLLGGGGVLAHRAAMPLGMSMRLPSLASLRAHGGDMRSVLEELGLATLAARFEAEDISPGLLPYLDDTALRELGATSVGAR
LKLRIAAQALFLG*

>Vop6_Vocar.0009s0380.1

MRKRTGLPLPRQRENAPAQNSVNSTLGEDNGDPEAFRGWVKTWTWTLTGFCFYMTASWVVDACLPANPVASVELFKQVPLLVYQMSSTAF
FTALVLNLTSLLFEDNAPKRQLALLSCAIKGAACHTDMLLVTGRARVVFDAFGAIVIPQRYV**Q**WMV**T**TPTMVY**I**LSKISDFTPQQTATAICMD
VVMVLSGLMANFAPGPYLNWLMFSVSMLSFVGVLYMMGRMVFSAVKEHSSPSSRRSLLFIYMCTLLIWSMF**PVAWVLHLLNSSSPYGEYLNI
FANFMAKVLFSSSIMYGNYMTIAQ**RRLLAQQAAENAHRVRMIQELRDAVTRKDQFMSLMSHELRTPLNGIIQLSDALVRGAGGEMNPKG
QHFVKTIKNSSNHLLNIINDILDVAALKEGKLTIKHELCSLAKAVEHVVDIVAPLARKDVAIERSVDPHTPLIIADFSRVIQILYNLAGNALKFT
HRGRVCVRVSPSADGSSVTLQVADTGIGIPPERISGIWGAFEQVDMSVTRKYGGTGLGLNIVKQLVEAHEGKIEVQSAEGRGTTFTVTLPVLQP
CTRQSLELQVMESLSKCGHASARAAVSRRRSNRLASTSGRKPSLEETASKLARGTTRHASGLTSPSRRDAADGNVSGSNSQEKEDVLYRRRTQ
ELERENLMGELQRRAEHKRSMEEAHRVSRQLQLSEPERQAERDSRSLERLDLQIAARPPPSLPAPAAAAGGSCLRNSSETGVKSSPGSITAAGS
LGSFQAWRGEASEVAHRAVTGHSSRTSGEAREALATRGSVGGGGGGGGCSGSTAVSTGGNSLADVYEHLLLARGSTDSHSGHGLVRKLSATS
VGGGSKSNTVREALRMSTYGRTPGGGGCGGGGGGGGGGMHSHSGPVPGGTSHLLLKALDSDLYRGARESHDDGSSVGADSESDGLRAAGR
RSQKGPGPVSRSGKIMPASASLNSTNALVTASSLSPPPGLTLDKLPYSDMYGTVQILSVDDEEVNQIVLEEILTSTGYHFARCMDGLEALDWLC
ASETMPDLILLDCMMPNMSGHEFCATLRKVIPGNVLPVIMVSAKSDEDNIVEGLRSGSNDFVRKPYRREELLARIETQLRLKSDSWWLAELVN
NVDGRETESMKLLKNILPESIIARMQQGQKFVADSHPHVVILFSDIVGFTSLSSKLPTAEVFLMLSNMFTAFDKLTDRFSVYKVETIGDAYMVA
AGHDEDEDKARKGSPLTRVLGFAKAMLDVVRNITAPNGERMRIRIGVHCGPAFAGVIGMKCPRYCFLGDTVNTASRMESTGFPMCIHVSED
VYQHHPNMEGEFVEVGEREVKGKGRMRTYLVRVGAWEQALRDYAPRQQTAAASTSASASAAGQQQQQQHQRPAQATTMQQQQQQLQQ
QQQLSCQQPEREGLHGGAAEGAKSTGVSSGGLGGGELHPQPQLSQLSHRASFGAVSPGGSRSAGQVMLCPSSSSAAAAAAAAAAAANDGN
HNNGRFVHLLSTVAEEVSVPPSPLLLPDATAAVTDGNGVGFGLVRPQLTGSAMGVRSPLSRPELSAPRLPPRTTFGSEAYYCDETSDDGANSTL
NGMIMASGGALRSCSGSLTGLLPASPFLLQTAPATGAGAGARRAVSDASGYGNTGDGGDSGRQPGHASVLDVAAEFGLGVGGAAATAPAV
AHLREDGLAFAPATAAGGAGRVGSVTGGAVIPATGYLEQQITRLGTQLAAETSKRQRLQADLDEERRRSASALQHVTLLQQQAAALATAAA
FTSSATSSTAGRQDSTDRNGAVATSYGSCSASTKHPCNNLPSGGPSAKNGTKHGGNSVRQQKQLEQQLRDQEQQQQQQQPISQCDSSYAPD
AEIVILGPADDDADIGDGSGGSGGGSGGAAGTAGPDSDEGPALEQQRVPSRFPLHLPVAASSGSVEGGASPRAGLDPRVTTMQGTGNSSNLM
RDSPLVLTDIEWHSNGLRRPDDATAAAETGSPEAAVAVGRRETGGGVHVHATDARGGGTGGALVSSPVSPPLHPLHSPPAGLLAARPASDST
TNDTTTDQKLFYDSGARVNAAVEPFHTSLSHGLKAAVIVRAGTAGGTGGCHVVQSGWLMPRYSLDVLFEELGLLPYLGAFQEEAIRLDQLLG
MDTGQLERLGLRPLGYCLRLREAVLDLARRLLRACEEAARLPQPLALTDAAPVLGAPHAAAAEAIPSSINSGGGGGGGGGDGGGGGGGGG
GAAMRSG*

>Vop7_Vocar.0001s0831.1

MESDVLGHQSRVVLKTVSRMPRTFISQLYAVDMAVTGWVSFGSLSYAACAIYLASQNVDRSCAAGIGEQVASVEWTFQVQTAAFGAVLLLN
LLNLGMRQHLLPFKCNLLLLFINGVAFVTDLLLWRGATMVMFSSSGRPFAPLRYV**Q**WCH**S**TPTMIY**M**LALLADCGGCQLAVPLLSDVLMVA
SGLAACYTRGFLKDAMTI**VSFGTFIVVLYYVYGMFHKTLSSDVSPEQRSTLRTVLALLLALWSAFPVVWLAADWHLMGPQMEAICWGV
CDYLAKVVFSSQLWQSNLTE**VQLRRDRALEAWEASNRVEAVTRLTALLKQRDDLLSTLSHELRTPLAGIVALSESLCQEVSAALPSSAQNLA
TVRATATCMLNIVTSTLDSFAARRVGEPGGAPYVGDDGSGPVTPSVPLVALDLRPLVETVTTVLRPLAHDGVAIVSDLPSVLPLVAADSTRLSQ
VLYNLVGNAMRFTTHGEVRVAARMVLPEGAETTSGRPGDGGAAAATAATADLAPTTSNTSANGGGGGIWRYGRPRLSQSSVSSWSSVASFP
AGTQVEVSVEDTGIGIAQDLLDELFLPYRTGHETQVGGASGESSSPNGLAPTSTGRVSRSIGGTGLGLYLVRLALRAQGSDITAESAPGIGSVFRF
RLPLAQQSQQQHPGHHHHHHHYHTHHHVNGTAQPSSPPQPLTGPHHQQQQVVLVEEVSNQTHQQQQSHADYESGSHAQLSGSGFDCGE
ARFSYPVSAVTTASDGSGAERVSAAPSSPKPTVPSSPKPWVACATARLVKLSAGAAAAGSRTGDGDGESGAAIASAGPQHPPTSAFLLGESPG
PLHSRPLRMSLDEQRRNLLFPQHKQPSSASASAGSRCKASCTAECCGREPPAQGAAVLGVASKAAASTDILSAVADAGLGVAVPQVTTVPPL
RLVTPTLTPAPTPTAAMTRNSTGNVMSSLTPFDVAHAMEEGRQLARSGLRPNFRHRNNGTLQVLSVDDDPINQLVAGQMLTSQSWKVVKC
MNGPEALAHLQLAPKPGTVAAAAAAASASVSEQPVMGASASAAASLAAAAAAAQPLLLPAVLPDCVLLDVMMPGMSGFEVCRKMREHFS
SAQLPIIIVSAKGDSAAVDEAFESGADDYMTKPYKRTEMVSRIKAQIRTRDSILDAAARAAAAAAMLPAQPAQELSPPAPITSGSTTTAWTGLG
TGGTPRACCSSAAARMLPACSGAASEVPSPSPSPLHQVAHQGPHLDARPTSVDVFLLQQPTPNVIKMRMGASRGGLDGSWGRVGLYKRDG
MLSRTGLVEGTVTPWDAGLREGPLKDPVTAPAIVLGSPMIDLQPVEGSLTGAAGVSASAGAPGSSSDTIHKQVVEPLAPAAGLAEAAAAEVG

VAIGMSKPPEALPEATMVEPAKELASTANTPGLCLAGGQSPAATSGTGRCSSDAGSVTAAGIVAALVDVATASGASEGAVAEGAASSRGDG
GSSSGCRGCGDGGEPSGAAALMREVLTEVRRSRVVEADIERLTSRVAALRTACCTLAAERDGWRRQARDLRALLAAGLGGAIAVPAVSMPV
QAKARNSAQPHLALAEALAAAHGSRSSADSLGITGGGAPNGGAVAAQLEDLKKEAALLRQQLSMALASVGGNGSIALGGATDTGGSRNFG
LPSSCFPAVRPQRGLLGAPRTSDSPHSAAITRVGATGDQDRSGGGGGNDGAPPWGVSVFPNTAYVPPPDIFLTTEASGLSAGSGPGGGACEGC
ASGEAGAGGCHAAGAAARHGKRALTRAASSGVAGAPGLVSRAGLAAAAEIAIAKHANGGGGGGAAAAGGSVGKEDNAAAPVRASGDG
TSDAAVALALPQLPKGDTAQVLGELRGSSVASEGGRRATASTSAGDEMCRVSSTARAPVVQASSSRGSRSKGGGGGVAGTSGILRLLCSKGAA
PTGSSPNVSPHRRNPHHQQQQQ*

>Vop8_Vocar.0069s0008.1

MSCVPFPSILVHMWRMVSSAVDETMDPTSKRAIKFIRLFSITTWNLFPFVYFAAI**D**GALSLEI**S**EPLWAAL**D**WLT**K**MVYSSSLMEANFFTIAQRR
EFAVRAIEEANRLATIQQLSTAIERKDDFLSAMSHELRTPLNGIIGLSESLMAGACGQLPEKAVKTISTVKLSGKRLLQLINDILDAAKMKQGTL
VIKHEKVDIKRLVTDVLDLSLPLVRKGVRLVNNVGNVPKIVGDNGRIVQILYNLVGNAAKFTRQGVISITAGVSEDNIKVYVTVSDTGVGIPKE
KVPKIFGAFEQADMSTTRRYGGTGLGLHLVKELVKAHGGEITVESEVGVGSSFTIWLPISQDGVLTSVDDSEMKEPYALDLNEPLRRISEESEEG
GYHRNHVMGSRNTSEGDSDGDDGGGGGGGGGGKRRSSSATAAGGGGGGGDRSDSGGQEGVSGLGGGGSGSRRRLRTSAGGGAGGGSGT
AGMENLITVTKTGFKVMKPFYREKYGGCMVLSVDDDPINQMVVENLLSPEGYKVEQAMSGSEALDFLKKTATLPDVILLDIMMPDMSGYEV
CQEIRRRYSAVSIPIIMVSAKGHPEHVMKGLEAGSVDYVKKPFHRQELLSRIRAQVRNREIFEAEMESRKVTEMLKRILPMSVIQRLQQGQSMIA
DAHDEVTVLFADVAGWLSSQSLSSRSTSDVVLVLNEMYGTFEKLLLKYQVFRVEHTSESYLVVSGHDGTSDHIKRILSMAVDMLSAVQQLTFP
GGQQINIRIGVHTGPAYAGVVGIDNPRYCVFGDTVSVANALEAKGFDNTIHCSNAVYGAVRSKERDRGNVMFVPGRKSMTTGPTVMDTFIY
KAGSWETALKQLEDEEREKEAAEREAKLVEEGAAATRREGQEALAAQERARAEALAAQIEALEAERRSTEARVEMEAAEKRRVQERLAAEA
AAKAAAQQRLEAERVAAKAARAEAEALRNRAEAVEAERLRLKPFPIFLGRKSMAGVVTQKVYLLAIYTPLRMQSQPCQVLLFPASCQTDTD
TSTLNSNPTICVRNLNRTRVPSTQGYATALRPERRSGVEGSGRGGGGGAGRSGGAGKGAGRSGGAGKGAGRSGGAGKGAGRSGGAGKGAG
RSGGASGRIVGGSSRGGSGGGDGGRPWLRAGRGRHSGASCSRLHQLLIACS*

>MspRh1_62803 *Micromonas species*

MTTDAPGSESLPRVKAKHVFLLFALFAFVNTSLRRVFSPKDGLADSISLPDGSSSDGIERDHAFAAMMLFGAAFMFNMNAAMWSLHVTKTSL
NLQVAYISAVAAWTHYEMWLGKDWYVSYMGSRFMGGNAVAFSGLRQL**E**WCF**T**TPILLT**I**VQNIHAYAFAAAGTSDKGKRNPATYQPVNR
AVLIAADECMLLCGLVLPLASGVERAVFLTAAVACFVFIIYHSVHALLDVMTFSELSHMDSARLTAIVALKVIAWSGYPITYFATE**F**GYIDCKT
QHDWYLYN**D**VIT**K**FSYTLIISAGSLRFIEVLEERRKAF**AIQ**MSRVQRAFFFNITHELRTPLNSIIGFNTLAMESGELTEFTESFIKASLTSAEALLGLI
NQILDFAKFEGAKDKSGGSNATIELSDDVFTLRQLVEQVMDISQKASSRGVDLIFSITSPEHFNTKLVGDFFRLRQCCVNLVDNAIKYSSNVNG
RTALVEFSMGIAPGAKPDSLAITFAVEDNGVGIKLEKQHTLFVPFCQPADHKTAKEKGTGLGLVITKAIIECMGGDIDFESVEGTGTKFFFTLDF
ERAREASIDGGDDADFVEDAAQLEALPPKARVVFHPAMNESTRKHVTCIMKCFGAKPGVNYVTVPGEDTLKDKIRQASLLGVPVVLTDVINL
NSSLDFIKATPRAGIIIFGLPYQLMELHKNISDMRNVQTVLKPVKPSDLLKAIAKLTHQGATREIDFLNDTTDSEDGAVQKRLEHAEIKAAENA
LIKVEPPDVAEGTGELKGMTVLLVEDNIMNQQMAKFSILKCGADLEIAMHGQEAVDMVTARFDQGKPGYDCILMDMMMPIMDGATATTEI
RALEQRRRLTPQTIVGLSANVGPEYTARVKAAGMDGSMSKPFYPATLRNTLLNVKKGTYVGFGSSRLSIDRIHTTIEEKPGN*

>MspRh2_61324_Micromonas species

MATTMSRPRVSSGDEIQGSTLLALFAALVALNALVAPHLAGCPIGDGGSGAKGTGGTCAATDASIGGSRDVAVFAAAMFVAATAFALVNA
AMTASPDRSRHFQSAAYVNACAAFAHLQMVRGENWHVSSADGFSISGLRLL**E**WLF**G**TPVMIV**L**AKQLYALSREPPVDYVSAAVKLFERDRG
ADGVYIPPPSIRLVQADVLMLVCGCFIANLAPAPPAVRNGALFVGVATFGYVISHLSRCLAGSAVYSAIAGVRKDAKFGYALAVFNVVSWCV
FPTAYFLHV**S**GRIDAAR**L**DLGYAIG**D**AFA**K**FSCTMLYVTGCGRIMDLAEDEAAQRTDAQVAEQREYIRAVCAELKAPLDAIVGYGQMSADY
DENGEKTPSQVRRFQRAILTSAESMRELVSQAAQYSKVHGEIHARGRMMTGKEGEDVGLPRVAWRKEGGDSRAFPLPPMLPTPGRRFSRVSR
ESFDGDDETDPPSSVFTLGKLQDLVVKAANIPVGFELTCEISPPAATHTEHFRANTRLLRQALATLCETAAAQPSVVRRAGGVEAARARGAPP
CVRLSASLGATRKMEIAPVIDPNDESRDESLETSDLPEWTDATFTASLILDRRVGSHPSDASNSGNPHSLGVGVTPEELAKRRRLDLSTTVAKTV

VTAMGGSLIRRQSDSMSGDASGDQFSFTVPLLRTSRDESECDFVPVMPLPPGRTVGAVLLRARGAVGAGAMSANHVEHVASSLISYGAKVDK
CEHESDAASQLSEFASTDDVYPVLVMAADDEGDDENSWRHERLLAAAGARGSVVLFSSDASDDVGSDPKPSRHRRYANDSNVVVVARNAT
GEEVHEALCGVLAHVAANDANPTAAEPSNQATPSEDVSNPTNDEERRREKFDHSASGAWFKELGESEHLTRANSEHRKSRASHDADEDPY
AFHGSPSPQKRLVDESSQKNDAIPERTVAKPEPASTASKVKHGFKDDELDGLDVLVVEDNHFQLRIVKATLQRSGVTLDVALHGAEAVAHVR
RRVDAGGKLYDLILMDSMMPVMDGATATREIRAMESAYRSSKTGDELRDAGNAQPMIIVGLSAEGGPGYEQESRDAGMDGTLGKPCRPET
MRKTLKEVHRGEWKRGTFQSASKRASLHF

>MpuRh1_jgi|MicpuC3v2|8757|wlab.149627.1 in phycocosm or 70932 in phytozome
MGNAAVASTEPVSVARTQVMPTVSPWNLAGMATAFVLLNRAIQRVIPGISVGVPNADGSVESVDESHARMAFVVFTAALFFNVNAAAFSIH
DSKRKLNLQVAYISTIAAYTHYEMAEGRDWYVNTFGPSGEVVSGLRQL**E**WAC**T**TPILLL**L**VQNLHAFVFAALPRAMGPKKTSLTTTRDGTTK
AANAIAPYVPVNRIALILADELMILCGLVAPLTRGLERFAFLSVAMACFGFIIYHSLFALVDITRRADGLSVADAGRVKCIAALKMIAWTVYPA
VYFAVL**F**GVMTPAR**Q**HEIYLYN**D**VLT**K**FSYSLVISAGSMRFVEMVDQKRTQLTAHMAGMQRTFFFNITHELRTPLNSIIGFNTLAMESGELTEF
TESFIKASLTSAEALLSLINQILDVAKFEGAKDAGSSDGPAIELSEDVFTLRQLIEQVTDISQKASSSGVDLVIHIEHPEHFNTKFVGDFFRLRQCC
VNLVDNAIKYSSNVQGRSSLVEFTMSICRARGQRAGSARGERGGGDSRGQRDQQSEKGDESIFDDVRDVSGITFSVKDNGVGIPAAKQHNLF
VPFSQPADHKAAKEKGTGLGLVITKNIIECMGGEIEFESKEDEGTKFFFTVDFKHARLSAVDYEEEFEGATDLGFGSAVSGGGLDTEHEQSLPA
NARVVLHPGMSASSRKHVVNILKCFGGRAGVNYVSVPSADKLAGKIEQASGLGIPIVLADIEDTEKTLNLVKQTPKKAGVVIFGLPYQLIDFHR
RTSDLRNVQTVLRPVKPSDFLRAAWKLANVNALGEPDWMHDTGGECDDGVAERLRAAEANADHNAGRAAADSGASASPGDEGDGATA
AAASSASAEDLKHELLAGMRVLLVEDNVMNQQMAKFSIIRSGAELEIAQHGKEAVDMVSSRLEQKLPNYDCVLMDMMMPVMDGATATVL
IRELEKKHGAEKPHAIVGLSANVGPEYTDKVKKAGMNGSLSKPFYPATLRNVLEQVYKGTYAGFSGKGGKDAAAADIQSKPGN*

>MpuRh2 micromonas pusilla (40573)
MRAGSPRGVAAAATAPAHPARADELSGRALLLLFLAFLAADACLTPVFLSSSDAAPRRDDAPSSSATASATSFRRDVAVFGTALFTAATTLSV
VSALWATHPARRRHFWSATYVNAVAGVAHLQMMRGGEWWLVGDARTRGDLPSGLRLL**S**WFF**S**TPVMIV**L**MRQFHGLARKPPLSSLTTVS
KSAFKGGKKGGNRAAADGGGAAKAKTRASSSSYAPPPASHLAWGTVFMLACGCGGVIAPGMPPPIARVLVVLGGGA**FAWVLTQMVRILL**
GAAEGSAFDSVRRDRGRIRVLVFINVCAWCVFPLVFLARRAGLIDDDEAHLGFSAGDGCAKFSLSALYLVGCGRILDLAEEDVRRRVQE
GMADQLAFYHSLSFELRQPLSAVIGFGQLALEYPGPEKVPSAVRDFQRAILASAESMRSLVVQASEYSRLEAEIRNLGILKRPAIDRDENAEGDG
EVMGFSPATLLDDVMTSATTALTLGVEVVVETRPKTLLRAAPSTAKTTFVGNVDALRDCLVTLTQGACACRESKEGDCGGAVRCVKLLMEL
GDATSDEDHFHGATYSEATFTAFVPVNGVPSSSSKGPGASAPSSSSPSSPSSSRRVVSCRHLDEMGMDAAKNKMLGLSMTVARAVVEAMGGD
LSYALHPEDASEPDCFSISVPLRRVLYTGPHTTASAHSPPPGDDDSFDFDLLPALPRPRGSKTIAMTHPSLSRHFAAYVSDVARGSGVVVATAIE
EAGGGVVAPDDESDPDPGATLAAAWRRLGADPDAFPVLIAPADAFGGGEGAGGGDAGVGVAAAAREFRDLVCEGGSRASGVLVCATAAA
TADARAALRERDAVSVVRRPCTTRDLHAALLACVNGALARARVARQSSLGQSGGGGGGGGGGRRDSVDGGGRRASIDIEEFASLSTSRYMG
AGSGKEGRLEKGEVPATASAASASSPLHSDDEWATPVAAAEKSKEKSPPPSKESSGSFSEIRPKPASKATSTLKDDELDGLDVLVVEDNHFQLRI
IRATLQNSGVSLDVALHGEEAVDRVRGRIDRGEKLYDLVLMDSQMPIMDGATASREIRALEKAHREGKNAAAAAVLNPETMIIVGLSAEAG
HEYEREARAAGMDGALGKPCRPETMRKTLKEVHAGRWKRGSFNTAAKRAVSHF

>**GtRh1**_XP_005821993.1_Guillardia theta (146834)
MSTTQNSTTTVLDVVINQNISSNTPSGFQNLTFSDLIGEYNSTTSTNYLGIQYADFNPYTLWYICMALFLAAASIILALTFSIESFQRNCLLYWIFS
LQFLITASMYLFMSLNIGLLYEVYDVKVEPGIILQPGVMLHRPTNPPVYWMRNV**F**WTF**S**TSIDLF**I**LTVVVDSSKNISKQKKYKTLINWEYKTYL
FMINAALHTCGVIGTIVPASQISKVCKWTYWVFGLGLFVMLMNQLASSQCPLMKTAERNMVLQSYKKLVRVVIITWSLYPALWAIGD**G**SGTI
AWNL**K**EVMYAVI**D**FIS**K**FSFIAMFLHCTNSPSCHWNVSLASFASWFRLCLGDDGGLKVLCMQMDAMQPLACFAKAIKLKRHYGWRRVAP
MLYRMKKFPQTLVFPDLTRFDPDDINAVNGIHGIFLEKNEDTGEFLINSHGKYGKARSVRTFEPWVIQYRTPPRPPAELKEYMECMALDAYYA
EPPRCMNVRQALKKQVSVNHAKVWKEWHIAAFDDARFLHPVLSDARNPPVPSPSWLLDQWEYEITKFKARGSRKTETTNLPGKDFGSIGILS

GKGRFWFCESKSYEIGCGLRLDTKWPILFYTKNGVVGHPAEVDVSHDVSKSHKRLVTGVSLWQPIFSGVNNVKADAFYHKKAGVMHGASLF
VDYRDVRYRPDAKVAACLDRFYQAFLADGFLARNMLMGFMLKRLSRETIDTYMGEARNMAVVVCMDVVPASEPCLNEYPDHEQLRRDRR
ERRYFSQRKRELGDLLSRIGEMSRRNPDKRKEHDKICEALRKRREAMDHVLKERKYRLEREQRERVDLQCEARELDNFRLEIEKKMMSSVLAD
KDWSAEMEPILFEKERAFERKLEQRTARLRKEFEERCTAESEALLKEYEGRSPVLFAEDDGDLRYDTMDTEEHTFFENENKWLFSTGLARMSA
FLDENGERIPEITCEGEVEDDYDESTFIF

>**GtRh2**_jgi|Guith1|148915|fgenesh2_pg.256_#_8 (148916)
MSTSSVAYLRTPVVQALDWIGFISLGGTAAVLAYRLMNFKPPNKDILYFFGYREKGMISLYVNLFAAVAYYARITSHLSGDVGAATNIVLYKYF
DYLI**T**CPLLVG**T**KSKRGETFDLLTTLNLPYKITYAVYVQITIFTGFMSANTPPPATFLWFAFGMLLFSYTWFNIISLVQVRFIQYFAKKGNTTQSR
RVSVASKAGFRNKNVRNPLQTALSTYFCIWMVYPVLWLLLK**T**KVIDQVT**E**HCINVVM**D**VLA**K**SMYGFALLRFQLLMDKASLEMSELKVTKS
DLMEDFNAEKKKMRELRRRQQAEMEGEELSDDEEEAHGNSRQNQIKGGNHVSGTSSPISQEQLGMLLSQMTTMQQMSSPRQMAPIGQPAS
PLQASMMPGGMPNSFMSAPPPRMIDENAGNAPANWMSAQQVQQSAAGGVGGAQPNNWRQAFDDLDQGRGMSRDV

>**GtRh3**_jgi|Guith1|148916|fgenesh2_pg.256_#_9 (148915)
MSTSSVAYLRTPVVQALDWVGFISLGGTAAVLAYRLMNFKPPNKDILYFFGYREKGMISLYVNLFAAVAYYARITSHLSGDVGAATNIILYKYF
DYLI**T**CPLLVG**T**KSKRGETFDLLTTLNLPYKITYAVYVQITIFTGFMSANTPPPATFLWFAFGMLLFSYTWFNIISLVQVRFIQYFAKKGNTTQSR
RVSVASKAGFRNKNVRNPLQTALSTYFCIWMVYPVLWLLLK**T**KVIDQVT**E**HCINVVM**D**VLA**K**SMYGFALLRFQLLMDKANLELSELKVTKS
DLMEDFNAEKKKMRELRRRQQAEMEAQEMSDDDEDVDDQAGESNQSQKSKAGKQSSGKNSPISQEQLGMLLSQMATMQQMSSPRQMSQ
PSQMMMSPMQTSMMPGGMPNSFMSAPPPRMIDENAGNAPANWMSAQPVQQSAAGGVGGAQPNNWRQAFDDLDQGRGMSRDV

>**GtRh4**_XP_005833164.1_Guillardia theta (107802)
MTGSVPGDSDDAVIMEKTKFVKMQISFLFVICMLMDIGGICFQISKKKRALNSLVFFINTVSFVVYLSACFGWIPAIYSLDGKRVSVERFF**Q**WMN
TTPCMIF**V**LSALGNTLQKYLIHDVKELVKCVLWDEAMIFAGLLHAYLGFSLLGWMFLLTACFCFYQVMQKLHGAILISISKVATVYEVVSLRV
LEIFTIVLWSLFPLVHVLYF**S**GTITFTQ**Y**DIAQSFV**D**LAT**K**AIYSVTLITGNFFLLDTVAELRLEQLQAEKDARNSKVVRSEMMDHAMQMAVIE
AETSARLSSRFLANISHELRTPLNSIIAFNSLLLESDDLSTTQTEYVTSSLTSAESLLSVINQVLDFAKLDSESKLFKTSGEEETPKALQPFFLEQVCD
NVCDMMSSRVAAREVDFAVKVSGHHGHEEGKKLMLVGDSFQLCQCLVNLCDNAVKFARKTGGEAHMRVSLQRSSSDWAVVQVEVQDN
GVGIARESLDLLFKPFSQISSHYSREHGGTGLGLAITHKIVTSMGGTITCCSDGLEKGSTFRSHLASPAPPPFVPAPPAPAPSSLTPAPISSALGVAL
TCNRMVVPFAIYKQEEVADELPAEEEKSHSSEDKLHRHDDKPLPHDLQLVLCMARGPSFRSVELFCRNHKMKPESFAYDGPEPSNMSMIRLV
SKIKRTLGSDSPPVFLVQIEVYKELLKKQMQLPSRLLIFGYIDSQFELIESQDDMATRLVPRPIKHSLLHSKIHSMLLITAAKTERSSSVASGDSEKIS
ELDDVSHPDEERQQEKKLRVLVVDDHFANQKVAVALLNKVLGKDAVIADIASDGVEALKLVEDNKDDPYNLILMDIQMPNMDGLEATRRV
RLLEKKDFDDASSSDNLRVGG

>**GtRh5**_XP_005818773.1_Guillardia theta (122016)
MEKERREQQVFENLQVEGYADAEFLDKRPSCDSRMDEGLPEPLRPLSPPHESRSEAPLNQQEDHQRVPAFFRQSRHFSQSFKLPSLKQATQVD
QKFMIRATMNDVPGLFLFALWLMVYVVFVYALRSYSSACANVQTDESEYYTMKKTMFVKILAVFLFSICILMDLSGMLFQISAKKRTLNSLVLF
INLVACTVYLSYVTGVLPVLHDLEGRQMFIERYF**Q**WMN**T**TPCMLF**V**LHALGSSMEKELVISFSSLFRVLISDELMILTGLVHAVVGNSMIGCVA
CFLSCCFFLDVLRGVHAFTNSSVARAATTYEIASLRSLEISTFLLWSVFPVVHLLHY**G**GFISTFQ**Y**DIAQTFV**D**ILT**K**SVYTVTLLTGNFCILDTVA
EVRIAQLQAEKETKHKNVIRTELMNQALQTAMLEAETSSRLSRHFLANISHELRTPLNSVVAFNSLLLESEDLSAAHKDYVRSSLTSADALLTVI
NQVLDYPKLEAKEDNEKEGKGGEEKEGSELMEEFSLLKLGEDLSDLIASCIKSDLDFAVDIQVSEADAELFLYGDAFHIRQCAMNLCDNAMKF
SPEQGGEVTLRMRLSDLDEASSVCLLHVEVEDNGIGIPEDKLNLLFKPFSQVSSQYTRLHQGTGLGLAITKKLVDSMHGSISCQSRGAEAAKSR
GGAAAGGTGTVFCITVPLHFTRQPVGVNSTLEIPGLQEGTVILATRKGPSSALIMRLCKERKVRVVDLRQDKGPVSKQLLVKLQAAVKAQGD

RPFCVVVDMLVYEQLSAMATELSCLKSFLILGGMTSQLCLLKKKRHDEASNVIPLPIKPSVLFSKIFSSFTEEKKSPALPSFPYAAKVAKETVSSG
NVPALEEAVKKEAEEDNRMRILVVEDQIANQKVAVALLQRVFGKDKVAVDIANNGEEGVAAASSGSYDLILMDIQMPIMDGLEASRKIREW
EKEKDRKKICIIAVTAHTSPAHVEECMQAGMDRHFSKPLSLSLMRSIVEDFDDWKGSRV

>**GtRh6**_jgi|Guith1|149064|fgenesh2_pg.274_#_4 (149064)

MPSTCSFSHDSQMEKERREQQVFENLQVEGYADAEFLDKRPSCDSRMDEGLPEPLRPLSPPHESRSEAPLNQQEDHQRVPAFFRQSRHFSQSF
KLPSLKQATQVDQKFMIRATMNDVPGLFLFALWLMVYVVFVYALRSYSSACANVQTDESEYYTMKKTMFVKILAVFLFSICILMDLSGMLFQI
SAKKRTLNSLVLFINLVACTVYLSYVTGVLPVLHDLEGRQMFIERYF**Q**WMN**T**TPCMLF**V**LHALGSSMEKELVISFSSLFRVLISDELMILTGLVH
AVVGNSMIGCVACFLSCCFFLDVLRGVHAFTNSSVARAATTYEIASLRSLEISTFLLWSVFPVVHLLHY**G**GFISTFQ**Y**DIAQTFV**D**IL**T**KSVYTVT
LLTGNFCILDTVAEVRIAQLQAEKETKHKNVIRTELMNQALQTAMLEAETSSRLSRHFLANISHELRTPLNSVVAFNSLLLESEDLSAAHKDYV
RSSLTSADALLTVINQVLDYPKLEAKEDNEKEGKGGEEKEGSELMEEFSLLKLGEDLSDLIASCIKSDLDFAVDIQVSEADAELFLYGDAFHIRQ
CAMNLCDNAMKFSPEQGGEVTLRMRLSDLDEASSVCLLHVEVEDNGIGIPEDKLNLLFKPFSQVSSQYTRLHQGTGLGLAITKKLVDSMHGSI
SCQSRGAEAAKSRGGAAAGGTGTVFCITVPLHFTRQPVGVNSTLEIPGLQEGTVILATRKGPSSALIMRLCKERKVRVVDLRQDKGPVSKQLLV
KLQAAVKAQGDRPFCVVVDMLVYEQLSAMATELSCLKSFLILGGMTSQLCLLKKKRHDEASNVIPLPIKPSVLFSKIFSSFTEEKKSPALPSFPYA
AKVAKETVSSGNVPALEEAVKKEAEEDNRMRILVVEDQIANQKVAVALLQRVFGKDKVAVDIANNGEEGVAAASSGSYDLILMDIQMPIMD
GLEASRKIREWEKEKDRKKICIIAVTAHTSPAHVEECMQAGMDRHFSKPLSLSLMRSIVEDFDDWKGSRV*

>**GtRh7**_jgi|Guith1|138313|fgenesh2_pg.31_#_17 (138313)

MVLFSVWIVFYSLLIFLVHWISSRMTGSVPGDSDDAVIMEKTKFVKMQISFLFVICMLMDIGGICFQISKKKRALNSLVFFINTVSFVVYLSACFG
WIPAIYSLDGKRVSVERFF**Q**WMN**T**TPCMIF**V**LSALGNTLQKYLIHDVKELVKCVLWDEAMIFAGLLHAYLGFSLLGWMFLLTACFCFYQVM
QKLHGAILISISKVATVYEVVSLRVLEIFTIVLWSLFPLVHVLYF**S**GTITFTQ**Y**DIAQSFV**D**LAT**K**AIYSVTLITGNFFLLDTVAELRLEQLQAEKDA
RNSKVVRSEMMDHAMQMAVIEAETSARLSSRFLANISHELRTPLNSIIAFNSLLLESDDLSTTQTEYVTSSLTSAESLLSVINQVLDFAKLDSESK
LFKTSGEEETPKALQPFFLEQVCDNVCDMMSSRVAAREVDFAVKVSGHHGHEEGKKLMLVGDSFQLCQCLVNLCDNAVKFARKTGGEAH
MRVSLQRSSSDWAVVQVEVQDNGVGIARESLDLLFKPFSQISSHYSREHGGTGLGLAITHKIVTSMGGTITCCSDGLEKGSTFRMVVPFAIYKQ
EEVADELPAEEEKSHSSEDKLHRHDDKPLPHDLQLVLCMARGPSFRSVELFCRNHKMKPESFAYDGPEPSNMSMIRLVSKIKRTLGSDSPPVFL
VQIEVYKELLKKQMQLPSRLLIFGYIDSQFELIESQDDMATRLVPRPIKHSLLHSKIHSMLLITAAKTERSSSVASGDSEKISELDDQEKKLRVLVV
DDHFANQKVAVALLNKVLGKDAVIADIASDGVEALKLVEDNKDDPYNLILMDIQMPNMDGLEATRRVRLLEKKDFDDASSSDNLRVGG*

>**GtRh8**_jgi|Guith1|145205|fgenesh2_pg.99_#_1 (145205)

MPGQTSRAEEPCCEIRGLKEERKMPTSGMPGREDSEKDLTSSSNRDLASVLQKGGIASPSTISRLIKMGSSLKREHLFQSDDLDEQTPQQLTLKE
KLLFWAIDAPGIVLFTIWCLVYISFTFACWWYAPFSTPSIKGMEPMPNVHVIMHQAKFVKVVLATCIFGICIIIDIAGVLFQVSSKKRQLNSLVLFI
NLVSFTVYLSTIYDLYPSMYDFQGNPIYVQFFA**Q**WMN**T**TPVMIL**V**LASLGSSMQDSLVQDWGIVIRAILWDEVMLTLGLTHTFLGPGLYGWIC
FGCAGCCFMTVMWYIHSISSISLSNAATTYEIMSLRGLEAITYMLWVLFPSLHILHH**L**GLLDTLQ**Y**EIARTVV**D**VVV**K**AIYSVALMSGNFCLLDT
VATLRVAQLRSERDSKQTAVVRAEVMNRALQMACVEAETSARLSRRFVANISHELRTPLNSVIAFNSLLLDAEELDPLHREYVKSSLTSAEALL
GIINQVLEYAKLEAREDSDTGKGKGDGAPAIELNKQPFLLTDICEDLCDILSARVNMRKVDFAVDVAKQKKFEMLPALVGDSFRLRQCLINLC
DNAIKFARDDHGKVILRIHVDSQEKDLAQLRIEVWDNGEGIPEEKQKLLFKPFSQVSSHLRNHNGTGLGLVITKRIVNAMGGNVECRSSVGG
GGTTFTVTVPLPIAKSEDVKREEATQDGLSGRVLVVLREGPGLEFVRGLCEEWGLQSSVISFEENPHGHARAIATSQRLLEALKAQDEAVLVM
DCQVYLHLIADSPSSLASRRLLLVGHLDDQSKLPPKSMAQFVLRPIKPSLFRSKLEAAFSLTPAPVETSVVKPQKPQTEAEEAAPTGEDRMQILIV
EDHPVNQKVAIAMLHKALGKDKVNIVLANNGQEGLDLATARGHAFDLILMDRTDPPYFIVAMTAHASQADVEECLSSGRRREKERRMGGG
GGTRVKGEGEGEEKDCEVVGVDGMAGMDRYASKPLNLNVMRDLMAEFLTWRSANPRVSPTA*

>**GtRh9**_jgi|Guith1|144198|fgenesh2_pg.84_#_1 (144198)

MPGNPSVVMSSHSASLHDENTSTSNSHEPSREKKDVAIASSFRPSTASEQNLRARLTKESAIAKAESSSFVNKSSKSSFIHSPDKGSRQGSFRIDTA
DKKWSLGMIRSEAPGVVLFIAWCFMYALVLSGARQLSAYCRQQSASGTRNEQVIMDKAKFVKVLAAFIFGICVVMDLAGMIFQVSAKKRPLN
SFALFINLVSFIVYVSTILDYFPLMFDFDGNPVQIQFYV**Q**WMN**T**TPIMIL**I**LACLGTSMQDVLVQDWGVVVNAILWDETMLVLGLVQTMLGSSI
IGWVCCVCSGCCMVVVFRRIHQISNASVNNAATTYEIASLRGIEWFTYMLWSLFPCVHVLYF**L**RRIDLVQ**Y**EIMRTFV**D**VVA**K**AVYTVALLTG
NFCILDTVATLRIKQLQAERDSKDKTVLRAEVMNRALQMACVEAETSARLSRRFVANISHELRTPLNSVVAFNSLLLDADDLNPVHREYVKSS
LTSAEALLGIINQVLEYARLESKADGSESGSSRQDLLPSSIELTEKPFFLADLCDELCDILTARVNLRKVDFAIELCTEYKGGSVPCLYGDSFRIRQ
CLINICDNAVKFAKDEGGQVVLRIELLEEAPDGSAFLSMEVWDNGEGIPQDQQDLLFKPFSQ

>GtRh10_jgi|Guith1|144226|fgenesh2_pg.84_#_29 (144226)

MEKDFPSVNGVAKSRVVHIASPSEAPLISSYRNKMSQNSFHTKTKSSSFISDSGSNDEKGSFSTRIKKAQKEMPGVALFIVWCFIYVGFVYGSWV
LSGYCKPKMGFSAQSDEVTLDKSKFVNVLAAFIFGVCIIMDISGMVFQVSAKKRQLNSLALFINMVAFVVYVSTVLDVFTPMYDTTGEPIAVQF
FM**Q**WMC**T**TPIMIL**V**LASLGTSMEENLVPDWPLVINAICWDELMLVLGLTHNIIGSCFIGWAVFCCAASCFVVVYYRIWQIIDRNVKQAATTYE
ITSLRGLQIYTYLLWLSFPTLHILHH**L**HVLDDTQ**F**SICRTFV**D**VLA**K**AIYTVALMTGNFCILDTVATLRLQQMETEKDSKERTVLRTEVMNRAL
QMACVEAETSARLSRKFIANISHELRTPLNSVVAFNSLLLEADDLNSIHREYVSSSLTSAEALLGIINQVLEYAKLEAKADGSDDGSQSSKHEMI
PSSIDFTEKPFHLGELSDELSDIMAARVSGKQVDFAVQLCLSCKLGTCPFLLGDSFRLRQCLINLCDNAVKFCRDEGGQEEAPADSAFVCLEVW
DNGEGIPLEQQDLLFKPFSQLPSKHMSNNGGTGLGLAITKRIVSAMCGTIECVSNTDGRGTTFRIVVPLPVCSGRDFELTFPKFMARDSLRGKV
RLVLPEGPTKTMMKEMLRFWGMDIDDIFTAASAMDLTLKQMERLKAELRDKKEKEAKEGRPSVLIIDSQLQNGLLDGWSKDELNSLLVVGY
LDEQGGGKYKVSCDLHKLVLRPVKMSALYSKLLVTVLPSSLLAC*

>**OtRh1**_jgi|Ostta4221_3|72114|OT_ostta17g01230T0.. >jgi|Ostta1115_2|46936|gw1.24.242.1

MAVDARSWRGFARGKVNGMGVRLSALLVLFLSFVVVTEWVRDREGERFSAFTPRLIRQQRAVDARTGERLMVSGDVNANASATGPDPFEDL
RTYVDHGVVRLEAEVCGSTSLMALTVFAVCASIGLNDIMWTPSRSRRSVSSTVLYITIVACYTHYLMVQGSDLLKQGVEGTYYSLLRYL**E**WMM
TTPVLLI**L**VFQLHALCVGDERAPYRTDMWCACIADEIMLITGWLTNELSGKVHGVCLTISMLCFTYVISRCVGLFRELLTSNLVAGDRMRFIILA
CAKTICWTSFPIVYMAKE**M**EFIDTSA**E**HEWFLSC**D**VLT**K**AVYCLLLSAGSIRVLDVTRDEELADIERLTKMQREFFFNITHELRMPLNSVIGFNT
LAVESGELTDVTNGFIKNSLTSAEALLGLINQVLDYAKFNRKGVEVSGGHGLELSNDNFTLEELIDQTLSICQRADYTGNLFVRIDPSLFPMTYI
CDFFRLRQCLVNLVNNALKFSNHLDRPGCVMIDVRGYLDESEAHLTFSVEDNGVGIPEERQSAVFVPFSQQSDYSALRVQGTGLGLTITKTIIEL
MGGTIHFTSKEDVGTKFNISLPMGYKSADKMRIAPKNLMALIGLRPGLQMRTLRNILACYQLNVRDMIEVGLVGQDREQVEHYIMHGHTSN
KHVVLCIEEEQYFQNEDWLKSIKPEPKIMIISKSSRMKDLPHVANVRSIMKPVAMMPTIEALEYFSREGEGECFVDETESQGVGSIMKSESDTAA
SKSATDLDGDVDISSMRVLLVEDNRMNQQVAIQSMKKCRVTVDVADNGLIATQMIEDTIHGVIAPYDVIFMDVMMPVMDGNEATRKIRQM
EKEAPETYKRNLIIALSANVGPEHTIAVTEAGCDGSLGKPFYPSTLRQLLYSIFMDEYEGFAAQETFFARPG

>**OtRh2_**OUS45390.1_Ostreococcus tauri (199419)

MGRMGARDGTLTKGARTQVPALVMRSVENARVAIDDGKLDTAIKAMKSTDALCSRVVAPPTVHGLCLRVLADAYVSKKAAEGAGGLAGG
AMTEGDGSGGGEAQSSGEGDDGVNEDYAELAKEALKRGIDMCKVHEGKAGMNAYMRHDLLGRLGDLYAALGEIYREEGNHKEAIRALRH
GLARFEKNNQNDFTAATYNRVAYCYMEQNVWDAALDTLQSAERAATGNDAESILLSTTHAYRAQCYRALDQIQPAREAFEKALTFAMAC
GNEPVVREAEEFLAETQAHSAAQTRATAMAVDARSWRGFARGKVNGMGVRLSALLVLFLSFVVVTEWVRDREGERFSAFTPRLIRQQRAVD
ARTGERLMVSGDVNANASATGPDPFEDLRTYVDHGVVRLEAEVCGSTSLMALTVFAVCASIGLNDIMWTPSRSRRSVSSTVLYITIVACYTHY
LMVQGSDLLKQGVEGTYYSLLRYL**E**WMM**T**TPVLLI**L**VFQLHALCVGDERAPYRTDMWCACIADEIMLITGWLTNELSGKVHGVCLTISMLCF
TYVISRCVGLFRELLTSNLVAGDRMRFIILACAKTICWTSFPIVYMAKE**M**EFIDTSA**E**HEWFLSC**D**VLT**K**AVYCLLLSAGSIRVLDVTRDEELADI
ERLTKMQREFFFNITHELRMPLNSVIGFNTLAVESGELTDVTNGFIKNSLTSAEALLGLINQVLDYAKFNRKGVEVSGGHGLELSNDNFTLEELI
DQTLSICQRADYTGNLFVRIDPSLFPMTYICDFFRLRQCLVNLVNNALKFSNHLDRPGCVMIDVRGYLDESEAHLTFSVEDNGVGIPEERQSAV

FVPFSQQSDYSALRVQGTGLGLTITKTIIELMGGTIHFTSKEDVGTKFNISLPMGYKSADKMRIAPKNLMALIGLRPGLQMRTLRNILACYQLNV
RDMIEVGLVGQDREQVEHYIMHGHTSNKHVVLCIEEEQYFQNEDWLKSIKPEPKIMIISKSSRMKDLPHVANVRSIMKPVAMMPTIEALEYFS
REGEGECFVDETESQGVGSIMKSESDTAASKSATDLDGDVDISSMRVLLVEDNRMNQQVAIQSMKKCRVTVDVADNGLIATQMIEDTIHGVI
APYDVIFMDVMMPVMDGNEATRKIRQMEKEAPETYKRNLIIALSANVGPEHTIAVTEAGCDGSLGKPFYPSTLRQLLYSIFMDEYEGFAAQET
FFARPG

>OlRh1_jgi|Ost9901_3|25667|estExt_fgenesh1_pg.C_Chr_160087 (25667)

MARSKSTPIDARRERRMSAGARDARTWRAFARGRVDGTGVRLSALTVLFLSFVVLTEWIRDREGEKFSAFTPRLIRSRHVLDVTTGERVMMTE
ERARGSGLNAGNASGAAAANLRTYMDHGVVRLEAEVCGSTSLMALTVFAVCASIGLNDIMWTASRQRRRVSSTVMYITIIACYTHYLMVQG
SDLLMKSIEGTYYSLLRYL**E**WIF**T**TPVLLI**L**VFQLHALVVGGAKSRFRTEMYAICVADEIMLITGMLVNEMNGTVRGMLLTVSIGCFMYVITTCI
QLFREVLANELTKGDRTRFVILAIAKTVCWTSFPLVFMAKE**L**NYIDTSA**E**HEWFLSC**D**VLT**K**AAYCLLLSAGNIRVLDAARDEEMADIERLTK
MQRDFFFNITHELRMPLNSVIGFNTLAVESGELTDVADGFIKNSLTSAEALLGLINQVLDYAKFNRKGAEVDQAHGLELTNDDFTIEELIDQTL
SISQRQDYTGNVFVRIDPSLFRMTYKSDFFRLRQCLVNVVNNALKFSSNLDRSGNVIIDVRGYLEEQEARLTFSVEDNGVGIPEEKQSAVFVPFS
QQSDYSALRVQGTGLGLTITKTIIELMGGVIDFTSREDVGTKFNITLPLGYKATEKQHTAPRDLKVLIGLRPGLQMRTVRNILACYQVNVQDML
EVGLEDAQDREQVEHYVRHSHKANRPAVLCIEEEQYFAHESWLNSIDPEPHILIISKSSRMKDLPHSACKRAIMKPIAMSAFIETLEYLAADAK
NGEVDENAMRTRGLGGRDESTSSLAENSMMRSERTSSDSKRAPKSSAEQPDIDISSMRVLLVEDNRLNQQVAIHSMKRCNVFVDVAENGLIA
THKVEDTIRGNSKPYDVIFMDMMMPVMDGNDATRRIREMEQSAPPGYKRNLIVALSANVGPEHTLAVTEAGCDGSLGKPFYPSTLRSLLYSIF
LDEYEGFEGETMNTASRPGH

>OlRh2_jgi|Ost9901_3|89413|ost_16_006_019 (89413)

MRGAAKLRSDRADVARPSSTSDLAARGARRSTNRDDPNGLGASTTARRASDDDARAMARSKSTPIDARRERRMSAGARDARTWRAFARGR
VDGTGVRLSALTVLFLSFVVLTEWIRDREGEKFSAFTPRLIRSRHVLDVTTGERVMMTEERARGSGLNAGNASGAAAANLRTYMDHGVVRLE
AEVCGSTSLMALTVFAVCASIGLNDIMWTASRQRRRVSSTVMYITIIACYTHYLMVQGSDLLMKSIEGTYYSLLRYL**E**WIF**T**TPVLLI**L**VFQLHA
LVVGGAKSRFRTEMYAICVADEIMLITGMLVNEMNGTVRGMLLTVSIGCFMYVITTCIQLFREVLANELTKGDRTRFVILAIAKTVCWTSFPLV
FMAKE**L**NYIDTSA**E**HEWFLSC**D**VLT**K**AAYCLLLSAGNIRVLDAARDEEMADIERLTKMQRDFFFNITHELRMPLNSVIGFNTLAVESGELTDV
ADGFIKNSLTSAEALLGLINQVLDYAKFNRKGAEVDQAHGLELTNDDFTIEELIDQTLSISQRQDYTGNVFVRIDPSLFRMTYKSDFFRLRQCLV
NVVNNALKFSSNLDRSGNVIIDVRGYLEEQEARLTFSVEDNGVGIPEEKQSAVFVPFSQQSDYSALRVQGTGLGLTITKTIIELMGGVIDFTSRED
VGTKFNITLPLGYKATEKQHTAPRDLKVLIGLRPGLQMRTVRNILACYQVNVQDMLEVGLEDAQDREQVEHYVRHSHKANRPAVLCIEEEQ
YFAHESWLNSIDPEPHILIISKSSRMKDLPHSACKRAIMKPIAMSAFIETLEYLAADAKNGEVDENAMRTRGLGGRDESTSSLAENSMMRSERT
SSDSKRAPKSSAEQPDIDISSMRVLLVEDNRLNQQVAIHSMKRCNVFVDVAENGLIATHKVEDTIRGNSKPYDVIFMDMMMPVMDGNDATR
RIREMEQSAPPGYKRNLIVALSANVGPEHTLAVTEAGCDGSLGKPFYPSTLRSLLYSIFLDEYEGFEGETMNTASRPGH

>OlRh3_jgi|Ost9901_3|28080|eugene.1600010136 (28080)

MSAGARDARTWRAFARGRVDGTGVRLSALTVLFLSFVVLTEWIRDREGEKFSAFTPRLIRSRHVLDVTTGERVMMTEERARGSGLNAGNASG
AAAANLRTYMDHGVVRLEAEVCGSTSLMALTVFAVCASIGLNDIMWTASRQRRRVSSTVMYITIIACYTHYLMVQGSDLLMKSIEGTYYSLLR
YL**E**WIF**T**TPVLLI**L**VFQLHALVVGGAKSRFRTEMYAICVADEIMLITGMLVNEMNGTVRGMLLTVSIGCFMYVITTCIQLFREVLANELTKGDR
TRFVILAIAKTVCWTSFPLVFMAKE**L**NYIDTSA**E**HEWFLSC**D**VLT**K**AAYCLLLSAGNIRVLDAARDEEMADIERLTKMQRDFFFNITHELRMPL
NSVIGFNTLAVESGELTDVADGFIKNSLTSAEALLGLINQVLDYAKFNRKGAEVDQAHGLELTNDDFTIEELIDQTLSISQRQDYTGNVFVRIDP
SLFRMTYKSDFFRLRQCLVNVVNNALKFSSNLDRSGNVIIDVRGYLEEQEARLTFSVEDNGVGIPEEKQSAVFVPFSQQSDYSALRVQGTGLGL
TITKTIIELMGGVIDFTSREDVGTKFNITLPLGYKATEKQHTAPRDLKVLIGLRPGLQMRTVRNILACYQVNVQDMLEVGLEDAQDREQVEHY
VRHSHKANRPAVLCIEEEQYFAHESWLNSIDPEPHILIISKSSRMKDLPHSACKRAIMKPIAMSAFIETLEYLAADAKNGEVDENAMRTRGLGG

RDESTSSLAENSMMRSERTSSDSKRAPKSSAEQPDIDISSMRVLLVEDNRLNQQVAIHSMKRCNVFVDVAENGLIATHKVEDTIRGNSKPYDVI
FMDMMMPVMDGNDATRRIREMEQSAPPGYKRNLIVALSANVGPEHTLAVTEAGCDGSLGKPFYPSTLRSLLYSIFLDEYEGFEGETMNTASR
PGH*

>OlRh4_jgi|Ost9901_3|47806|estExt_GenewiseEukaryote.C_Chr_160302 (47806)

MMTEERARGSGLNAGNASGAAAANLRTYMDHGVVRLEAEVCGSTSLMALTVFAVCASIGLNDIMWTASRQRRRVSSTVMYITIIACYTHYL
MVQGSDLLMKSIEGTYYSLLRYL**E**WIF**T**TPVLLI**L**VFQLHALVVGGAKSRFRTEMYAICVADEIMLITGMLVNEMNGTVRGMLLTVSIGCFMY
VITTCIQLFREVLANELTKGDRTRFVILAIAKTVCWTSFPLVFMAKE**L**NYIDTSA**E**HEWFLSC**D**VLT**K**AAYCLLLSAGNIRVLDAARDEEMADI
ERLTKMQRDFFFNITHELRMPLNSVIGFNTLAVESGELTDVADGFIKNSLTSAEALLGLINQVLDYAKFNRKGAEVDQAHGLELTNDDFTIEEL
IDQTLSISQRQDYTGNVFVRIDPSLFRMTYKSDFFRLRQCLVNVVNNALKFSSNLDRSGNVIIDVRGYLEEQEARLTFSVEDNGVGIPEEKQSAV
FVPFSQQSDYSALRVQGTGLGLTITKTIIELMGGVIDFTSREDVGTKFNITLPLGYKATEKQHTAPRDLKVLIGLRPGLQMRTVRNILACYQVNV
QDMLEVGLEDAQDREQVEHYVRHSHKANRPAVLCIEEEQYFAHESWLNSIDPEPHILIISKSSRMKDLPHSACKRAIMKPIAMSAFIETLEYLA
ADAKNGEVDENAMRTRGLGGRDESTSSLAENSMMRSERTSSDSKRAPKSSAEQPDIDISSMRVLLVEDNRLNQQVAIHSMKRCNVFVDVAE
NGLIATHKVEDTIRGNSKPYDVIFMDMMMPVMDGNDATRRIREMEQSAPPGYKRNLIVALSANVGPEHTLAVTEAGCDGSLGKPFYPSTLRS
LLYSIFLDEYEGFEGETMNTASRPGH*

>DsRh1_0121s00015.1_**Dunaliella salina**

MLREKANVVAPRPGDAKEAIRISTRVWLGVGTTSYLTCAFGLWSTFINGNSVAHEQNLARLHAEVPGQYLEWPMSAFAAVLVLNVLNLALR
QNTTKFRTNLLLLFINGVAFTTDLLINLDLTPVFQAANGRMFHPLRYL**Q**WSH**S**TPTLIY**M**IWLSSDPEEVEAPQLMYAVANDVVMILTGPAST
CSSGSMQL**LWGAVSFATFFAVLITVSKMFRSNIDNLEDSEARGIVRAVLFLMLGLWNLFPVVWLLAELRMISPAMEHLCWGLCDYAAKA**
VFVSQLWQSNIASVYERREIALRMWEESNRVLVIDKLQRLVDAKEQLLNVISHELRTPVLGIMALSGSLVKDVQQKVPINVNHLCMMKSSAA
FLNNLISASIETAKNKDQKPQAAHEAVYMYELVEEVCTLICPLVREGVTLTNAIPSELPPVVGDRTRLMQVLFNLVGNASRFTMHGHIHVRAR
ILDNYIEISVEDTGIGVAQEDIEAIFQPYLTLTSGVASSCGGTGLGLYLVKLALDGHNADVNVESEVGKGSAFH

>KnRh1_GAQ84541.1_Klebsormidium nitens (kfl00193_0190_v1.1)

MADEVPSTPPGGPPAAQEGEKEGPPSPVVPVPRGAVPQLLELLDSERSLRKPPRTWLIASSVSALVATFAVLVLLLRQWHPPAGRQAPFDPTR
HSTEVGMVAFGCAFILFTCSLICRTDKEKRTAAFLVCYINAIAFSTNMLLYYSATPVVLDFFGRPCFPVRYV**Q**WLH**T**TPTMIF**M**AAKISSFTSPQ
VVVAVLYDMLMLATGLTASLAPSWPVMAAAISCSCAAFLAVLVLVWNMFRSALREARDLASYRNLQFLQAHMVFTWNIFPLVWALSA**L**HV
ITPET**E**AYLYLAG**D**LSA**K**VVFSALLLEGSFLSIEEKRRAAAAVQEENSKNALIAELRQAVSQKDRFLASVSHELRTPLNGIIGLSDTVLQGSCGPV
TEVLLHTLGTIKKSGHRLQNLVNDILDVSSARSGPLVLRYDRVKLRPCVDHVLDLTTPSLKWSVKLHNDVPAGLPELQCDPARLIQVLHNIVG
NAAKFTDEGEIRVSARLRKNQTGQDVVAVTVKDTGIGIPAERIADIFSAFEQGSDAIVQQHGGTGLGLSLAETLVRAHHGSISVTSRLGEGSAF
TFVLPLVQPKREKEESAPEEPEPSRSRPPSAPSPQREADRRERASSSADSAASDGEAAPKFARSSLTKFQRESASGYTLKQQITGHCASEGFEDGA
GRLHRSFFDVNGQYEILSVDDDPVNQMVVEGMLRPAAYKITKKMDGMEALAYLERSDTTPDLILLDVMMPGLSGYEVCRKVRQMFPASALP
VIMVSAKSQEGDIVEGLKAGANDYLTKPFKRLEILARIETQLKIKKLWKVEM**EAAKSYQLLKKMLPES**IIHRLNSGQSLIADSHPEVTILFSDIV
GFTEIAGCSRTADLIFMLNDMFTGFDELVDKHGVYKVETIGDAYMVVAGHDGAADHAGRVLRMAMDMLALVHAIKQPNQRESVRIRIGVH
SGPAYAGVIGVKCPRYCFFGDTVNTASRMESNGYAQAVHCSPATHDLTKHLFDFATVPRRHIKGKGPMETYLLKHGEWELAHRALLADAA
AAPPLERAGTGWWLXMAVGGRVPEDKGKEDGEKSAEEKAPAQSETENRLSDSRIRASSEPVPPLRQEVDKRPPVQDPGTQPSSKLAADEPPG
VSPVADASARARFTSLRITPPGGPLHPASPRIRSPLSRSYSSPDLKVLINREAFQAAYPESSPSLEGMSPAPANGTPSPKPSVRSTSAGGSLGSTPVS
ESERRVSDGSPKLELHSTKFVQVGKTAWVLKRPPSISGAKGDPAAPIVGFGVAGKAAAPPAAKDTRMEHLKMMLGSDPGSYVRRLSDTSIISD
LDGPHEDG**GPPERGTA**PAGVPAADGSRTKAESERAGRPAETSLAKETPSAKDSEVKAHTTKPTAEKSPPSTDDVSRGAPKREEKRKEEQATE
KAPPRSLPVMVQRNLAAALEGPAAGGGGVLGASGSLRQYSRVSRTELVCLAASSCGFSDPHSRDVSNSGTPEGSKRPSFETPEGSKRSSLEERD
PPVGGPVADPAHDKGFSHLRPGQDDRAPPGVEQQVCGRVGEPGRKPRWSGRDPSPLKPRPDIAGIPLNLTRLEEEVKPLALPLADRSMANVE
EEFPVVTKPSLENGTGTEANGTLSNDQGVSSDDEQVAPLTAKKDGSRSPERSPRRYTDFAPAPLRVATSSAHAAHRRSVSQWLPERNSPPGAP

PPQPTGSPAQRTKAEALAQQPGQRRKTSVVSPGGSSQDRSPEHRAPERPVPDPLSSQTRAAAAAVAAARGIKVPAKPRLAEGVELDAATLERL
TEAITEQVLSHLGTPRGSTSSISSSRGSPSGQDRAQRSLSASPLRGAGWGPWRVPARGIPLDEASRDSSGKTSPGGSRSPRGRMAFTEFAAHDDG
WAREESRFASDPAYPGAAASSLPIQGRHVAGFPWQLELTRSYSEDGVAARYERDRSEARAPPGGWADVASDVSSGSWSSFESTYTGGAPGFD
RYRPEVTSAEGDDLQARGANGARLADASGNGHLAGGRDAPGEIARFLTGLGLGHYLDTLEQIVLPTLLTGIVRGAARTRSARETVLLFADVIL
PSRWQEEVTLAVLSRLTLQQLKEVGVHPLGARMRILEAFKARTPH

>KnRh2_GAQ88354.1_Klebsormidium nitens (kfl00421_0020_v1.1

MESPLRAEARCTQLTVFLMIFAAALVTALALFVRSLYLDASQYTTGPPPGDHTRAALVAYINGTALLTYVLLDRDYTPVLVDHFGRPHYPARF
L**Q**WML**T**TPIMIY**L**TASISGQSHRQIILNTFCDLFILMVGLNGLLSSSNLMSGVCLTAAGFAFVTLLRNVHRMTNDAVLETRDSGCHWSFHLLQI
YILTTWTAFPCVAFLSE**T**NLVSPIM**E**AHMFLAL**E**LAC**K**FVFSALLLEGKFLSLEENRRVAEVARDESNKSALIEQLQQAITEKNRFLASVSHELR
TPLNGIIGLSDAMLLNLDGRLQGSLGEVNEDMKKTLETIKQSGGRLKNLVNDILDVESLRQRKLTLHYEVVYLRHLVDHVMDLTSPMVKWN
VKLYNDVSKDIPEIDCNQARLIQILHNLVGNAAKFTDGGEIRVCARVRETDDGHEFMIVDVRDTGIGIPSHKLQTIFLAFEQVNETSMDQRGG
TGLGLFLVDSLVRAHGGTISVTSEIGHGSCF**TLKLPLKHVDVNKSKPGPAPRSRLVSLAVKTPDDGLNSTPKTASSKATSPKATSPKGTSPK**
AVTPPGARSPKNTSPKAASPAAASPMAASLKQKLTGSPGRMFVCSDGKGHVTLTPKTALAAIPDSDVTQDEKRAVTFEAGGAGIPKGD
GGSTPESGKKGSLSRCASTRLVGNLYVGEFELPKRAVSAPATAGQESSKIARKAGSLWPDLRSLKPVEVELSFKDQTEDHSSTAVHYPTKLRSH
YEVHGQYEILSVDDDRVNQMVVDGLLRPAGYKITKKMDGMEALAYLEGSEVIPDLIMLDVMMPGLDGYEVCKKVREMFPSSALPVIMVSAK
SQEGDIVKGLRAGSNDYLTKPFKRLEILARIEVQLKIKRLWRVEIEAKKSYQLLKKMLPDSIIERLSAGQSLIADSHKEVTILFSDIVGFTEISGSSAT
EDLILMLNEMFTAFDALVDKHGVYKVETIGDAYMVVAGHDGSPDHALRIVRMAMDMLAKVRDVKQPNHVDPVRIRVGIHTGPAYAGVIG
TKCPRYCFFGDTVNTASRMESNGFQMSIHTSMTTYERCQDSFEFAPVGRRIIKGKGPMETFLLKYGEYEAGLQALRAENRPTLARSDSSPDLLK
RHAKIDPASLPPSVSPLLKQSLEGPSRKTSRFKSEGPRREQDVEFAQDLPSGYVSDDVSCGGGPAPGRERKLDRTRTGPRKEQDLEYARDLPSGY
VSDDVSCGGGPAPGREKKLERTRTPPRRSLGSALLGITPLRRSGSFSGMTAQKEESAPAVNPDPVRTLRVDESSVNNIPVVAAQALNRSGDRPL
PEHVTSHVAVPRPSTDLGRVAAQQMPSGRETPAQEDGIKWRTGLGVQDGLSDPTGLFEILDGMGLGRYASVLEEAEVTVDILATMTPEQLKE
AGVQAIGARLRILEAFRAPKATGST

>GpRh2_KXZ46245.1_Gonium pectorale

MDSQALVFRGAALDIVPAAVYDRSTSIFLTALAIDLLNILFERQTIKLDCVLLPTFIKGMASTTNVLVRFYTPVVGLTNLGRPVVIQRYI**C**WMH**T**
TPSILM**L**LKMISTSITPREAAVAILFDEIMVVTGVIALVTTGWQRVFWSCITHLAMLPVVPYMHKAFTEAMEQVRTRPLQLTMLCIYVLNLILW
CTFAVTWDLAL**L**GWISVMT**E**EVLYVAC**D**FSA**K**VLFSSTLMLSSFKEFEARRESAMRVIESSSKAKLIAELQALLEQKERFMSSVSHELRTPLNGII
GISEGMLSGCCGVLPEGVRRQIYIIRTSGARLLALINDVMDAAALRQNKLVLKQEQVVLRHVVDDVLDLTRSLVDSEVQLVNLVPPRMLVVG
DTGRIVQILNNLLGNAAKFTRRGQIRVTARQVEGGRKVAVTVSDTGIGIPRNKLATIFLPFEQVDMSISRKYGGFGLGLNIVQELVKAHGGTIN
VSSIEGKGSTFTFTLPLARAMGRESLEEAKAAAKRSQQGLGIAITDASSSQVGGGSVSGSGAGPAGKDGTSGSVTASGISPRGAGGGDDSSTIVS
SSDASRRPGQKGSTAGGVPNPAEVEQYGELNYNAAELSALASSRPYAQSARESLPASYEGNRSSSAHSPSIQPAVSMRSGRSGGPPSAAPVPAR
SRGDSSISGAGGAAVGGTSDVPQLASDAKIVHGPRPFHYTMYKRFLLLSVDDDHVNQSVIKSLLSSTGYEVVAVPSGPEALRYVSTAPALPDLV
LLDCMMPEMDGYEVLQRLRAMTPHVHLPIIMVSAQTEEDHVVCGLDLGADDYVTKPFKRNELLARIRAQLVYGDWEQDEGALETLSSLAIG
EVGGASAGHASPGNGGSSMHILDSGNNMGDAAHAAAAAAAAAATAALSPGGLADSGRRNSLPGVGGSDQRLIVCIDDDDVNQVVLQGM
LTSQHYRYVRASTGSQGLSYVCGTNSGGIPPDLVLLDCSLPDMTGFDVCRCIRQMYNKQQVPIIMLSARHNESAVVEGLKCGANTYVTKPFRR
NELLARIRLHLRSREAHHGSTPDSSNEDSDPRAGSNAASALTDAGASTPGKSSNGGRALQRAHDPVVASAAAAVGPGSMDAVRVWPEAAV
LVVAVADYGGLCRMLLPAEMAELSGRMLAAFDRAVTAHGAVRVEASSGVMSAAMLPAAVAPGPGAAADPADAALGLRLATLHRVGRAL
IEAAATIGVPGTNTCLQLQQALALGPLMGHSTPPVFGGLPGQGGGAGSGMLYFGPVLAELLDLARRAPPMTIVTPDMEAQALRSSGATADIR
GPLFLEADDVGGPAAAALAARRLWLVEAHPLAHQYLPPQLLHAASNVLRAGGFFNRSGTDDMDKSSLAASTVAGPSPLGPRGGGQGGSTL
ASGLSGGAGGGAGSASHGHGAAGGSSLLGAGEHGGQAAASAASTSVGFNTSLGFGLYGGHNISRAASRAFSQTFGLDSDALGRMGQQLAA

SGGNTGTGSGNGASQGSGATTGVSPGGATGSARGGGGSTGSPLGGGQNMIDSSSGGAAGITFSLQAAAAAVGASTGGGSGPSGVAANADLY
SIGGGSSVLLSVPPSNPFAVPHAPSGPSFLYGTAATPGLRASG
GVPSAVGGGGSGSQVSPSEQDAMAEPHIRALRNELAVMRQQLEALQSGSASVRSEAHAGTSGGSHAQPPPTPQALLVQHLQQQQQQAAQQ
QQQPQHFLAPAVQAGPQHADSGALQSLASVGEQGLVGARGSEAGGGGLVAAASGSIGGESLGAGLAEKAQKKGSKLTRLFGKSSKSGAK

>GpRh3_KXZ47741.1 Gonium pectorale

MDPHVCLDPVPPLVYQMSSTAFFTALVLNLTSLMFEDNATKRQLALLSVAIKGAACHTDMLLVTGRATVVFDAFGAICIPQRYV**Q**WCV**T**TPT
MVY**I**LSKISDFTPSQTATAIAMDVTMVITGLLANFTPWPLNLLMFGASMLAFGGVLYMMGRMVYGAVKEHTSAASRRSLLFIYMCTLLIWCLF
PLAWLLHV**A**SPESAAGEYLNVFA**N**FMA**K**VLFSSSIMYGNYMTISQRRLLAQAEAENANRVRLVADLRDAVTRKDQFMSLMSHELRTPLNGII
QLSDAMVRGAGGEMNPKGQHFVRTIKNSSNHLLNIINDILDVAALKEGKLTIKHELCCLDKAVDHVVDIVAPLAKKDVAIERSVDPRTPLIIA
DFSRVIQILYNLTGNALKFTHRGRVAVRVTPAADGGAVTLVVSDTGIGIPKERIPSIWGAFEQVDMSVTRKYGGTGLGLNIVKQLVTAHDGNI
TVSTTVTVTSEEGVGTTFTVTLPVLQPSARRSLEVQVHDSLAKCGYQVPRAREKPRSRAVSGLEDTFTQLTRGITRKASGLLSPKQEPPGQSDSV
ADRADRADKERDERDVLYRRRTQELERENLMGDLQRRTEHKRSMDEAHLASRSLEMTGLERRAERDTTSLDRRGGGGGGGGGAAEPPAQL
PAAGPSDSSRLSASPTGLSGLLSARSSMVGGGGGSARQLAGGGGNSFWRSDAWEQQQQLNNNNNNNLRRSSIESQRGGGGGGGQPSGLVRKS
SNVSLSSRTGGPMGQPYGMPMRGFGRPSNAGAAANGGGTSLLKSALESDLYRLPYRDSWDGASSFGADSESEGARLSRRHHHVAAATKLAA
AGGSVAGGGGGGGGSFSMGTAAAATASPPGLTLDKLAYSDMYGTIQILSVDDEEVNQIVLEEILTSTGYGFVRCMDGLEALEWLCASETLPDLI
LLDCMMPNMSGHEFCATLRRVIPGNVLPVIMVSAKSDEENIVEGLRSGSNDFVRKPYQREELLARIETQLRLKKDSWWLAELVNNVDGRETE
SMKLLKAMLPESIIARMQQGQKFVADSHSHVVILFSDIVGFTSLSSKLPTAEVFLMLSNMFTAFDKLTDRFSVYKVETIGDAYMVAAGHDEDD
DKAAKGPPLSRVLGFARAMLDVVRNITAPNGERLRVRIVSSGGSEGGEGKGERG

>GpRh4_KXZ54193.1_Gonium pectorale

MIAFSTAFGLNFITLLFERESAKFQLALLACYINFLAGFSDYLSWKGISPIVRDSWGQGFQLLRTV**M**WLL**T**TPAMVY**L**LSIISDFSRIKVYVVMLA
DVLMIAFGILAFLAHNWILTILLYTIAWSLFTYVVYSMWSMFHASIAEARHDSSRISLEVLRLFAVGLWFTFPAIWIVVK**L**GLVDVRT**E**EWLWC
AS**D**FLG**K**VMFSSSLLHGNFLTIEQRRLIAMRIVEEGNRIQVIQELKDLVEQKERFMSSMSHELRTPLNGIIGLSDALLVGSCGDVNDQALKTITTI
KTSGARLLNLINDILDAASMRKGKLTIKHEKVNLKRVVDDVIDLCQPLAKRGVKLVNDLRENVPFVLGDTGRIIQVFHNLIGNSCKFTHSGHI
AISATVKDDEVEVAVTDTGIGIPEDKFDQIFMAFEQVDMSVTRKYGGTGLGLNLVKQLVEAHGGRIGVRSKENAGTTFFFTLKIHSEHSTEGQ
GGSPSDAAPSSVGEHSQLTAVRGGPGASTGHQHPAPRRAPSRRGSFTDKMLGGHKKTPSETGKSMLGAVVSGSQLGAVTAARAAMQGGGG
GGGGGGATSGSGMAQGAEERERHGASLDQQQQQQTSRKSREGAHPHGPASFTKGPQVSGPMSLNDAAALMKGALKRKSSFRTKGTKVRV
LSVDDDPVNQLVIQNLLAPVGYEILQAMDGQEALQVLTEEERLPDVILLDVMMPGMSGYEVCRKLREMYPLSCIPVIMISAKSKEEHIVEGLA
AGSNDYVVKPFGRQEILARIAAHLRFRDTVYQAGEIAGAIPGEVLPSRVLLRGGAGGALDLAPFLTGPARFTSLPPRVAKGI**EAG**TTSTTLQMF
DQLTLLEVSIPQLGDLLSSVPASELLVALAQLFHDLDTLLEQHGCFLLEGVDDSFIIVSGLDNLGDQVLHALGLARSLIAADTFTLGGRRLKM
ALSIGIHTGPAQGVLVGHNHPTLYFTGQLPAEVHMLAATCPANCVHVSARVVEAVGTEREHFVAAGHMNSGSTSTYLMKVGSWEAGGIVA
TSDNTARWGKGKLGVHEGINTEARRMRPIQLALLAMAQTNPGFLVDIASGGPEASQKHAGGAPSDPAAAGAGSSAAGGAAAGGDPAAAA
AEIARLTREYEALEKQLEEVSNEAARLQDLVDDLEEQLIAKNGCSAAATQQAEAAAAGAQALKTQVATLESQLAEAARERSGLEAALAEME
QRLVATHAALATANHEAADRARSRTPAPEPRVETASMASGSEAGFASAAAAAHGSGSMFGGMGGGGALAPRVAMPLGVGMRLPNIASLR
AAGGDMRAVLEELGLSSLAPRFEAEDISPGLLPYLDDGAMRELGATSVGARLKLRLAAQALFMGA

>GpRh5_KXZ55246.1_Gonium pectorale

MAVAKSPLGEVDRNRRYPSRLAAASFPKDYRDDYDDSDDAVDDSEDTGNDGDARRGRSVQRRAGIKARPDKAQKVRTRGARPLTRTTATV
SNGGSGPPKRRHSARLLKQHHDQDKDGGASDRGEEEEEEEVGEGAEVGGTSDKDRGSGDGGGASSDSGSSGQEDEDEEMGDEGAAAGPRQ

AGSKSEGEHADDEEDGGVWKGRYHLRGGAVNALRAIPAKRPRYNEDGGGGAKRRRREDEDDEEEDEGEEEEDEEDEDDDEEQGGKGGRV
RDRARERDRRSERDRRRERDRSRKGKGSAAPYTLRDRSRMVPMSVQEQERQKEELRRELQARKRFRDRARAHGGGKHGGAGSGGGAGHGG
GGGGGSGHRRSGGGGFGGWRGGVSDDDIPDIDGTSMGNPWRNLQAAAGQLPPWQQHGAPNTPLPVLGQQANGGAQVVPSSALHGLGA
GVAGTPFGGAAAAAAAGGGLPLAPWEQALLADAAALAGGGVKEKAGNAEINPVAVDPSVGFDQVGGLDSYVEALKEMVFLPLVYPELFT
RFNVQPPRGVLFYGPPGTGKTLVARALASHASRYGGRKVSFYMRKGADVLSKWVGEAERQLRLLFEEAQKNAPAIIFFDEIDGLAPVRSSRQD
QIHNSIVSTLLALMDGLDSRGQVVVIGATNRPDALDGALRRPGRFDRELLFPLPGLQARRSILEIHTRKWSLRPSPALLDELAGLCVGYCGADL
KALCAEAALHAVRRRYPQIYASEDKLLVEPSSVNVEREDFLSAIAAVTPASNRSAAALARPLAGAAAVVLAPQLAAVLGRLQRSFPPAAQCL
AAGARREGGGSATTSPEPTASSILPPGRAGPSGSTAAGALVPRLASGTDGGAMGSLLARLPAWLMSRPRLLLCGPPGHGQGALASAVLYALE
GLPSHAIGLPALLANPGARSPEEALVLAFVEARRSAPAILYLPHLHLWWRTAPPTLRATLVMLLRDLPPELPLLLLATAEVPSCELDRELRDRV
FEGPGDVVALQTPGEQARRELFKASGAREPILAAAEPLDPALLAATRSAAAAGSAAAAAAAGGAEPAGPLPKDPSMEAARAAEAAAAEEL
AARRAYEDDQSALRALRMALRGVTLTLLNDRRWKMFAAPADAGEDPEYWQRVRREKEWASNVGSDWKVTNPMDLATLLARVDGRQYST
TSHYLADVQLIAQGARQYFGDDPQGVRQVSRAVALGDEAEHLVKSRVPMELAQRCEHMARRGGPAPPPPGLELPEELAAAKVKAAAAGA
KGKDGVGAATHGAAAAVNGAGGIVRRQRGGHGTGSDANCILMFDDPEMLLRRMSKQQLQQQREREQQDDLVEWTYQIQTAAFGAVLLL
NLLNLGMRQHLLPFKCNLLLLFINGVAFATDLLLWRGASPVLTSSAGRPFAPLRYV**Q**WCH**S**TPTMIY**M**LALLADCAGWQLVPPVLADVAM
VLTGLGACYTEGLLKAVLTLLSFITFAYVLVYVYGLFNKTISGDVSSDQRITLRTVLVLLLALWSAFPLVWLAAE**L**HLISPAA**E**AVAWGVC**D**YL
A**K**VVFSSQLWQSNLSGVQLRRDRALEAWEASNRVEAVARLTALLRQRDDLLSTLSHELRTPLAGIVALSSSLCKDPSAVSPASSRTLASVRAA
AACMLNIVTATLDSFAGRREGGEGRASKDFGAGSPAVAAGAAVEAIAVAAVDLQSLVETVVSVLRPLAHDGVQVVTDLPSRLPHVLADATR
LSQVLYNLVGNAMRFTSQGEVRISARILIPEGAELPAAAAGSALTTSGMGSSGGGITGCCSGGCEPPCGATRSSNSGSCSGRVSGSGGSGLWRG
RARRLSSSASSTSSSASAASPLPPGSEVEVLVEDTGVGMPQETLDELFQPYRSAEEGGVAAGVVAAKRRKATPLGGTGLGLYLVRLALRSQGSD
IVAESVPGLGSTFRFRLPVVDESTAARRDGASGAAGHRGALAERANSRLIGVSLVDAGGAPGTPPARPLTGPAVHAEAPSTALRKQRMSLDE
QREQRNPSLLRVMGRDRAASSSARSVLGGPVSAGGQSAGDTAAMAAAASAMNPPAAAEPQAADPPAVAAARAGRPQAARAPGPNFRHR
NNGTLQVMSVDDDPINQLVAGQMLTSQSWKVVKCMNGPEALKRLHLDPTTGASAAGSGAGGVREDCGEAALAAAVTAAAGAASLVLPD
CILLDVMMPGMSGFEVCRRLRERYPTAQLPVIIVSAKGDAAAVEEAFAAGADDYISKPYKRDEMVARIKAQPSAAGCSTTPEPAALAALAPA
DFPCVGGSAGGGAGSSGAGSSSNNGVAVCASGQPIALLQEVLAEVRRRNHVETELAKLGARVASLQAACGSLASDRDGWRRQARELRALL
AAGCNGAGRAATGCAMSADGFGGCNGGTSGLDTLLSAEPVRFASTNKLNLTSPGQGNGNGRNSDGSGASSVVNDLQDGGGGAGAGGVN
AAAGSGSRAVTGGGSATGGISGAVELQLQDLRREAALMRQQLMVALASVGTTRTDNSTSIGAVGGPVANSGAAEPGAGRMVQALAPDGSA
AAARVAEIGPGGAQPAGALPRRPPAPVAGGHNRAMHDLMLATNASDLSTRSGTGASMGSGMPGHAGSGAASAVADRPAAPVIPIPPPSNP
GKDTGPPVAATRHHLNPQQYP

>CsRh1_PRW60699.1 Chlorella sorokiniana

MPASKGSMAGHSAQRTGLHEPIEELPDRRLHFSRSVTLVSLCTMSYAFWIFYLCGHSESEATLEFYRVVPPTVYMWPMVAFATAVLLNLLSMA
MERRPVKRQLCFLNVYISGVAFVFEFLAWKQSAPIYINAAGRPTSLMRFV**M**WAH**A**TPIMLY**T**LSLISDFSTKQLYQTLGVNVFMIVAVIPGELIP
AWHRWIWNGLSCAVYPYIFHQIFYMYSAGIREARETGAQTSLRTLRNFTLSFWTFFPCVWVLVQ**L**GWVDLYH**E**EVLWCVA**D**TMG**K**IIFSSTL
LHSNFMSIEHRRMMAMRVVEEANRIRVIHELRQLVEQKEQFIALMSHELRTPLNGIIGLSNVLLMDVDSLPPDTCKTVTTIRNSGARLLNLIND
ILDAAALRKGKLAVQQGKVNLKNVADDVVDLTEALAKPGVNIRNKITADAPLVVGDTSRIVQVLYNLIGNAAKFTERGDIWVDATSSPDGA
TVAVSVHDTGIGIPEDKLDDIFAPFAQVDMSTTRRYGGTGLGLNLVKQLVEAHGGSISVASRRGKGSVFTFTLRVYAQDDGGSPGPVTAQQR
HSLALAAQEAADAAAEELDEVSMPNPEEEPLLASGRMSRKDSEPDMMLRLSHDAQPANMLQRLSADSRLRSSYDADYANYHKAPQQLSPQ
AALNAFLSAGPHQVVKEIQKGNITKPEDVAAATNGGDGNGGNGGSGGNGSQGDVEEEGPLAHDWLLADQVVQRQSSAGQVKVLSVDDD
PVNQMVIQAMLGKAGFKVLKAADGQKALDLLEESLRQGDPPHVMLLDVMMPGLSGYDVVRLVRERHPTLMLPVILVSANSREEHVVEGLQ
AGANDYVTKPFGRNELVARIQAQLRTREFSQGVRASLAAACQGLQQQQQQQAPQQGADGSLGWEGNRRLSLPVVERAASAPPDGLPPWP
CMI

>ApRh1_XP_011395544.1_Auxenochlorella protothecoides

MVAFATATVLNLASACLELRPIKRQLCFLAAYITATALVFEALAARGRAPIFWSVSGRPVSLLRYV**M**WAH**A**TPVIIY**T**LSMISDFDSLRVWRLV
LVDLVMIVTIIPGELVDAWHRWIWNIISFAVFPYICSELWAMYSSAIHASRDHATVRALSALRASTAVFWTLFPFVWTVAQ**A**RGVSTAW**E**ELL
WTCC**D**LGG**K**IIFSTSLLHSNFLTIDDRKMRAMREVIESNRVRVIQELRTLLEQREHFISVMSHELRTPLNGIIGLSSTLLLDLAGEPLRGRGPAVT
HDVAAIRSSGVRLLNLVNDILDASALREGKLAVKLERVEVRRVAEDVLALVRQLAAPGVVILNDIPSDLPAAKADTGRLIQVMYNLIGNACK
FTDKASWWWWWWGGGSKGDGAWCLGCIMLTAGPERENMVSISVSDTGIGIPPSKLDLIFGPFAQAGGLADMSYTRRYSGTGLGLHLARQL
LAAQGGTIHATSHQNKGSTFTFTLPVYTDADSKAAAVAGVISQGRTLRNPPRIDDMLEEEAGALGLGAQPDGGAAGEDLLQLLLGQAGLDP
GARGPGSAPRVGRSGLVAGTPVCVDNFASDFQTDMLDRLSLESRSTYFLATERETQRKAPQQLSPEAAASAFTSAAGPSPGWPGAQGDWAA
ADAGACGAGGASGDQAMSPFLLPSASTAESGGPPSGSDRGPLVLPPRGGGGAESRPDWQRAWQSVEDMRQRGAIRVLSVDDDPLTQTVVQ
GMLSRSGFQVSKAADGERALDVLEGGILAGRPPHIVLINLMMPKTSGFDVIRTIRSRWPGLMLPVIILSASCWEKAVVEGLQASGEVRSIKLAPL
PFQAGANDYLPKPFGCFELMARIEAQLRTHEFMAEACRQARPSEARSSEGGSGGSGSASANGVCSPDSSSDASYN

>AsRh1_jgi|Astpho1|75682|estExt_fgenesh1_pg.C_000730034 (5151)

MPGLRGWTGVLGWVGSAVVLYITLDNAAQAYWGDGSPEAAALLFAEVPQQWFRYSADFFLAALFVSLLSVLYEDSPAKKQAAVLSCLIKSV
ACYADWKLSVDGKGVIWDSAGYPFVVKRYV**N**FMI**T**TPTMLY**L**LSGVSSFSVGEVAATAGMQWGCVVTGLIASLTYGFWKWFWVTLSFAFYG
LVLREMQRMVNSAAKANEGTKMRLRGLNFIHNYSVVAWSLFPAAWVTG**H**LHLTSIGD**T**ELLMMAG**N**LVA**K**LLYSSGIMYDNFVTVSERRD
AAAAEIEQRQRLVMITELKSAMQRKDSFLSVMSHEMRTPLNGVIGLTDALLKGSSGKMTEKAVKLLHTIHDSANYLLNLVNDVLDAAAFRH
GKLKLMVQQVNISELVNTVFSNVLTMQQEGVKLHAEVDPRMPIIIADKDRLTQVLYNIVGNALKFTSMGTVGVLVKPFADMKSVVFTVADT
GIGIPEKNFSSIFLPFEQLDMSPTRKYRGTGLGLSICKVLVEAHGGSIAVSSEVGAGSTFTFTLPVEPPQPHSIRGDGRLSNDGSLLPKPEEGSPSRA
SLQLANPEKAISAGTGGSVAAGKGPLSPLGGIGTPSAGGATESKLKVSLHHVASCQALDRRNSSTTPHSRHAYTLDHVKPLHGQENGTFLVLS
VDDDPVAHLVLREMLTPEGYEVHSELDPQKALQWVAASPFLPDIVLVDCMMPGLTGYEFTAKLRETVPRTLVPVIMVSAKAEGTLIKNGFDV
GCNDFVSKPVMAEELLTRMATHLDLRQDAPGWMQIITGGPSKLDNEALRLLRSILPDRIIRRMKEGQTLIADSHPSVCILFADICNFTPLSSSMA
TAEVFLLLSNLFSLFDKLTDRHGVYKVETIGDCYMACAGHDEDDDKKAKGSPTQRMLAMAVDMLQAVQDLTVPNGGSVRVRIGMHVGPA
YAGVIGQKCPRYCFLGDTVNTASRMESNGFPMTIHLSDAAHRELLMCMDGSSFAPLGKRAIKGKGTMETFLAKEGEWRAAVEATQHPKPGK
LSKMRSLLPLIPALEARGGDASVQPGMAGDVHAAAKQAANAGLPALAASSRHAANAEGAAASRMASESPAAVLSPSRDGSQASTLQAQLE
AQQHALAAARATADAQLQQMLRVQGQLQAEILRHQMQRDQASGSTSRVSSFSALPGADAMAHLNGGGALSLAQLQALAAAGAMGMD
MYGNAYPTTPRGYPGMYPPPGGYYWPPPGPYWTPGPSSVAGAPAAGAGPVPVPSSVDLELRLNSAPVASTRTSVDRGSGDASGNPGSPDKTP
AGWTRKLFKKVGKSGHHAPPRNTSLL

>AsRh2_jgi|Astpho1|77589|estExt_fgenesh1_pg.C_001370002

MKEGTRGWPITLVVAGASVGAYYGLNQLCLKWGANDKAASAALFKEVPQIAFQRSGDAFFACFALHILNIAYENTNTKRQLALLAAVIKGI
AGWVDWQIAADGGGIIFDSSGYRPLVIRRFL**N**YAI**T**TPIIVY**L**VSCVSDFSGWRVAGVAAMQAGVIAFGVAASLLPSPWKWFVVSMSVFLYVT
VLRNIRHMVGSALAVRSGANRENGGLQFIFIYTVVLWSVFPATWFATQ**L**NLIGTRN**S**EIISMVA**N**WVA**K**VVYSSNIMFSSFVTISERKADAQA
VAEQEERVHMIKDLKEAMDHKDSFLSVMSHELRTPINGIMGLSDALVRGTGGKLNEKGQQFLVTINNSAHHLLNLVNDILDAAAFKHGKLK
LKVEEVDIHKVVAQVFSTLSTLKKGDVQMWNDIDDSTPNIMADSGRVVQVLYNLLGNSLKFTSKGSVGLHVVPSDDKKDVLFKVQDTGIGIA
EDKLQSIFVPFEQADMTTTRKYGGTGLGLSICKVLAEAHGGQIEVTSTPGLGSTFTVRLPIFPRQVKDAAPPPTGAVVAPPQTSPVLKVYTLDNI
ESASPAAAEKQPLLAVPETPRGEKSHKLSFAEAPGVTPPRSFDRQRPEMFTVEDQTLPQPVEQSPPPPVEQTLQTAGPQLPAVKTKGTRDRTVS
RSASFKGTSTPTTRTPTYTLQDVKPTHQDELGTILVLSVDDDPVAHMVLAEILEPMGYKLHCETDPEKALKWVNESVVLPDIVLLDCMMPKM
NGHQFCAKFRETVPRSMVPVIMVSAKTGEANVVEGLRNGCNDFLSKPVHRDELLARMETHLHVKQDASWMTHLINSGCSKVDNEAMQLL
KSILPDKIIARIQGGQKFIADSHQRVTVMFSDIVGFTPLSSSLPTAEVFMLLSNMFNTFDRLTDQYGVYKVETIGDAYMVVAGKPWHDEDDMK
RSLGTPAQRVLNMATAMIDMVKALTLPNGDFVRIRLGMHSGPAYAGVIGQKCPRYCFLGDTVNTASRMESNGFPMTIHLSEAAHKELVPVL
GDDYFVSVGKRNIKGKGTLQTYLAKEGMWEEAMQAIKEHGQEAASVVEVATPRRLSSRTMTMSRHQLQVAALEDALSTMRAALETEQAQH

LESQASIRQAQETIAAMQAQAAGQEENAKEQAAKLALLQANLEAKQQELKTCEARLETAAEKVRRMEAELEMAQRKVEEAQKQVQDAHK
DAVERQGELLRLKNAAAEVEIEEVEPAEEPAVPERRAAKADGQRQRAAQFPSRDPPGQPQERPRSLSRPPARDEEGYLDDEGGEDGHPPRRF
QEESSPPRNARAMSVTPPMSVTEWDDQPQEWDGPATIPARRPGHGESPMQDYLGEHSALLQEVQRKRMQRQGEGYGAYASSQPGNGGSVS
PWGGKSNYTSASADEHLRGMFRAARDMAPTRHSPEGYGWEDADHERGPSPGRRGGPRPLDTMYDSIDPRGYRSPGYRSAVDGNAGSYSPD
GFPAKAARGRPTSARSHPRTPSSPQRDRSGWGPAY*

>AsRh3_jgi|Astpho1|29226|e_gw1.00088.7.1 (6282)

SQVPKQHYQISVASFLSALVINFTSLLYEAGATKKQLALLSCFIKGAAAYADLVTGTGQGIILMDPNGTPCVVNRYI**Q**WAL**T**TPTLVY**V**ISRISSF
STKRVALAVGAQVVVIVTGLLSQLSPGIFFWLYICMSFFFYVFVMIEMGKMVGSVLTEESNSEARSGLLFIFWSTVVVWLAFPIYWCCQQ**L**GILS
VYN**C**ELGTLVA**N**FVA**K**VLFSSSVMYGNFLTIAQRRALAKEAAEHANRLKLIEDLKASMKVKEEFLSVVSHELRTPLNGVIGLSEALLRPGPWQ
LGEKGLHYIKTIKNSSHHLSNIINDILDAASMAKGKLVLKTERVSLNKVVDHVFDIMGHLVKKKVTLEKNLAPDLPHIIADGSRVVLYNLLGN
SVKFTTEGRITVSVKPMPGSLPGMEMLQLSGDMTAARKFGGTGLGLNIVKRLVEAHGGTINVESTPGVGSTFTVRLPVKPPESQAQKQAEGSV
GDWSNRKNMQQSTPRNSGGEDQRSTDVGSPCLTERKPTHMEEYGNTLILSVDDEPTNHLVIEEALESAGYKMHSELDGKDALAWINGSKVL
PDLILLDCMMPNMTGEPAAWHEFCIELRKTVPQAVVPVIMISAKNNEDNVVEGLSHGCNDFLSIVNGVLQEDTEAMQILKSILPECIIARMQD
GHAYIGDSYDNVAILFSDIVGFSTMASTMSAVEVFLLLTNLYTQFDRLVDKYAVYKVETIGDGYMLAAGHDEDKDKARIGTPMERLMMMAK
AMLDCAAKFRTNNGAALQIRVGVHCGPVYAGVIGSKCPRFCFIGDTVNVASRMESNSFPMCIHLSEVAYKNLGSHMSQAVSMGKRAIKGKG
EMVTYLAKVGSMLFFCCVLICLSGWHSVVLRIRARQRPA*

>AsRh4_jgi|Astpho1|76150|estExt_fgenesh1_pg.C_000880024 (6192)

MLAQLQLDAQALGSPTSPIEFCKSVDLPDQHFLAYGAGAGLVGIATCQASSDSPSQPGVPFARQPDIVLAVPAARNAGRDIISVVFSSKSAENL
LAAAAGNHIHLFSLTASMLALQGRIAPHEGPPQGDSQMLCAVQHSADIAALSWTQAGDGLLAADTQGCVVMYRVPEPGDLSGRGSRLVHV
WTGSSSMLVQPQTLLAAGTNVMSPAASASPGSRQVTIWWPEHLPDVQDAAHQRQAHHRPSPEQVAVLAEQLRHPAGVVGLEWSPGALQH
GVSPDLEEEDGDEAGSPVAAATSQPALMTVGEDSVIRIWVEVTVGPMLGPAQGSSLGRPALPQQPAGPAAPGGGSGGARLQAPPGKGEPRR
CRAKGAQVSRDAVHFLGGIQQWSKEEPRWAQALCTTLYTSGSGARGFKLQAQLAPGTLRACWGQPDAGLAGVNHRLQSSQIMWVLAVAQ
QRALEGSTEPQSEQLLLWALDGLSGVVINGIPNNLPGTRAASVPKAVLWGQHCSALVWPKAAGQRRLACGCSTEHAAPVLQVLESCRGEGS
DTLLRTFELHTVDEVTQLHVARRSRAGISAGNQPLAMVHELPLHSLTLQRQTGHAAPVTALAQHPRQPLAASLDANGLVLLWSVPAGAPLR
LLGSLSELSGAVQAPEGATAVVWLDPAAGGAQLAVGGAHGVDIYGVSRLGEQQQTPLQVAHLAVARLPDQWRGLEHLTSLAGRPGGRQLL
LGLQQQPGTATPQSTPPPVAAWQMASQATCMAAAPQESGFDLLLGGSDGLLRLVTLGDRNSLLEQDRLVCSASGQGQPTGIPVAAAASGGS
AWLAAAVQSFGSADHLVCIWQATVASDTPSFSLRAALPLPEAPTAVVWLEGCLPGVALAVADSWPEFRVFVRGKDGGFAPIAKLPSLPDSVR
QLCGANTGRLPGQQPLAGASAGEDAGLGTPALAAAGTKPKAPATPGSSAPPGSAPQALCQQVLLGAGSLLACLSPVARATEQPDGHGRVL
ALVGQEVSGGSPVALAAVAAEAGGPLPQFDPRRLRMLIERGRMQAASAAIRVMLQRLQGRSATGKPDEVPGADQKLPPPMVDAILDANFLK
AFTRLSARLPESAKAAAAQAAKAAPAAPAAAARAGSGSAQTDDDDGPMKPLHTSKGTAPNGELKGTAGSTGSRAASALPVDPFAFNMGAF
DMGSSSAPATETRADQQPASADPYAFDMGSFGGDPAPAAAPPPQQAPTAADPFAFDMGAFGMGTDSQQEAGHQTKSQPGPPPASSGDPFA
FNPDAFGMGDAAGSGHAEQPDSAAPVPAAAADPFAFNPEAFGMGQPPQQVPGSAQAAPAPGAAADHFAFDPEAFGMDQLGQPPQQPPG
SAQAGHGRDSAASNEDISVKGPQRQGAASVPGSRTTGTSLPGAAQSPARDRSQRRQQAAAVFQPRRPGSKPADALSLEELEALERLLLPQVD
PSDDEDDLAGQADKSQQMPFRSPWATGEATAALGLTHQQALEVFELAQLLCPSAAQRALPPTPGALEPAAGALPSADWAALDAAGQQLL
RRLQLALVWQHHIAAEQAAVAGAPGGAGAGRSREVSDGLMASAHTNNALLGSVTEAASWAERVGLTPGLSSEGAMWGVLSESQEALLTD
SLKLVPGSLDQEAAEKPMEFGFSTGPARKQPRHQLSWEGMRQAGAGFWLTDPQVVKSTAEALAKAQYAATKDPHSCALMYTALQRKALLT
GLFRTANNKKVADFLGRDFSDVKNRTAASKNAFVLLGKHRHELAAAFFILAGSNKDAVGVCGHEMRDPQLALFLARLLEPGNPQLQQEIID
DRLLPDAQEAEDAWAVCLLQWLHGQPAAGLLTLVTSNHLMSSSDSGTPSQTPSRTLRQPQKEQALVRSASRRHGNVPRQAPSLPSLDNVFA
LDLILFCLQDNLNVWPGPLPSLPELHSLATAAAQQADVQGLPSAGLEALHVASSCMHRLQGAAGQGQRHAAVLGAWQQRLVLAVLVQGI

DWQPLTALAQGQDSRPGSAMRKALTAHTAAWQDQAAALLARLDAAGIAVDQQVALQQLAIWEAALQAAVLPGLGQPGKAEGDRLSRSH
SAFSGKSLISTRHSVLGPAVFEASHELLHVEGDRLHAVCASNAGMYGYAGGEGRPFVAASGHHGLIQGEVLLLDEHYSVGADREEGLEDQRK
GSAFSALLGDMLEAVRWPGDPWTNMATPHMGGGVLAGGPHTRAATAPKPESPSAARVRAPLGQILTSTLCAHPTRQLYLSALMSGSSSGEV
FLCKFGDRFATAGYTPMPAAVTIAPSSAQSMFSAPPRSWNPAPGTSFTHWGQPQSVVWSDCGERFAGIGDGGVVATWRIDAPRLGLSKRGM
DVAFLGTGSSVLAVGGLDASGANIAIWDTQAPSYHRPVARLNHHSAAVTSLQVLPGGYHLASADASGTIAVKDLRMLGGSLANSASPRGSS
ARSSGHLMAGSPRKRLSVDGSASSAAADAAARGLLWLVRPPSESAGVVCMTAGLWPAPDGSAGGTMLVTGHHDGTIHVWSAGRGRHLQA
IEGAHVMPSSQQQTLTSKDITTLIVAIAASAYFAMAAGGGDDYFPVYYNNAQSRDIFWARYI**D**WFL**T**TPLLLL**D**LLILTDITVATTIWIMAADIF
MIIFGLFGAVSDHKTKWGWFGVACFFEALIVVGLMLPGMKGAFARGKSTGMLFAIMAVYLSVLWWGYPIVWGLAE**G**SNTISADA**E**IGAYAG
L**D**IVA**K**VVFGWIIMLLGYPMIGKQMNMEHEQGRFLPQLLSSPINAPLSIVPTGAKAYHPVGKSNPGGPMESATGATTGSAVPAGATTASAGT
KAPGANGQTIV*

>BgRh1_jgi|Bigna1|89464|estExt_fgenesh1_pg.C_490145

MSTMRTSVRPKIGGHIQVGDEGHQGYGHIEKLLYPFDVLASFIETVFPNMQKYATTTGIVIVCYLAHARMVSTYPTDGVVSVVADVHTTIVVS
AAITTFFFSVSPFYALFWERATKEGEHVGIYSRSHVGFLCICGVTFACMATHIYIAAIGQIHTLNTLGGSRVFYTTYYC**E**WIT**T**TPMMIQ**S**LFHFA
EMTKRRRIVVIRAQILNFIMFTSGSWLWGKPTLLKGVIVAYFFIGNLYSQDRLLKFSKVVSRDEPLQREWDTYNSNLRIFYTMTWMLFPMIYVL
GS**L**EIIQPWM**E**IKLFAYA**D**LSA**K**VGLVNMAYRYINNATEEIAWCKKILEQERLKSDVVRYIFHETRVPLNTISLGVSNLDVSDDAKEIESVKKTL
AASCDSMRHILDDYLVWEKMRAGKISKFKFEVATFALQEIVDMLLTRFKATAEKNDLTYTVEARVKNTSFIGDKYKIVQIASNYISNAMKFTP
RGGIVSFVVEEIQPPPNSSYRPSNLHSSIRDALEEGDICWLQFSCRDTGAGISEENQKLLFTPFVQIEPGVKKNSSGAGLGLSICKEMAAGMGGSV
HLESMIGKGATFYVTLPLPKAISRKVIPDGPLQATIGDGNKLSEEAPLRVLVTDDVKSNREFLAKRLRRMKKENGKPKYVCDVAEDGVAAIRM
SQNKGPFPYDVYLMDNQMPNLLGRDCIRKLRDEHGIKSMMIGVTGDIISEDRKLMLECGADCIHGKPVNVKALTQDISAFFEKKRNASQSKSS
G*

>BgRh2_jgi|Bigna1|139324|aug1.49_g14032

MSTMRTSVRPKIGGHIQVGDEGHQGYGHIEKLLYPFDVLASFIETVFPNMQKYATTTGIVIVCYLAHARMVSTYPTDGVVSVVADVHTTIVVS
AAITTFFFSVSPFYALFWERATKEGEHVGIYSRSHVGFLCICGVTFACMATHIYIAAIGQIHTLNTLGGSRVFYTTYYC**E**WIT**T**TPMMIQ**S**LFHFA
EMTKRRRIVVIRAQILNFIMFTSGSWLWGKPTLLKGVIVAYFFIGNLYSQDRLLKFSKVVSRDEPLQREWDTYNSNLRIFYTMTWMLFPMIYVL
GS**L**EIIQPWM**E**IKLFAYA**D**LSA**K**VGLVNMAYRYINNATEEIAWCKKILEQERLKSDVVRYIFHETRVPLNTISLGVSNLDVSDDAKEIESVKKTL
AASCDSMRHILDDYLVWEKMRAGKISKFKFEVATFALQEIVDMLLTRFKATAEKNDLTYTVEARVKNTSFIGDKYKIVQIASNYISNAMKFTP
RGGIVSFVVEEIQPPPNSSYRPSNLHSSIRDALEEGDICWLQFSCRDTGAGISEENQKLLFTPFVQIEPGVKKNSSGAGLGLSICKEMAAGMGGSV
HLESMIGKGATFYVTLPLPKAISRKVIPDGPLQATIGDGNKLSEEAPLRVLVTDDVKSNREFLAKRLRRMKKENGKPKYVCDVAEDGVAAIRM
SQNKGPFPYDVYLMDNQMPNLLGRDCIRKLRDEHGIKSMMIGVRKPEEEEEEEENIEKTKQKLRIRYVYYSILVQVTPRSPYLVLSPDNPKSFL
SRSRETLSLRTGS*

>1KGB_BR

QAQITGRPEWIWLALGTALMGLGTLYFLVKGMGVSDPDAKKFYAITTLVPAIAFTMYLSMLLGYGLTMVPFGGEQNPIYWARYADWLFTTPL
LLLDLALLVDADQGTILALVGADGIMIGTGLVGALTKVYSYRFVWWAISTAAMLYILYVLFFGFTSKAESMRPEVASTFKVLRNVTVVLWSAY
PVVWLIGSEGAGIVPLNIETLLFMVLDVSAKVGFGLILLRSRAIFG

>1UAZ_AR1

TAAVGADLLGDGRPETLWLGIGTLLMLIGTFYFIVKGWGVTDKEAREYYSITILVPGIASAAYLSMFFGIGLTEVQVGSEMLDIYYARYADWLF
TTPLLLLDLALLAKVDRVSIGTLVGVDALMIVTGLVGALSHTPLARYTWWLFSTICMIVVLYFLATSLRAAAKERGPEVASTFNTLTALVLVLW
TAYPILWIIGTEGAGVVGLGIETLLFMVLDVTAKVGFGFILLRSRAILGDTEAPEPSAGAEASAAD

>1VGO_AR2

QAGFDLLNDGRPETLWLGIGTLLMLIGTFYFIARGWGVTDKEAREYYAITILVPGIASAAYLAMFFGIGVTEVELASGTVLDIYYARYADWLFT
TPLLLLDLALLAKVDRVTIGTLIGVDALMIVTGLIGALSKTPLARYTWWLFSTIAFLFVLYYLLTSLRSAAAKRSEEVRSTFNTLTALVAVLWTAY
PILWIVGTEGAGVVGLGIETLAFMVLDVTAKVGFGFVLLRSRAILGETEAPEPSAGADASAA

>1H2S_SRII

MVGLTTLFWLGAIGMLVGTLAFAWAGRDAGSGERRYYVTLVGISGIAAVAYVVMALGVGWVPVAERTVFAPRYIDWILTTPLIVYFLGLLAG
LDSREFGIVITLNTVVMLAGFAGAMVPGIERYALFGMGAVAFLGLVYYLVGPMTESASQRSSGIKSLYVRLRNLTVILWAIYPFIWLLGPPGVAL
LTPTVDVALIVYLDLVTKVGFGFIALDAAATLRAEHGE

>1XIO_SRI

MNLESLLHWIYVAGMTIGALHFWSLSRNPRGVPQYEYLVAMFIPIWSGLAYMAMAIDQGKVEAAGQIAHYARYIDWMVTTPLLLLSLSWTA
MQFIKKDWTLIGFLMSTQIVVITSGLIADLSERDWVRYLWYICGVCAFLIILWGIWNPLRAKTRTQSSELANLYDKLVTYFTVLWIGYPIVWIIGP
SGFGWINQTIDTFLFCLLPFFSKVGFSFLDLHGLRNLNDSRQTTGDRFAENTLQFVENITLFANSRRQQSRRRV

>1E12_HR

AVRENALLSSSLWVNVALAGIAILVFVYMGRTIRPGRPRLIWGATLMIPLVSISSYLGLLSGLTVGMIEMPAGHALAGEMVRSQWGRYLTWAL
STPMILLALGLLADVDLGSLFTVIAADIGMCVTGLAAAMTTSALLFRWAFYAISCAFFVVVLSALVTDWAASASSAGTAEIFDTLRVLTVVLWL
GYPIVWAVGVEGLALVQSVGATSWAYSVLDVFAKYVFAFILLRWVANNERTVAVAGQTLGTMSSDD

>Sodium (Na^{+}) ion pump (6RF6_A)

MTQELGNANFENFIGATEGFSEIAYQFTSHILTLGYAVMLAGLLYFILTIKNVDKKFQMSNILSAVVMVSAFLLLYAQAQNWTSSFTFNEEVGR
YFLDPSGDLFNNGYRYLNWLIDVPMLLFQILFVVSLTTSKFSSVRNQFWFSGAMMIITGYIGQFYEVSNLTAFLVWGAISSAFFFHILWVMKKVI
NEGKEGISPAGQKILSNIWILFLISWTLYPGAYLMPYLTGVDGFLYSEDGVMARQLVYTIADVSSKVIYGVLLGNLAITLSKNKELVEANS

>RhGC (blastocladiella emersonii)

MKDKDNNLRGACSSCNCPEYCFSPTSTLCDDCKCSVTKHPIVEQPLSRNGSFRSSGASLLPSPSSPNVKITSTVGLRSRKSESQANVRGSMISNS
NSGSRSNNSGGAGGGSGGSSSSKGGSALANYQSAMSELWSWNMMLSTPSLKFLTVQFTTWIVLTTVGA**IYTL**FFHERQAYNRGWADIWYGY
GAFGFGLGLSFAYMGFTGARNPEKKALSLCLLGVNFISFMSYIIIMLRLTPTIEGTMANPVEPARYL**E**WIA**T**CPVLIL**L**ISEITQYPHDPYKVIVND
YALCLAGFVGAISAQQPWGDLAHFVSCLCFSYVVYSLWSCFTGAIDGETQCNVEKSGLRWIRFSTITTWSLFPITWFSYT**S**GLISFTV**A**EAGFSMI
DIGA**K**VFLTLVLVNSTVEQAQNQKVDAITAIAEELENQINNCDAILQKMMPEGVLEQLKNGQATEAKEYESVTVFFSDITNFTVISSRTSTKDM
MATLNKLWLEYDAIAKRWGVYKVETIGDAYLGVTGAPDVVPDHAERACNFAVDIIEMIKSFKTITGESINIRIGLNSGPVTAGVLGDLNPHW
CLVGDTVNTASRMESTSKAGHIHISESTYHFIKSKFVTQPLDVMEVKGKGKMQTYWVLGRK

>Rh-PDE

MGRKNAANSSMLQEASMNNYSMTSAASGASSSGRGKRRAKTRNIAIASTKEVQWQGIFMIIVWLCVMGSLIFFANPEASRRVFAKFSHLQSF
YGATSVAFAFATGLDILAYVNAVSDEKRVLSGILAYVDGVACISYLSMATLNLYFLVDSTQGNPVWLMRYA**E**WII**T**CPTLLY**W**CGLASRADRS
SVSDIATADALLLAGGALSSILPSWPAFFVFAGSFATYIYVMLHMWGMFGKAMQPDFQPPPPLPRHALHLLRCEIVMSWSIFPLVEFLRR**Q**GYI
DFQV**G**EAMNCVA**D**YAA**K**VGLAMIMVNCNLEQINALRVQQMHSALTGMLKVMRKTNLSSSRMAQLDGVDDDVKSWIMNEFSGSTDGKG

GDDAKAQQRARGRKGHSAAMDAEKLAASSLFTWDFDALDKSDEDLTAVCVRVFQELHVIEQFNIDEKKLRKWLQKVRTQYNKNPFHNWR
HAVMVLHTTYLLLTSVATEFITEVELLAMLIAALSHDLDHNGLTNAFHINSRSELALVYNDQSVLENHHSHLAFEILLEKGCNVVENLDEDEF
KRLRAVIINCILNTDMATHHTKLSKMEEVNRLGGFINLAENNDQRLFVLAVLLHTADLHNPIKPFESNKRWSARLQKEFNNQVELEAKMNL
PSLPFMRGNDEESLAKGEIGFINFVVKPWHQQLSQAFPKLDFLLDTIDANLAEWKAIAESYRQMH

>GtACR1 (6EDQ) Anion channelrhodopsin 1

MSSITCDPAIYGEWSRENQFCVEKSLITLDGIKYVQLVMAVVSACQVFFMVTRAPKVPWEAIYLPTTEMITYSLAFTGNGYIRVANGKYLPWA
RMASWLCTCPIMLGLVSNMALVKYKSIPLNPMMIAASSICTVFGITASVVLDPLHVWLYCFISSIFFIFEMVVAFAIFAITIHDFQTIGSPMSLKVV
ERLKLMRIVFYVSWMAYPILWSFSSTGACIMSENTSSVLYLLGDALCXNTYGILLWATTWGLLNGKWDRDYVKGRNVDGTLMPEYEQDLEK
GNTERYEDARAGET

>ChR1

MSRRPWLLALALAVALAAGSAGASTGSDATVPVATQDGPDYVFHRAHERMLFQTSYTLENNGSVICIPNNGQCFCLAWLKSNGTNAEKLA
ANILQWITFALSALCLMFYGYQTWKSTCGWEEIYVATIEMIKFIIEYFHEFDEPAVIYSSNGNKTVWLRYAEWLLTCPWRDSRLNVILIHLSNLT
GLANDYNKRTMGLLVSDIGTIVWGTTAALSKGYVRVIFFLMGLCYGIYTFFNAAKVYIEAYHTVPKGICRDLVRYLAWLYFCSWAMFPVLFLL
GPEGFGHINQFNSAIAHAILDLASKNAWSMMGHFLRVKIHEHILLYGDIRKKQKVNVAGQEMEVETMVHEEDDETQKVPTAKYANRDSFII
MRDRLKEKGFETRASLDGDPNGDAEANAAAGGKPGMEMGKMTGMGMGMGAGMGMATIDSGRVILAVPDISMVDFFREQFARLPVPYEL
VPALGAENTLQLVQQAQSLGGCDFVLMHPEFLRDRSPTGLLPRLKMGGQRAAAFGWAAIGPMRDLIEGSGVDGWLEGPSFGAGINQQALV
ALINRMQQAKKMGMMGGMGMGMGGGMGMGMGMGMAPSMNAGMTGGMGGASMGGAVMGMGMGMQPMQQAMPAMSPMMT
QQPSMMSQPSAMSAGGAMQAMGGVMPSPAPGGRVGTNPLFGSAPSPLSSQPGISPGMATPPAATAAPAAGGSEAEMLQQLMSEINRLKNE
LGE

>ChR2

MDYGGALSAVGRELLFVTNPVVVNGSVLVPEDQCYCAGWIESRGTNGAQTASNVLQWLAAGFSILLLMFYAYQTWKSTCGWEEIYVCAIEM
VKVILEFFFEFKNPSMLYLATGHRVQWLRYAEWLLTCPVILIHLSNLTGLSNDYSRRTMGLLVSDIGTIVWGATSAMATGYVKVIFFCLGLCYG
ANTFFHAAKAYIEGYHTVPKGRCRQVVTGMAWLFFVSWGMFPILFILGPEGFGVLSVYGSTVGHTIIDLMSKNCWGLLGHYLRVLIHEHILIH
GDIRKTTKLNIGGTEIEVETLVEDEAEAGAVNKGTGKYASRESFLVMRDKMKEKGIDVRASLDNSKEVEQEQAARAAMMMMNGNGMGMG
MGMNGMNGMGGMNGMAGGAKPGLELTPQLQPGRVILAVPDISMVDFFREQFAQLSVTYELVPALGADNTLALVTQAQNLGGVDFVLIHP
EFLRDRSSTSILSRLRGAGQRVAAFGWAQLGPMRDLIESANLDGWLEGPSFGQGILPAHIVALVAKMQQMRKMQQMQQIGMMTGGMNGM
GGGMGGGMNGMGGGNGMNNMGNGMGGGMGNGMGGNGMNGMGGGNGMNNMGGNGMAGNGMGGGMGGNGMGGSMNGMSSG
VVANVTPSAAGGMGGMMNGGMAAPQSPGMNGGRLGTNPLFNAAPSPLSSQLGAEAGMGSMGGMGGMSGMGGMGGMGGMGGAGAA
TTQAAGGNAEAEMLQNLMNEINRLKRELGE

>VChR1

MDYPVARSLIVRYPTDLGNGTVCMPRGQCYCEGWLRSRGTSIEKTIAITLQWVVFALSVACLGWYAYQAWRATCGWEEVYVALIEMMKSIIE
AFHEFDSPATLWLSSGNGVVWMRYGEWLLTCPVLLIHLSNLTGLKDDYSKRTMGLLVSDVGCIVWGATSAMCTGWTKILFFLISLSYGMYTY
FHAAKVYIEAFHTVPKGICRELVRVMAWTFFVAWGMFPVLFLLGTEGFGHISPYGSAIGHSILDLIAKNMWGVLGNYLRVKIHEHILLYGDIR
KKQKITIAGQEMEVETLVAEEEDDTVKQSTAKYASRDSFITMRNRMREKGLEVRASLDAGGGDSGMEAGGGGAAHAQPHMAKPGTELGKT
MSASFTNGAATSLEPGRVILAVPDISMVDFFREQFAQLPVPYEVVPALGAENTVQLVQQAAMLGGCDFVLMHPEFLRDRGPTGLLPQVKMM
GQRTAAFGWSQMGPMRDLIESSGVGAWLEGPSFGSGISQAALQQLVVKMQQAKRMAAMGSMMGGGMGNGMGMGMGMGMGMGN
GMGNGMGMGNGMGNGMGMGNGMGMGNGMGMGNGMGMGNGMGNGMGNGMGMGNGMGNGMGNGMGNGMGNGMGNGMG
MGNGMGMGNGMGNGMGNGMGNGMGNGMGNGMGMMTPGAMGMGMGGMGNLAAAAGNAMYGGGGGGGGSTMGSGNAAMMTGLVMG

GGNGVGAGPGGVVANLGSSALQPQSQMMGGGNVVGMSSPQLQLQQSSSMPLGGLAPNRIGNNPLFGAAPSPLHSQPGASPTGLSSPQLGM
GAMLPAGTSVGAGGGSVGPTETDMLQQLMTEINRLKDELGE

>VChR2

MDHPVARSLIGSSYTNLNNGSIVIPSDACFCMKWLKSKGSPVALKMANALQWAAFALSVIILIYYAYATWRTTCGWEEVYVCCVELTKVVIEF
FHEFDEPGMLYLANGNRVLWLRYGEWLLTCPVILIHLSNLTGLKDDYNKRTMRLLVSDVGTIVWGATAAMSTGYIKVIFFLLGCMYGANTFF
HAAKVYIESYHTVPKGLCRQLVRAMAWLFFVSWGMFPVLFLLGPEGFGHLSVYGSTIGHTIIDLLSKNCWGLLGHFLRLKIHEHILLYGDIRKV
QKIRVAGEELEVETLMTEEAPDTVKKSTAQYANRESFLTMRDKLKEKGFEVRASLDNSGIDAVINHNNNYNNALANAAAAVGKPGMELSKL
DHVAANAAGMGGIADHVATTSGAISPGRVILAVPDISMVDYFREQFAQLPVQYEVVPALGADNAVQLVVQAAGLGGCDFVLLHPEFLRDK
SSTSLPARLRSIGQRVAAFGWSPVGPVRDLIESAGLDGWLEGPSFGLGISLPNLASLVLRMQHARKMAAMLGGMGGMLGSNLMSGSGGVGL
MGAGSPGGGGGAMGVGMTGMGMVGTNAMGRGAVGNSVANASMGGGSAGMGMGMMGMVGAGVGGQQQMGANGMGPTSFQLGSN
PLYNTAPSPLSSQPGGDASAAAAAAAAAAATGAASNSMNAMQAGGSVRNSGILAGGLGSMMGPPGAPAAPTAAATAAPAVTMGAPGGG
GAAASEAEMLQQLMAEINRLKSELGE

>MvChR1

MSPPTSPTPDTGHDTPDTGHDTGGHGAVEICFAPCEEDCVTIRYFVENDFEGCIPGHFDQYSSHGSLHDIVKAALYICMVISILQILFYGFQWW
RKTCGWEVWFVACIETSIYIIAITSEADSPFTLYLTNGQISPQLRYMEWLMTCPVILIALSNITGMAEEYNKRTMTLLTSDVCCIVLGMMSAASK
PRLKGILYAVGWAFGAWTYWTALQVYRDAHKAVPKPLAWYVRAMGYVFFTSWLTFPGWFLLGPEGLEVVTGTVSTLMHACSDLISKNLWG
FMDWHLRVLVARHHRKLFKAEEEHALKKGQTLEPGMPRSTSFVRGLGDDVEIDPSYELYRLKRQNHPEYFLSPAQTPRRGPSFDKRTSFEMD
GGKNGMLQMMPVTGMGMGMGMGMGGGKTVLFLDYTGGGYVSFFEQQLSNMGVNVTKCWSDDDMYNTAGVANVKQLFHFAMIPNNA
LGGQMVMDLRGTGLLVVAYGPEPPMPGMGQDEFVPLQMPGVPYDESILHNLVMRHAITQGLGMNGMQGNMGQQQQMMGMQGNMNG
MQGNMNGMQGNMNGMQGNMSGMQGNMNGMQGNSGMNQGWNNQGFTNTGAFGY

Publisher's Note: MDPI stays neutral with regard to jurisdictional claims in published maps and institutional affiliations.

© 2020 by the authors. Submitted for possible open access publication under the terms and conditions of the Creative Commons Attribution (CC BY) license (http://creativecommons.org/licenses/by/4.0/).
